# Supplementary material for: Mosaic Epigenetic Dysregulation of Ectodermal Cells in Autism Spectrum Disorder
Source: PLoS Genet. 2014 May 29;10(5):e1004402. doi: 10.1371/journal.pgen.1004402 (PMC4038484; doi:10.1371/journal.pgen.1004402)
Supplement: Figure S1 — H&E staining of buccal epithelial samples. The buccal brushings yielded the expected squamous epithelial cells (a.i-a.vi) with very small proportions of possible contaminant cells types (b) of different sizes (b.i-b.iv) or nuclear characteristics (b.v). Counts of 25 individual cells per sample showed high purity in both ASD and TD groups (c). (PDF) [file pgen.1004402.s001.pdf]

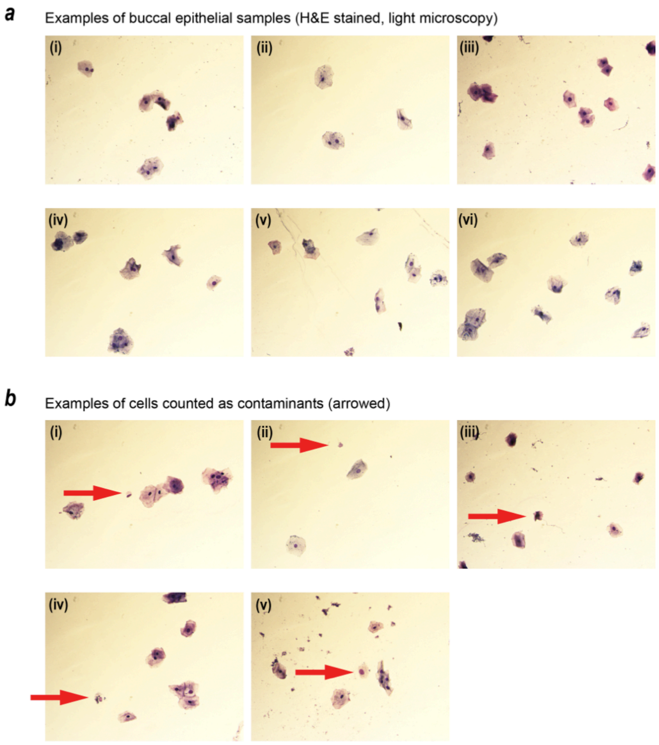

c Percentages of buccal epithelial cells in each cohort (25 cells counted per sample)

	Median	Mean	Standard deviation
Typically developing (TD)	100	97.96	2.35
Autism spectrum disorder (ASD)	100	97.86	2.52

Supplemental Figure S1: H&E staining of buccal epithelial samples.

The buccal brushings yielded the expected squamous epithelial cells (a.i-a.vi) with very small proportions of possible contaminant cells types (b) of different sizes (b.i-b.iv) or nuclear characteristics (b.v). Counts of 25 individual cells per sample showed high purity in both ASD and TD groups (c).
