# Supplementary material for: Mosaic Epigenetic Dysregulation of Ectodermal Cells in Autism Spectrum Disorder
Source: PLoS Genet. 2014 May 29;10(5):e1004402. doi: 10.1371/journal.pgen.1004402 (PMC4038484; doi:10.1371/journal.pgen.1004402)
Supplement: Figure S2 — B allele frequency plots of known mosaic cases. The MAD output correctly highlights an abnormality along chromosomes; analysis of BAF patterns allows determination of the source of error. The pattern in AG13074 results from meiosis I non-disjunction, and F44 P110 demonstrates a meiosis II non-disjunction with mosaic UPD. The lack of array probes on the p arm of chromosome 13 precludes definitive assessment of the meiotic source of the GM00503 error (I or II); the pattern of GM00682 could result from a variety of situations. (PDF) [file pgen.1004402.s002.pdf]

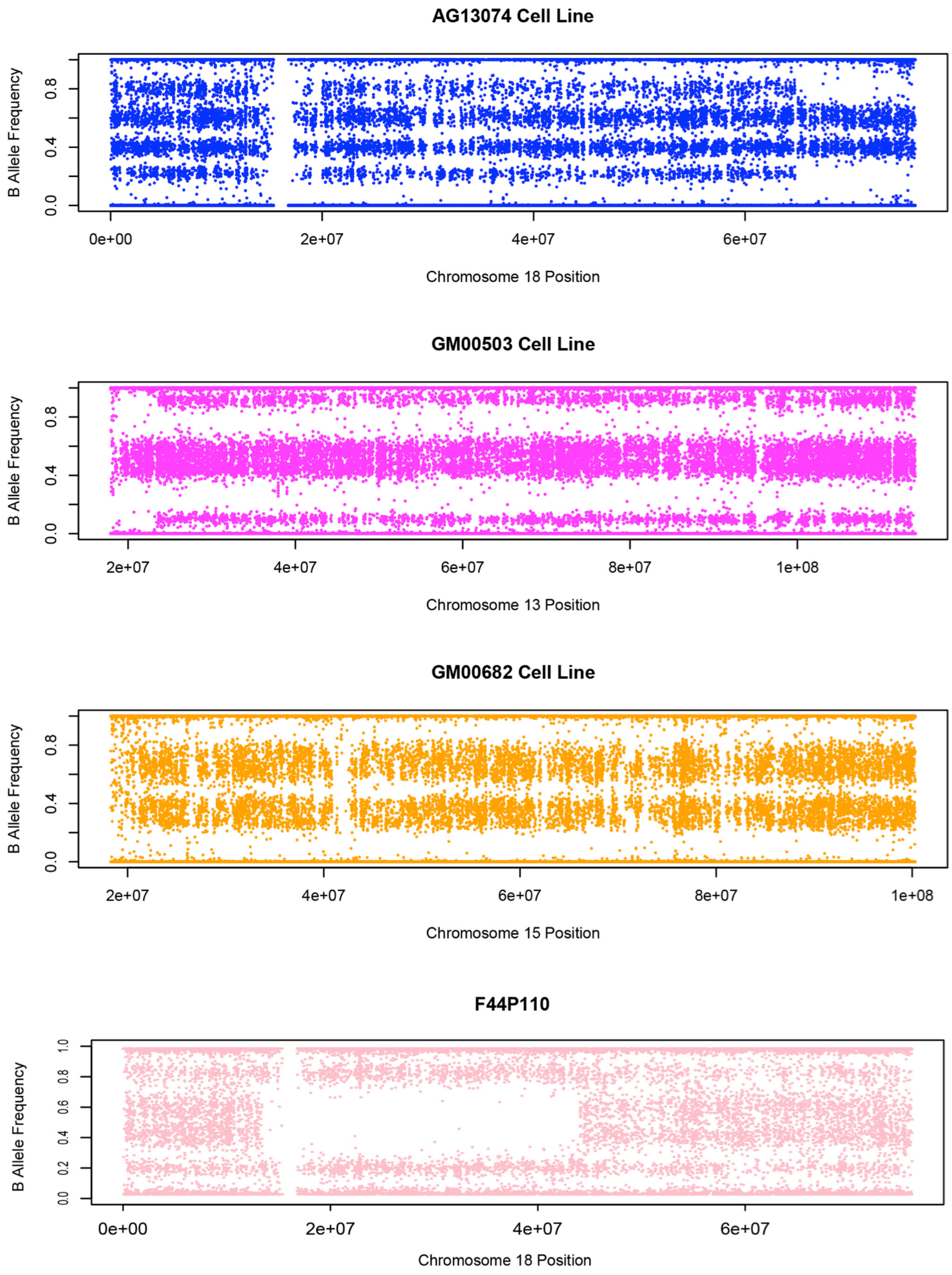

Supplemental Figure S2: B allele frequency plots of known mosaic cases

The *MAD* output correctly highlights an abnormality along chromosomes; analysis of BAF patterns allows determination of the source of error. The pattern in AG13074 results from meiosis I non-disjunction, and F44 P110 demonstrates a meiosis II non-disjunction with mosaic UPD. The lack of array probes on the p arm of chromosome 13 precludes definitive assessment of the meiotic source of the GM00503 error (I or II); the pattern of GM00682 could result from a variety of situations.
