# Supplementary material for: Mosaic Epigenetic Dysregulation of Ectodermal Cells in Autism Spectrum Disorder
Source: PLoS Genet. 2014 May 29;10(5):e1004402. doi: 10.1371/journal.pgen.1004402 (PMC4038484; doi:10.1371/journal.pgen.1004402)
Supplement: Figure S3 — Preprocessing of Illumina 450 K data. The colors in the density plots correspond to different chips (microarrays), each run to contain 12 samples. The normalization procedures correct for both intra-array and inter-array differences. (PDF) [file pgen.1004402.s003.pdf]

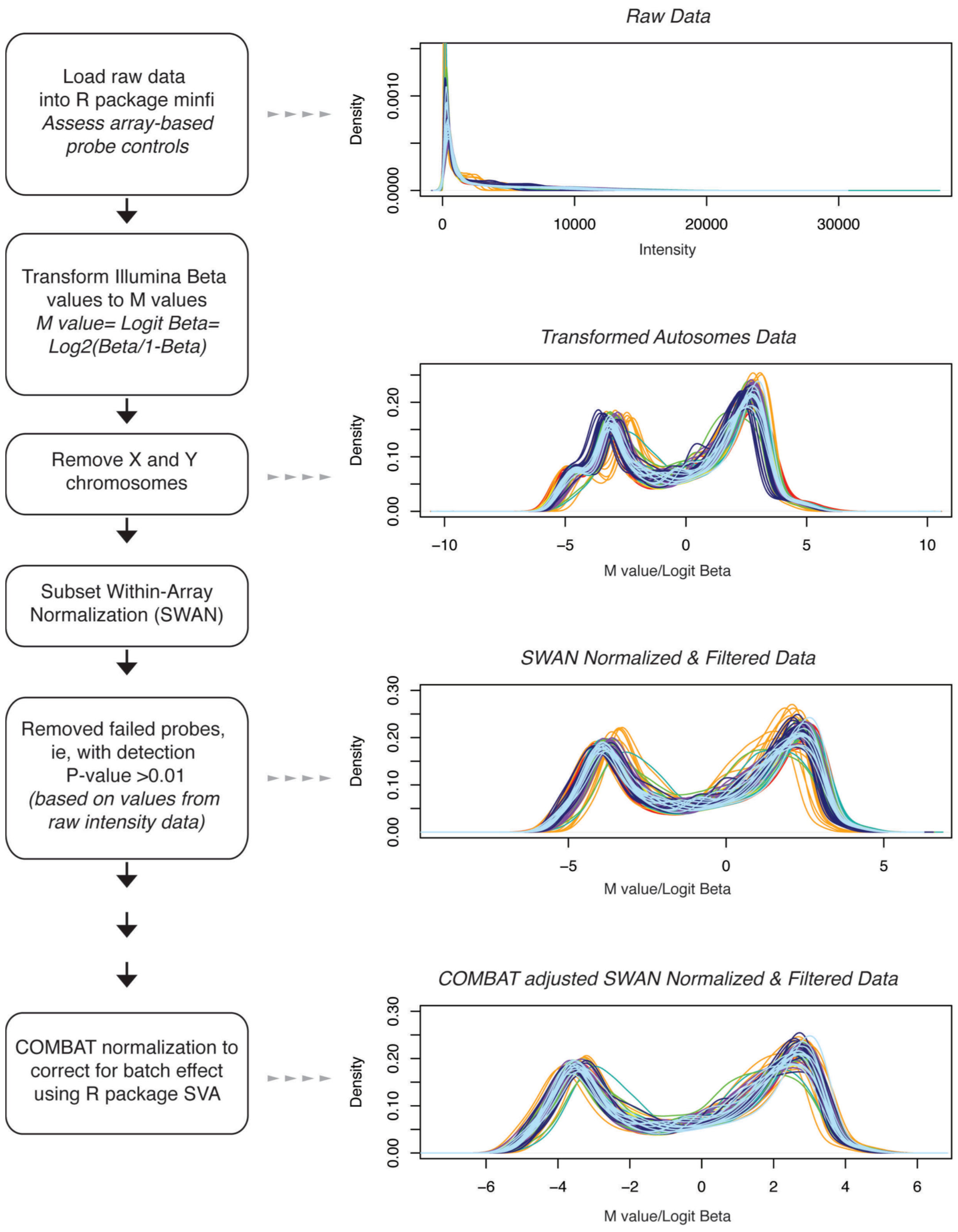

Supplemental Figure S3: Preprocessing of Illumina 450K data

The colors in the density plots correspond to different chips (microarrays), each run to contain 12 samples. The normalization procedures correct for both intra-array and inter-array differences.
