# Supplementary material for: Mosaic Epigenetic Dysregulation of Ectodermal Cells in Autism Spectrum Disorder
Source: PLoS Genet. 2014 May 29;10(5):e1004402. doi: 10.1371/journal.pgen.1004402 (PMC4038484; doi:10.1371/journal.pgen.1004402)
Supplement: Figure S4 — Associations between principal components and known covariates. Heat map of –log10 P-values for the association of each principal component with each known covariate demonstrates that variation due to technical artifact has been removed, while variation due to known biological covariates has been preserved for subsequent analysis. (PDF) [file pgen.1004402.s004.pdf]

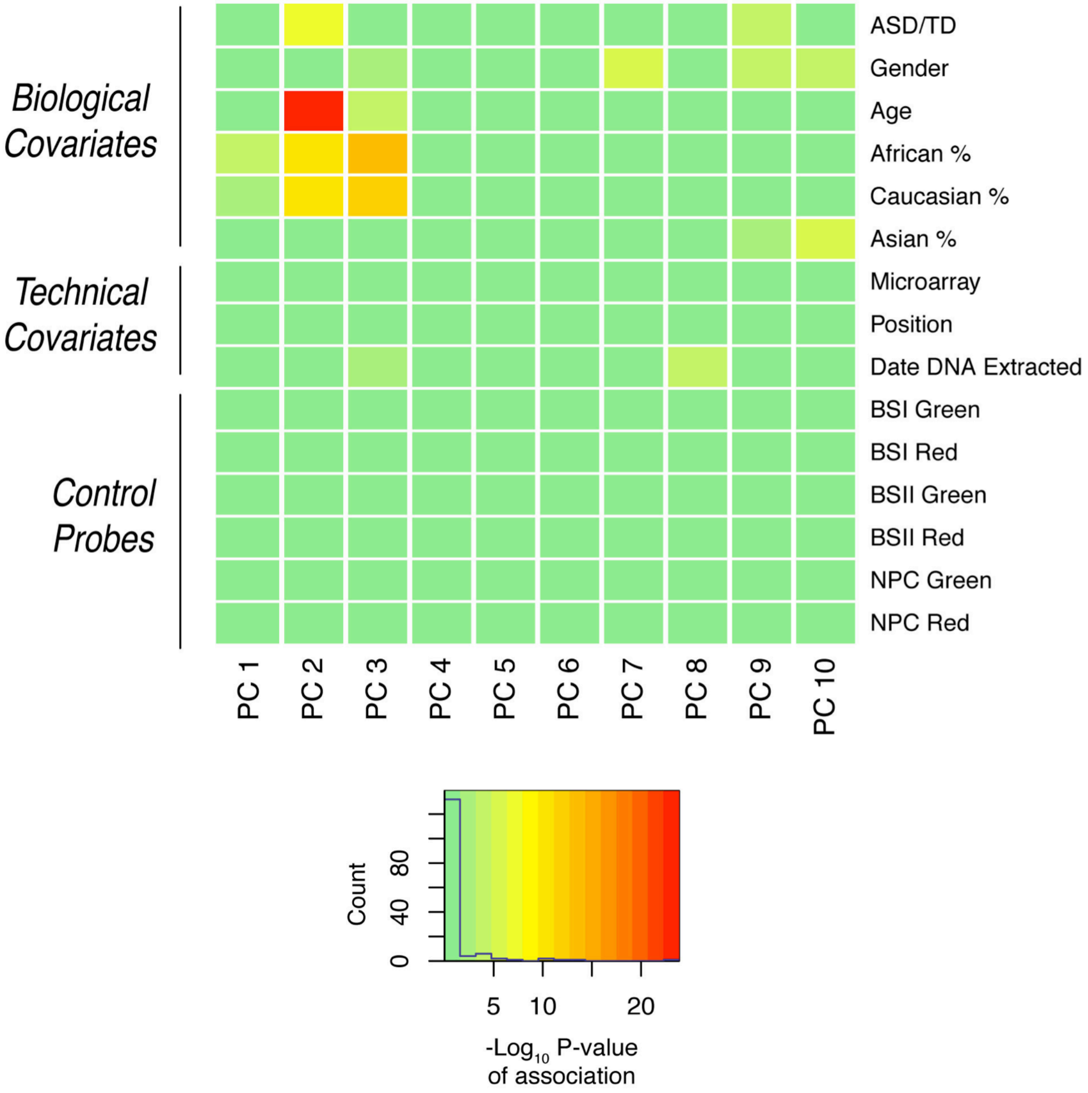

Supplemental Figure S4: Associations between principal components and known covariates

Heatmap of $-\log_{10}$ P-values for the association of each principal component with each known covariate demonstrates that variation due to technical artifact has been removed, while variation due to known biological covariates has been preserved for subsequent analysis.
