# Supplementary material for: Mosaic Epigenetic Dysregulation of Ectodermal Cells in Autism Spectrum Disorder
Source: PLoS Genet. 2014 May 29;10(5):e1004402. doi: 10.1371/journal.pgen.1004402 (PMC4038484; doi:10.1371/journal.pgen.1004402)
Supplement: Figure S5 — Stable DMRs defined by the bump-hunting algorithm. DNA methylation values are displayed along with -log10 p-values for each probe. Left column, from top to bottom, gene names: ADRA2C, GPC1, CREB5, HOOK2. Right column, top to bottom: PAX8, NOS1, MAPK8IP. (PDF) [file pgen.1004402.s005.pdf]

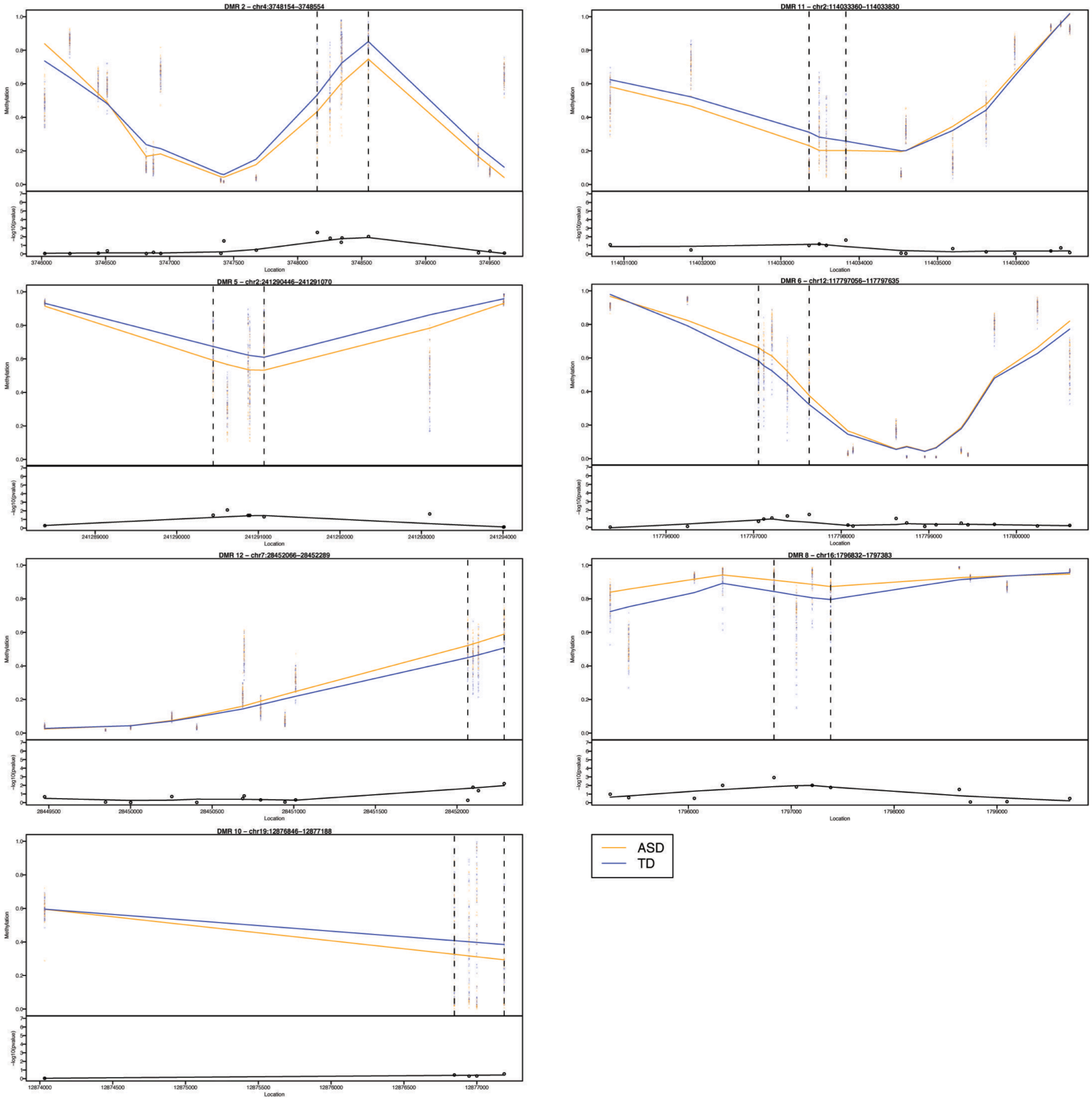

Supplemental Figure S5: Stable DMRs defined by the *bump-hunting* algorithm

DNA methylation values are displayed along with $-\log_{10}$ p-values for each probe.

Left column, from top to bottom, gene names: *ADRA2C, GPC1, CREB5, HOOK2*.

Right column, top to bottom: *PAX8, NOS1, MAPK8IP*.
