# Supplementary material for: Mosaic Epigenetic Dysregulation of Ectodermal Cells in Autism Spectrum Disorder
Source: PLoS Genet. 2014 May 29;10(5):e1004402. doi: 10.1371/journal.pgen.1004402 (PMC4038484; doi:10.1371/journal.pgen.1004402)
Supplement: Figure S6 — Results of massively-parallel bisulphite sequencing of the NOS1 and KCNQ5 DMRs. The p values were calculated using t tests combining all of the loci tested in the putative DMR and comparing between the ASD and TD groups. (PDF) [file pgen.1004402.s006.pdf]

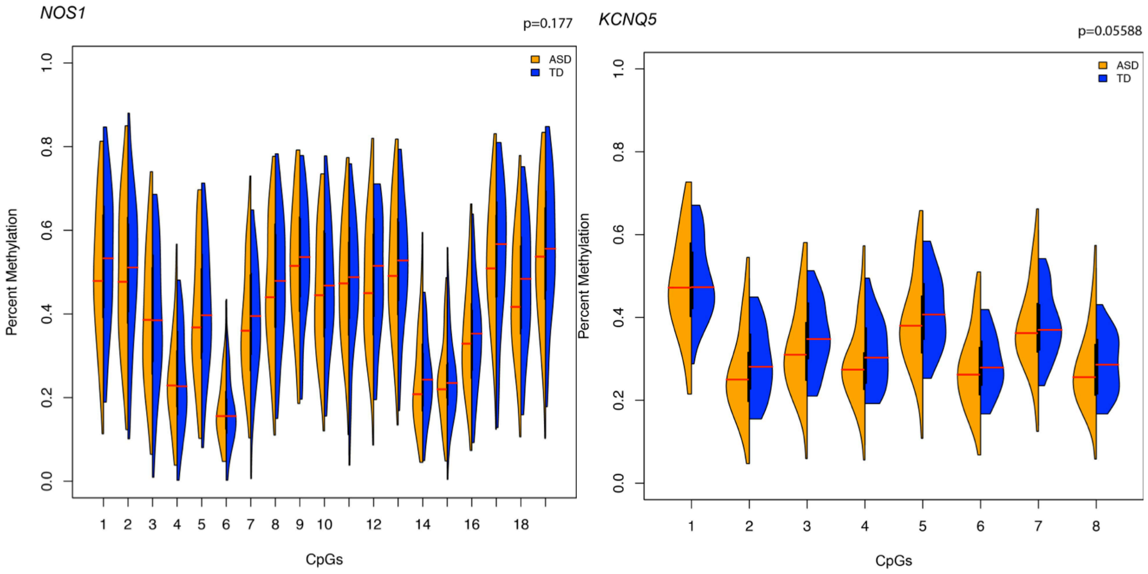

Supplemental Figure S6: Results of massively-parallel bisulphite sequencing of the *NOS1* and *KCNQ5* DMRs. The p values were calculated using t tests combining all of the loci tested in the putative DMR and comparing between the ASD and TD groups.
