# Supplementary material for: Mosaic Epigenetic Dysregulation of Ectodermal Cells in Autism Spectrum Disorder
Source: PLoS Genet. 2014 May 29;10(5):e1004402. doi: 10.1371/journal.pgen.1004402 (PMC4038484; doi:10.1371/journal.pgen.1004402)
Supplement: Figure S7 — Gene ontology analysis of genes associated with age-related DMRs. Panel A shows connectivity of the gene ontology categories significantly enriched for genes related to age-associated DMRs, plotted by corrected p-value in Panel B. (PDF) [file pgen.1004402.s007.pdf]

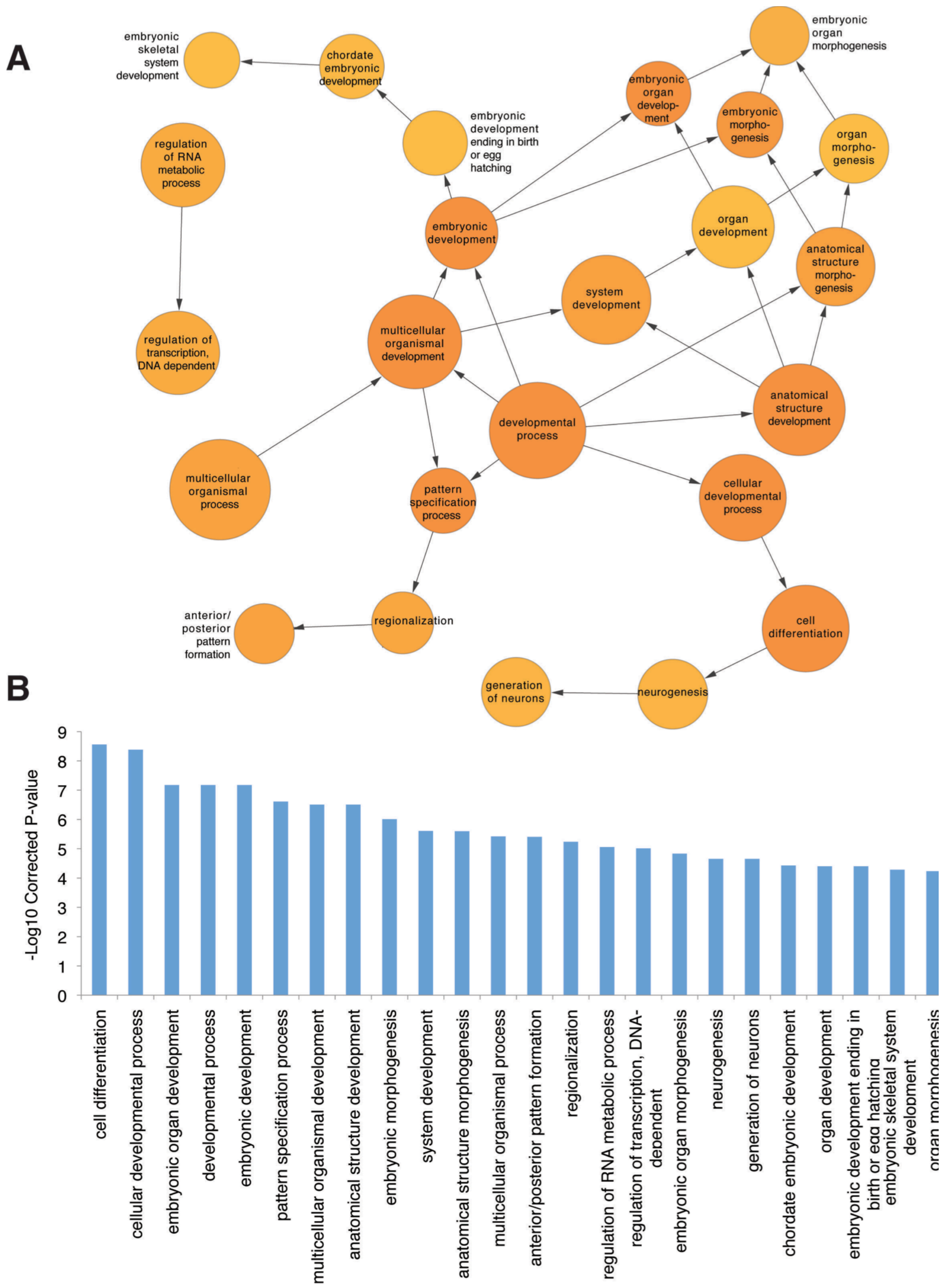

Supplemental Figure S7: Gene ontology analysis of genes associated with age-related DMRs.

Panel A shows connectivity of the gene ontology categories significantly enriched for genes related to age-associated DMRs, plotted by corrected p-value in **Panel B.**
