# Supplementary material for: Mosaic Epigenetic Dysregulation of Ectodermal Cells in Autism Spectrum Disorder
Source: PLoS Genet. 2014 May 29;10(5):e1004402. doi: 10.1371/journal.pgen.1004402 (PMC4038484; doi:10.1371/journal.pgen.1004402)
Supplement: Table S1 — Characteristics of study subjects. Age metrics reflect the mean age in years per group and the standard deviation, with the range included in parentheses. Maternal and paternal age refer to the age of the parents at the time of the subject's birth. All maternal ages are included, while information for paternal age was only available for ∼50% of subjects. Subject age refers to age when the sample was collected. Percent genetic ancestry based on subjects with quality filtered genotype data; 47 ASD and 46 TD genotypes were included. ASD: Autism Spectrum Disorder. TD: Typically Developing. CEU: Utah residents with Northern/Western European ancestry from the CEPH collection (European) YRI: Yoruba in Ibadan, Nigeria (African). (PDF) [file pgen.1004402.s009.pdf]

	ASD	TD
Ages		
Maternal age (mean, SD)	37.58, 2.93	38.1, 2.97
(range)	(35-48)	(35-48)
Paternal age	40.19, 6.27	40.72, 5.87
	(29-51)	(30-52)
Subject Age	6.84, 3.58	11.2, 7.31
	(2-17)	(1-28)
Gender		
Male (n)	39	22
Female	11	28
Genetic Ancestry		
% CEU (mean, SD)	63.38, 29.37	85.19, 16.38
% YRI	22.43, 30.74	6.61, 17.02
**Total**	**50**	**50**

Supplemental Table S1: Characteristics of study subjects.

Age metrics reflect the mean age in years per group and the standard deviation, with the range included in parentheses. Maternal and paternal age refer to the age of the parents at the time of the subject's birth. All maternal ages are included, while information for paternal age was only available for ~50% of subjects. Subject age refers to age when the sample was collected. Percent genetic ancestry based on subjects with quality filtered genotype data; 47 ASD and 46 TD genotypes were included.

ASD: Autism Spectrum Disorder.
TD: Typically Developing.
CEU: Utah residents with Northern/Western European ancestry from the CEPH collection (European)
YRI: Yoruba in Ibadan, Nigeria (African)
