# Supplementary material for: Mosaic Epigenetic Dysregulation of Ectodermal Cells in Autism Spectrum Disorder
Source: PLoS Genet. 2014 May 29;10(5):e1004402. doi: 10.1371/journal.pgen.1004402 (PMC4038484; doi:10.1371/journal.pgen.1004402)
Supplement: Table S2 — Experimental batches of microarrays. (PDF) [file pgen.1004402.s010.pdf]

	ASD	TD	Parents
Genotyping microarrays			
Batch 1	24	24<br>(including 2 siblings)	47
Batch 2	26	26<br>(including 5 siblings)	45
Methylation microarray			
**Batch 1**	**47**	**48**	**0**

Supplemental Table S2: Experimental batches of microarrays
