# Supplementary material for: Mosaic Epigenetic Dysregulation of Ectodermal Cells in Autism Spectrum Disorder
Source: PLoS Genet. 2014 May 29;10(5):e1004402. doi: 10.1371/journal.pgen.1004402 (PMC4038484; doi:10.1371/journal.pgen.1004402)
Supplement: Table S3 — HAPMIX local ancestry probability calculation. (PDF) [file pgen.1004402.s011.pdf]

Call	Probability Calculation (run number)
Homozygous YRI	Probability YRI (from run 1) **X**<br>Probability CEU/YRI (from run 2)
Homozygous CJ	Probability CEU/(CHB+JPT)(1) **X**<br>Probability (CHB+JPT)(2)
Homozygous CEU	Probability CEU/(CHB+JPT)(1) **X**<br>Probability CEU/YRI(2)
Heterozygous YRI/CJ	Probability heterozygous CEU/(CHB+JPT)& YRI(1) **X**<br>Probability heterozygous CEU/YRI&(CHB+JPT)(2)
Heterozygous YRI/CEU	Probability heterozygous CEU/(CHB+JPT)&YRI(1) **X**<br>Probability CEU/YRI(2)
Heterozygous CJ/CEU	Probability CEU/(CHB+JPT)(1) **X**<br>Probability heterozygous CEU/YRI&CJ(2)

Supplemental Table S3: *HAPMIX* local ancestry probability calculation

Abbreviations:

CHB: Han Chinese in Beijing, China.
JPT: Japanese in Tokyo, Japan
CJ: CHB/JPT (East Asian)
YRI: Yoruba in Ibadan, Nigeria (African)
CEU: Utah residents with Northern and Western European ancestry from the CEPH collection (European)
