# Supplementary material for: Mosaic Epigenetic Dysregulation of Ectodermal Cells in Autism Spectrum Disorder
Source: PLoS Genet. 2014 May 29;10(5):e1004402. doi: 10.1371/journal.pgen.1004402 (PMC4038484; doi:10.1371/journal.pgen.1004402)
Supplement: Table S4 — DMRs Associated with ASD from Bump-Hunting. Unstable DMRs are shaded in gray. (PDF) [file pgen.1004402.s012.pdf]

chromosome	start	end	value	area	pns	indexStart	indexEnd	nprobes	avg	max	area.raw	gene name	Illumina annotation
1	248,100,183	248,100,614	0.427	4.273	23,221	45,365	45,374	10	0.080	0.150	0.803	*OR2L13*	1st exon, 5'UTR, TSS200 and TSS 1500, CpG island and north shore
2	241,290,446	241,291,070	0.381	1.904	137,222	274,789	274,793	5	0.086	0.104	0.428	*GPC1*	north shelf
2	114,033,360	114,033,830	0.331	1.323	128,333	258,706	258,709	4	0.075	0.105	0.301	*PAX8*	gene body, CpG island and north shore
4	3,748,154	3,748,554	0.465	2.324	163,348	326,311	326,315	5	0.119	0.148	0.595	*ADRA2C*	CpG island and north shore
5	139,227,979	139,228,242	-0.158	0.790	180,529	358,423	358,427	5	-0.076	-0.127	0.378	*NRG2*	gene body, CpG island
5	16,508,920	16,509,123	0.373	1.491	174,420	347,241	347,244	4	0.078	0.095	0.312	*FAM134B*	TSS200, 5'UTR, 1st exon, body, enhancer
6	73,329,988	73,330,358	-0.209	1.253	192,434	389,727	389,732	6	-0.077	-0.088	0.461	*KCNQ5*	TSS1500, CpG island north shore
7	28,452,066	28,452,289	-0.202	0.806	202,381	410,259	410,262	4	-0.076	-0.083	0.302	*CREB5*	TSS200, 5'UTR, 1st exon, CpG island south shelf
10	135,341,870	135,342,620	-0.491	2.947	35,126	69,284	69,289	6	-0.078	-0.148	0.469	*CYP2E1*	gene body, CG island and south shore
10	135,342,936	135,343,280	-0.462	1.850	35,127	69,290	69,293	4	-0.078	-0.098	0.310	*CYP2E1*	gene body, south shore
12	117,797,056	117,797,635	-0.213	1.067	57,778	115,485	115,489	5	-0.086	-0.109	0.432	*NOS1*	5'UTR, north shore
16	53,407,678	53,407,808	0.174	0.696	87,732	174,199	174,202	4	0.089	0.115	0.357	*LOC643802*	CpG island, south shore
16	2,879,944	2,880,326	-0.265	1.060	83,603	166,459	166,462	4	-0.101	-0.125	0.402	*ZG16B*	TSS200, TSS1500, 1st exon
16	1,796,832	1,797,383	-0.515	2.060	83,090	165,290	165,293	4	-0.090	-0.139	0.361	*MAPK8IP3*	gene body, CpG island and south shore
19	12,876,846	12,877,188	0.401	1.603	111,948	225,403	225,406	4	0.079	0.086	0.315	*HOOK2*	gene body, CpG island, north shore and south shore

Supplemental Table S4: DMRs Associated with ASD from Bump-Hunting.

Unstable DMRs are shaded in gray.
