# Supplementary material for: Mosaic Epigenetic Dysregulation of Ectodermal Cells in Autism Spectrum Disorder
Source: PLoS Genet. 2014 May 29;10(5):e1004402. doi: 10.1371/journal.pgen.1004402 (PMC4038484; doi:10.1371/journal.pgen.1004402)
Supplement: Table S5 — Primers for bisulphite-converted DNA. (PDF) [file pgen.1004402.s013.pdf]

Locus	Forward Primer	Reverse Primer
chr12:117797179-117797517	GGGGAAAAAATTTATGTTTTAGAGAG	AAAATTCTTCCCTCTACTCCCATAAC
chr6:73330181-73330482	GGTTTTTGTTGGTGATTAGGAGTAG	AAAAACAAAACTAAACTTCCACCAC
chr1:248100298-248100643	TTTTATTGTTTTTGGGGTTAATTAT	CACCAATATATAAAACAAAACCCTTC
chr5:16508806-16509201	TATTTTAATGTTGAATATTAGGAGGAAAA	AACCACTCCACCCTTAAATAAATAC

Supplemental Table S5: Primers for bisulphite-converted DNA
