# Supplementary material for: Mosaic Epigenetic Dysregulation of Ectodermal Cells in Autism Spectrum Disorder
Source: PLoS Genet. 2014 May 29;10(5):e1004402. doi: 10.1371/journal.pgen.1004402 (PMC4038484; doi:10.1371/journal.pgen.1004402)
Supplement: Table S6 — Age-associated candidate DMRs. The table shows the results of the dmrFind algorithm. DMR positions are shown in the chr/start/end co-ordinates, with probe indices and numbers represented by indexStart, indexEnd and nprobes, and area_raw the significance calculation following permutation analysis, allowing ranking of these DMRs by significance, as shown. (PDF) [file pgen.1004402.s014.pdf]

Supplemental Table S6: Age-associated candidate DMRs.

chr	start	end	value	area	pns	indexStart	indexEnd	nprobes	avg	max	area.raw
**chr2**	176,986,460	176,987,465	0.029	0.318	132,157	265,348	265,358	11	0.732	0.855	8.054
**chr1**	231,155,632	231,156,204	-0.039	0.467	21,845	42,875	42,886	12	-0.609	-0.707	7.312
**chr6**	32,118,295	32,118,457	0.026	0.290	189,023	380,814	380,824	11	0.659	0.753	7.250
**chr19**	58,220,295	58,220,837	0.027	0.271	119,212	241,574	241,583	10	0.697	0.817	6.965
**chr14**	24,641,021	24,641,852	0.048	0.529	67,268	134,784	134,794	11	0.606	0.635	6.670
**chr8**	23,563,970	23,564,717	0.027	0.246	215,946	436,377	436,385	9	0.729	0.859	6.557
**chr1**	110,610,899	110,612,044	0.033	0.267	12,843	25,512	25,519	8	0.799	0.850	6.392
**chr19**	9,473,565	9,473,781	0.032	0.285	111,176	223,482	223,490	9	0.691	0.797	6.217
**chr16**	66,612,955	66,613,334	0.037	0.334	88,644	175,913	175,921	9	0.663	0.794	5.970
**chr10**	22,634,038	22,634,226	0.046	0.320	25,480	49,991	49,997	7	0.832	0.864	5.825
**chr13**	79,169,714	79,170,303	0.027	0.217	63,849	127,718	127,725	8	0.718	0.788	5.745
**chr6**	32,078,398	32,078,624	-0.028	0.225	189,003	380,630	380,637	8	-0.691	-0.752	5.525
**chr2**	177,029,459	177,030,171	0.027	0.192	132,199	265,502	265,508	7	0.775	0.798	5.425
**chr14**	29,234,890	29,235,196	0.029	0.229	67,445	135,202	135,209	8	0.666	0.827	5.332
**chr5**	87,441,081	87,441,969	0.031	0.249	177,623	353,048	353,055	8	0.659	0.812	5.272
**chr3**	147,126,638	147,127,097	0.035	0.244	158,563	316,905	316,911	7	0.752	0.852	5.264
**chr7**	27,205,200	27,205,658	0.025	0.223	202,198	409,826	409,834	9	0.583	0.696	5.251
**chr4**	155,661,691	155,662,795	0.029	0.259	170,611	339,758	339,766	9	0.556	0.630	5.004
**chr11**	86,383,182	86,383,430	0.041	0.286	44,708	90,004	90,010	7	0.707	0.738	4.950
**chr22**	24,890,690	24,890,833	0.043	0.301	145,700	292,479	292,485	7	0.678	0.766	4.744

**chr8**	143,858,414	143,858,636	0.036	0.255	223,217	449,399	449,405	7	0.669	0.736	4.680
**chr7**	99,775,422	99,775,558	0.034	0.240	207,428	419,896	419,902	7	0.666	0.745	4.661
**chr14**	57,275,967	57,276,789	0.034	0.204	68,821	137,800	137,805	6	0.773	0.871	4.638
**chr7**	27,225,058	27,225,143	0.029	0.172	202,219	409,908	409,913	6	0.772	0.848	4.631
**chr17**	7,832,680	7,833,237	0.028	0.255	94,610	188,539	188,547	9	0.511	0.575	4.602
**chr1**	248,020,436	248,020,812	0.043	0.258	23,207	45,341	45,346	6	0.749	0.833	4.492
**chr7**	8,481,994	8,482,614	0.037	0.225	201,229	407,774	407,779	6	0.747	0.836	4.481
**chr16**	87,864,324	87,865,062	-0.050	0.300	91,530	181,608	181,613	6	-0.721	-0.750	4.325
**chr1**	92,952,440	92,952,533	0.034	0.202	11,997	23,805	23,810	6	0.715	0.761	4.290
**chr2**	74,875,227	74,875,387	0.042	0.254	125,794	254,086	254,091	6	0.713	0.788	4.276
**chr8**	24,772,137	24,772,350	0.039	0.232	216,010	436,484	436,489	6	0.706	0.777	4.233
**chr14**	54,413,218	54,413,931	0.052	0.310	68,535	137,302	137,307	6	0.704	0.806	4.221
**chr10**	103,603,292	103,603,869	0.029	0.205	31,479	61,239	61,245	7	0.602	0.702	4.215
**chr1**	91,301,204	91,301,962	0.029	0.172	11,874	23,499	23,504	6	0.695	0.766	4.170
**chr3**	120,626,881	120,627,088	0.031	0.155	156,324	312,596	312,600	5	0.830	0.849	4.148
**chr2**	10,182,878	10,183,227	0.043	0.258	120,711	244,696	244,701	6	0.690	0.742	4.140
**chr12**	106,533,667	106,533,903	0.040	0.241	56,479	112,724	112,729	6	0.687	0.745	4.119
**chr11**	2,292,751	2,292,914	0.036	0.253	36,185	72,072	72,078	7	0.588	0.751	4.116
**chr6**	100,903,561	100,903,909	0.025	0.148	193,518	391,945	391,950	6	0.686	0.807	4.113
**chr6**	85,474,028	85,474,209	0.030	0.178	192,887	390,662	390,667	6	0.680	0.859	4.083
**chr11**	94,278,324	94,278,603	0.032	0.259	45,079	90,675	90,682	8	0.508	0.574	4.061
**chr5**	87,980,882	87,981,253	0.026	0.158	177,666	353,160	353,165	6	0.670	0.802	4.020
**chr2**	220,299,643	220,299,900	0.032	0.190	134,869	270,356	270,361	6	0.667	0.762	4.003

**chr22**	30,476,089	30,476,285	0.033	0.230	146,109	293,345	293,351	7	0.565	0.685	3.953
**chr5**	78,407,552	78,407,683	0.029	0.171	177,238	352,385	352,390	6	0.657	0.800	3.943
**chr6**	29,943,268	29,943,480	0.038	0.308	188,133	374,577	374,584	8	0.489	0.583	3.911
**chr1**	79,472,282	79,472,452	0.030	0.152	11,278	22,419	22,423	5	0.779	0.847	3.895
**chr18**	44,526,430	44,527,026	0.031	0.246	106,733	214,413	214,420	8	0.485	0.657	3.883
**chr13**	100,624,279	100,624,373	0.026	0.128	64,614	129,099	129,103	5	0.776	0.832	3.880
**chr5**	43,017,982	43,018,629	0.045	0.227	175,429	349,043	349,047	5	0.760	0.817	3.802
**chr4**	85,414,016	85,414,486	0.027	0.134	167,599	334,300	334,304	5	0.754	0.792	3.772
**chr22**	24,181,191	24,181,270	0.034	0.169	145,604	292,299	292,303	5	0.754	0.818	3.771
**chr5**	172,672,390	172,672,817	0.027	0.136	183,336	363,882	363,886	5	0.749	0.773	3.744
**chr1**	151,810,586	151,810,904	0.031	0.187	14,673	29,289	29,294	6	0.621	0.691	3.729
**chr3**	147,127,579	147,128,157	0.034	0.169	158,564	316,915	316,919	5	0.744	0.861	3.722
**chr7**	100,463,416	100,464,145	-0.059	0.353	207,617	420,346	420,351	6	-0.615	-0.639	3.692
**chr13**	79,170,627	79,171,230	0.028	0.139	63,849	127,727	127,731	5	0.737	0.838	3.683
**chr4**	85,402,870	85,403,409	0.027	0.137	167,592	334,286	334,290	5	0.732	0.761	3.658
**chr16**	86,547,203	86,547,544	0.027	0.137	91,139	180,813	180,817	5	0.731	0.767	3.654
**chr11**	86,085,623	86,086,005	0.026	0.158	44,685	89,972	89,977	6	0.609	0.731	3.652
**chr7**	19,146,032	19,146,555	0.025	0.123	201,554	408,365	408,369	5	0.730	0.799	3.650
**chr2**	223,164,831	223,164,925	0.027	0.134	135,087	270,748	270,752	5	0.727	0.805	3.637
**chr5**	176,827,082	176,827,793	0.040	0.237	183,904	364,948	364,953	6	0.599	0.723	3.597
**chr3**	147,125,712	147,125,782	0.031	0.157	158,563	316,888	316,892	5	0.717	0.828	3.586
**chr6**	85,482,570	85,483,055	0.032	0.159	192,892	390,680	390,684	5	0.715	0.793	3.573
**chr6**	11,044,877	11,044,974	0.067	0.269	186,057	369,233	369,236	4	0.892	0.905	3.568

**chr2**	176,948,693	176,948,759	0.029	0.147	132,115	265,209	265,213	5	0.706	0.784	3.528
**chr1**	91,190,366	91,190,891	0.028	0.139	11,865	23,457	23,461	5	0.705	0.795	3.527
**chr17**	46,685,292	46,685,448	0.028	0.141	99,899	199,582	199,586	5	0.701	0.744	3.505
**chr16**	1,593,152	1,593,766	-0.034	0.202	83,002	165,085	165,090	6	-0.583	-0.705	3.499
**chr16**	68,482,591	68,482,821	0.030	0.148	89,083	176,948	176,952	5	0.697	0.806	3.483
**chr11**	2,891,065	2,891,118	0.031	0.154	36,402	72,730	72,734	5	0.691	0.737	3.456
**chr16**	51,187,388	51,187,807	0.030	0.148	87,625	174,051	174,055	5	0.686	0.801	3.429
**chr10**	93,805,441	93,805,870	0.028	0.139	30,293	58,645	58,649	5	0.669	0.772	3.344
**chr17**	78,999,347	78,999,895	-0.032	0.162	104,247	208,702	208,706	5	-0.668	-0.739	3.342
**chr14**	100,069,535	100,069,840	0.055	0.222	72,269	144,115	144,118	4	0.827	0.851	3.307
**chr22**	40,417,285	40,417,869	-0.043	0.214	147,204	295,668	295,672	5	-0.653	-0.769	3.264
**chr12**	113,916,473	113,916,664	0.030	0.122	57,370	114,617	114,620	4	0.811	0.828	3.242
**chr8**	97,157,756	97,158,052	0.026	0.128	220,041	443,885	443,889	5	0.648	0.741	3.240
**chr1**	164,545,553	164,546,143	0.035	0.174	16,595	33,260	33,264	5	0.646	0.810	3.229
**chr3**	14,614,882	14,615,579	-0.032	0.161	149,961	301,002	301,006	5	-0.641	-0.692	3.203
**chr6**	101,846,779	101,846,872	0.026	0.130	193,554	392,047	392,051	5	0.638	0.692	3.191
**chr2**	176,964,506	176,964,720	0.030	0.118	132,130	265,271	265,274	4	0.798	0.830	3.190
**chr6**	73,329,988	73,330,358	0.036	0.217	192,434	389,727	389,732	6	0.529	0.610	3.176
**chr1**	119,535,693	119,535,986	0.038	0.153	13,653	27,185	27,188	4	0.794	0.872	3.175
**chr11**	123,066,529	123,067,275	0.030	0.181	47,241	94,847	94,852	6	0.529	0.683	3.174
**chr1**	228,400,217	228,400,693	0.034	0.136	21,459	42,125	42,128	4	0.788	0.822	3.153
**chr8**	56,015,399	56,015,785	0.026	0.105	218,073	440,284	440,287	4	0.784	0.808	3.135
**chr5**	170,288,742	170,289,070	0.034	0.171	183,006	363,218	363,222	5	0.625	0.864	3.127

**chr8**	25,898,191	25,898,539	0.031	0.126	216,106	436,652	436,655	4	0.778	0.803	3.111
**chr5**	126,626,348	126,626,364	0.025	0.099	179,378	356,130	356,133	4	0.777	0.826	3.106
**chr11**	134,147,143	134,147,634	0.034	0.136	48,559	97,232	97,235	4	0.773	0.855	3.091
**chr19**	17,958,339	17,958,736	0.032	0.159	113,027	227,806	227,810	5	0.618	0.744	3.091
**chr19**	57,182,844	57,183,268	0.031	0.124	119,037	241,073	241,076	4	0.768	0.814	3.070
**chr10**	28,035,631	28,035,894	0.038	0.267	25,811	50,667	50,673	7	0.439	0.487	3.070
**chr13**	79,177,877	79,177,925	0.027	0.107	63,854	127,748	127,751	4	0.759	0.790	3.037
**chr7**	101,005,910	101,006,089	0.031	0.215	207,774	420,718	420,724	7	0.433	0.521	3.032
**chr11**	64,146,487	64,146,822	-0.034	0.172	41,583	83,185	83,189	5	-0.606	-0.642	3.030
**chr7**	153,584,416	153,584,609	0.034	0.136	211,900	428,260	428,263	4	0.757	0.787	3.029
**chr1**	75,595,919	75,596,336	0.029	0.118	11,085	22,047	22,050	4	0.757	0.834	3.029
**chr8**	11,555,178	11,555,548	0.029	0.114	214,859	434,416	434,419	4	0.756	0.799	3.025
**chr12**	54,071,090	54,071,194	0.033	0.163	53,207	106,359	106,363	5	0.605	0.704	3.024
**chr1**	39,957,387	39,957,400	0.026	0.105	8,008	16,050	16,053	4	0.752	0.768	3.009
**chr5**	87,968,528	87,968,749	0.029	0.115	177,652	353,113	353,116	4	0.752	0.830	3.008
**chr10**	88,149,210	88,149,632	0.040	0.159	29,828	57,728	57,731	4	0.752	0.813	3.007
**chr1**	18,959,268	18,959,625	0.025	0.101	4,757	9,510	9,513	4	0.742	0.756	2.966
**chr2**	239,139,911	239,140,190	0.032	0.195	136,760	273,781	273,786	6	0.494	0.662	2.962
**chr16**	29,625,216	29,625,259	0.036	0.145	86,317	171,434	171,437	4	0.739	0.794	2.958
**chr3**	147,111,120	147,111,308	0.024	0.098	158,547	316,846	316,849	4	0.737	0.803	2.947
**chr11**	35,441,558	35,441,900	0.029	0.144	39,114	78,143	78,147	5	0.588	0.723	2.942
**chr2**	200,329,654	200,329,680	0.028	0.113	133,200	267,328	267,331	4	0.735	0.837	2.941
**chr5**	132,083,532	132,084,068	0.032	0.127	179,674	356,716	356,719	4	0.734	0.835	2.938

**chr8**	25,905,478	25,905,811	0.028	0.112	216,114	436,684	436,687	4	0.732	0.775	2.928
**chr7**	27,245,018	27,245,747	0.026	0.103	202,239	409,982	409,985	4	0.724	0.765	2.896
**chr1**	154,475,068	154,475,269	0.032	0.127	15,125	30,199	30,202	4	0.723	0.793	2.891
**chr18**	70,534,298	70,535,005	0.027	0.135	107,430	215,904	215,908	5	0.578	0.744	2.888
**chr5**	87,979,441	87,979,871	0.026	0.105	177,664	353,155	353,158	4	0.721	0.816	2.884
**chr14**	95,239,381	95,239,751	0.026	0.131	71,852	143,386	143,390	5	0.576	0.861	2.882
**chr11**	66,102,055	66,102,352	-0.029	0.144	42,230	84,801	84,805	5	-0.572	-0.642	2.861
**chr6**	100,054,585	100,054,817	0.026	0.104	193,460	391,824	391,827	4	0.714	0.855	2.855
**chr19**	2,046,085	2,046,350	-0.050	0.199	109,187	219,324	219,327	4	-0.711	-0.744	2.846
**chr6**	100,442,105	100,442,151	0.037	0.150	193,479	391,871	391,874	4	0.710	0.720	2.841
**chr18**	49,868,378	49,868,552	0.027	0.108	106,986	214,941	214,944	4	0.708	0.758	2.832
**chr3**	62,354,991	62,355,443	0.030	0.118	153,866	308,533	308,536	4	0.708	0.780	2.832
**chr4**	174,430,487	174,431,058	0.024	0.096	171,282	340,983	340,986	4	0.706	0.731	2.825
**chr3**	187,387,555	187,387,734	0.031	0.124	160,929	321,238	321,241	4	0.706	0.841	2.823
**chr10**	22,634,578	22,635,028	0.025	0.102	25,480	50,000	50,003	4	0.699	0.782	2.796
**chr6**	134,213,992	134,214,307	0.027	0.109	195,293	395,191	395,194	4	0.699	0.753	2.794
**chr17**	40,715,222	40,715,281	-0.026	0.132	98,728	196,894	196,898	5	-0.558	-0.644	2.788
**chr4**	52,942,997	52,943,247	0.042	0.169	166,053	331,543	331,546	4	0.696	0.757	2.784
**chr2**	223,161,771	223,162,128	0.032	0.127	135,085	270,727	270,730	4	0.695	0.792	2.780
**chr11**	111,385,450	111,385,659	0.027	0.109	45,879	92,155	92,158	4	0.695	0.805	2.779
**chr15**	60,288,082	60,288,404	0.026	0.103	77,285	154,102	154,105	4	0.694	0.759	2.777
**chr21**	36,041,605	36,041,699	0.033	0.134	143,100	287,541	287,544	4	0.694	0.716	2.775
**chr6**	156,718,177	156,718,546	0.039	0.193	196,828	397,938	397,942	5	0.554	0.626	2.770

**chr8**	98,290,310	98,290,372	0.034	0.137	220,135	444,065	444,068	4	0.692	0.826	2.767
**chr15**	58,357,922	58,357,989	0.028	0.110	77,129	153,822	153,825	4	0.691	0.723	2.764
**chr4**	48,486,087	48,486,472	0.026	0.106	165,951	331,335	331,338	4	0.687	0.752	2.748
**chr17**	36,719,518	36,719,937	0.034	0.172	97,771	194,856	194,860	5	0.549	0.726	2.744
**chr1**	19,665,070	19,665,240	0.040	0.161	4,926	9,817	9,820	4	0.686	0.731	2.744
**chr10**	126,136,228	126,136,709	0.059	0.237	33,326	64,892	64,895	4	0.683	0.755	2.733
**chr10**	50,604,330	50,604,569	0.024	0.097	27,342	53,347	53,350	4	0.680	0.779	2.721
**chr16**	2,563,274	2,563,560	0.024	0.097	83,480	166,190	166,193	4	0.679	0.803	2.717
**chr19**	10,397,612	10,397,780	0.031	0.125	111,331	223,899	223,902	4	0.678	0.766	2.713
**chr13**	37,004,721	37,004,812	0.025	0.127	61,968	124,283	124,287	5	0.542	0.570	2.711
**chr17**	61,778,366	61,778,813	0.025	0.100	101,437	202,761	202,764	4	0.675	0.730	2.701
**chr17**	58,216,297	58,216,651	0.030	0.119	101,114	202,101	202,104	4	0.672	0.755	2.688
**chr20**	55,964,998	55,965,497	0.035	0.138	141,209	283,686	283,689	4	0.671	0.781	2.682
**chr2**	157,176,971	157,177,345	0.035	0.141	130,820	262,911	262,914	4	0.671	0.825	2.682
**chr8**	143,545,478	143,545,949	0.034	0.170	223,102	449,175	449,179	5	0.536	0.619	2.680
**chr8**	1,273,592	1,273,856	0.028	0.139	213,656	432,090	432,094	5	0.535	0.646	2.674
**chr12**	66,627,900	66,628,232	-0.025	0.099	54,519	109,239	109,242	4	-0.667	-0.715	2.668
**chr14**	29,243,404	29,243,690	0.028	0.114	67,454	135,237	135,240	4	0.666	0.790	2.665
**chr1**	41,119,634	41,119,988	0.030	0.121	8,190	16,458	16,461	4	0.660	0.741	2.638
**chr6**	30,653,512	30,653,659	-0.028	0.112	188,431	376,470	376,473	4	-0.659	-0.679	2.635
**chr4**	151,500,631	151,501,298	0.037	0.148	170,283	339,171	339,174	4	0.659	0.774	2.634
**chr16**	85,932,591	85,932,853	0.031	0.123	90,989	180,519	180,522	4	0.658	0.752	2.632
**chr2**	177,053,274	177,053,292	0.028	0.113	132,211	265,539	265,542	4	0.658	0.724	2.631

**chr13**	24,844,846	24,844,938	0.028	0.168	60,981	122,394	122,399	6	0.438	0.541	2.631
**chr12**	56,414,442	56,414,533	0.025	0.099	53,637	107,388	107,391	4	0.654	0.701	2.616
**chr11**	46,383,031	46,383,209	0.028	0.111	39,798	79,438	79,441	4	0.653	0.724	2.611
**chr19**	18,260,330	18,260,515	-0.033	0.131	113,106	227,997	228,000	4	-0.650	-0.685	2.599
**chr12**	126,675,667	126,676,048	0.026	0.103	59,051	118,197	118,200	4	0.650	0.770	2.598
**chr6**	110,736,772	110,737,053	-0.033	0.163	194,095	393,045	393,049	5	-0.517	-0.584	2.586
**chr15**	67,356,310	67,356,942	0.035	0.174	78,156	155,651	155,655	5	0.516	0.559	2.579
**chr8**	61,777,711	61,778,137	-0.036	0.146	218,407	440,878	440,881	4	-0.643	-0.724	2.572
**chr1**	6,515,580	6,515,748	0.036	0.145	2,387	5,290	5,293	4	0.643	0.703	2.570
**chr11**	17,803,160	17,803,421	-0.036	0.145	38,057	76,022	76,025	4	-0.642	-0.684	2.567
**chr17**	46,618,919	46,619,555	0.026	0.104	99,835	199,378	199,381	4	0.641	0.826	2.565
**chr10**	99,734,416	99,734,912	0.034	0.169	30,914	59,888	59,892	5	0.513	0.616	2.564
**chr4**	175,132,842	175,133,151	0.024	0.096	171,327	341,091	341,094	4	0.640	0.744	2.561
**chr1**	200,009,830	200,010,283	0.024	0.097	18,525	36,715	36,718	4	0.638	0.685	2.551
**chr5**	54,518,745	54,519,159	0.028	0.113	175,795	349,774	349,777	4	0.637	0.754	2.549
**chr6**	30,070,059	30,070,403	0.038	0.339	188,216	375,015	375,023	9	0.283	0.533	2.543
**chr20**	33,762,474	33,762,943	0.028	0.111	139,752	280,319	280,322	4	0.635	0.727	2.540
**chr7**	94,284,865	94,284,900	0.030	0.121	206,839	418,369	418,372	4	0.634	0.835	2.535
**chr2**	175,208,588	175,208,761	0.026	0.103	131,985	264,936	264,939	4	0.631	0.743	2.526
**chr14**	94,392,718	94,392,932	-0.030	0.121	71,715	143,118	143,121	4	-0.630	-0.671	2.519
**chr12**	50,426,531	50,427,095	0.032	0.127	52,527	104,971	104,974	4	0.628	0.698	2.512
**chr1**	17,085,860	17,086,071	0.030	0.121	4,431	8,941	8,944	4	0.627	0.684	2.508
**chr17**	27,038,861	27,039,058	0.028	0.110	96,631	192,556	192,559	4	0.627	0.717	2.506

**chr12**	11,653,278	11,653,827	0.024	0.098	50,245	100,630	100,633	4	0.626	0.842	2.505
**chr21**	36,399,146	36,399,540	0.041	0.166	143,128	287,597	287,600	4	0.625	0.684	2.498
**chr7**	142,494,148	142,494,244	0.028	0.113	210,754	426,012	426,015	4	0.622	0.724	2.490
**chr2**	74,663,416	74,663,698	0.026	0.104	125,719	253,871	253,874	4	0.622	0.725	2.487
**chr1**	91,300,288	91,300,446	0.025	0.102	11,873	23,494	23,497	4	0.622	0.653	2.486
**chr17**	42,733,527	42,733,600	0.033	0.130	99,202	198,076	198,079	4	0.611	0.835	2.443
**chr10**	110,225,900	110,226,387	0.025	0.099	31,992	62,298	62,301	4	0.611	0.627	2.443
**chr1**	3,473,665	3,474,376	-0.028	0.111	1,736	3,933	3,936	4	-0.610	-0.781	2.440
**chr13**	28,545,214	28,545,566	0.025	0.098	61,363	123,182	123,185	4	0.607	0.697	2.429
**chr3**	194,408,516	194,408,901	0.036	0.143	161,474	322,164	322,167	4	0.607	0.695	2.427
**chr1**	33,231,272	33,231,382	0.035	0.140	7,123	14,255	14,258	4	0.603	0.623	2.412
**chr17**	1,881,005	1,881,333	0.036	0.145	93,375	185,564	185,567	4	0.601	0.611	2.404
**chr1**	115,881,130	115,881,259	0.029	0.114	13,375	26,633	26,636	4	0.601	0.698	2.403
**chr4**	109,093,158	109,093,243	0.025	0.101	168,519	336,010	336,013	4	0.595	0.663	2.380
**chr2**	186,603,398	186,603,639	0.030	0.120	132,626	266,293	266,296	4	0.592	0.691	2.369
**chr2**	220,196,530	220,196,755	0.031	0.125	134,841	270,288	270,291	4	0.592	0.800	2.368
**chr7**	101,512,529	101,513,100	0.025	0.099	207,835	420,826	420,829	4	0.592	0.650	2.367
**chr13**	112,709,256	112,709,550	0.024	0.097	65,560	130,940	130,943	4	0.590	0.803	2.361
**chr10**	94,451,351	94,451,736	0.026	0.105	30,344	58,759	58,762	4	0.589	0.644	2.358
**chr6**	29,521,781	29,521,803	0.030	0.121	187,956	373,759	373,762	4	0.588	0.632	2.352
**chr17**	4,648,566	4,648,949	0.034	0.135	93,932	186,796	186,799	4	0.586	0.755	2.346
**chr19**	11,529,947	11,530,065	0.029	0.145	111,665	224,649	224,653	5	0.468	0.552	2.342
**chr8**	10,261,972	10,262,221	0.029	0.117	214,638	433,984	433,987	4	0.585	0.697	2.338

**chr11**	125,036,088	125,036,420	0.030	0.119	47,523	95,377	95,380	4	0.584	0.668	2.334
**chr1**	4,770,676	4,771,201	0.034	0.136	2,015	4,574	4,577	4	0.583	0.707	2.333
**chr12**	58,003,774	58,003,965	0.025	0.127	54,003	108,274	108,278	5	0.466	0.629	2.331
**chr11**	9,025,730	9,026,308	0.035	0.140	37,246	74,531	74,534	4	0.580	0.758	2.322
**chr11**	334,298	334,833	0.035	0.138	35,235	69,532	69,535	4	0.576	0.582	2.305
**chr17**	7,350,001	7,350,413	0.026	0.103	94,440	188,076	188,079	4	0.574	0.627	2.297
**chr1**	47,882,686	47,883,234	0.025	0.102	9,213	18,622	18,625	4	0.574	0.790	2.295
**chr10**	8,097,331	8,097,689	0.032	0.128	24,556	48,236	48,239	4	0.573	0.672	2.293
**chr4**	41,646,293	41,646,672	0.026	0.105	165,708	330,788	330,791	4	0.572	0.657	2.290
**chr13**	112,717,207	112,717,707	0.031	0.126	65,571	130,968	130,971	4	0.571	0.630	2.284
**chr1**	228,246,632	228,247,135	0.027	0.108	21,403	41,970	41,973	4	0.570	0.623	2.282
**chr22**	17,083,412	17,083,727	0.025	0.101	144,702	290,507	290,510	4	0.570	0.658	2.280
**chr8**	145,106,246	145,106,582	0.039	0.155	223,787	450,753	450,756	4	0.568	0.584	2.272
**chr6**	125,855,124	125,855,421	0.027	0.110	194,820	394,326	394,329	4	0.565	0.583	2.261
**chr6**	170,597,326	170,597,588	-0.036	0.145	198,758	401,914	401,917	4	-0.562	-0.663	2.248
**chr5**	172,110,211	172,110,579	0.028	0.113	183,213	363,608	363,611	4	0.559	0.695	2.237
**chr11**	1,483,731	1,483,973	0.026	0.103	35,852	71,128	71,131	4	0.559	0.663	2.236
**chr12**	113,913,695	113,914,222	0.028	0.112	57,369	114,609	114,612	4	0.557	0.748	2.229
**chr1**	236,557,182	236,557,682	-0.032	0.127	22,345	43,778	43,781	4	-0.557	-0.739	2.229
**chr3**	100,712,058	100,712,345	-0.024	0.097	155,320	310,850	310,853	4	-0.555	-0.560	2.221
**chr15**	45,996,521	45,996,787	0.028	0.112	76,437	152,529	152,532	4	0.553	0.665	2.213
**chr15**	37,387,304	37,387,577	0.032	0.160	75,453	150,422	150,426	5	0.443	0.507	2.213
**chr22**	19,748,777	19,749,188	0.032	0.126	145,030	291,203	291,206	4	0.552	0.710	2.208

**chr19**	3,097,565	3,097,728	-0.028	0.111	109,601	220,187	220,190	4	-0.549	-0.559	2.197
**chr16**	66,400,320	66,400,411	-0.032	0.126	88,601	175,821	175,824	4	-0.549	-0.699	2.196
**chr1**	228,225,533	228,225,687	0.030	0.119	21,398	41,960	41,963	4	0.549	0.571	2.194
**chr19**	15,530,606	15,530,870	-0.025	0.100	112,528	226,742	226,745	4	-0.547	-0.653	2.188
**chr3**	42,307,519	42,307,866	0.024	0.097	151,540	303,761	303,764	4	0.544	0.672	2.175
**chr1**	159,825,552	159,825,761	0.028	0.112	16,043	32,151	32,154	4	0.540	0.740	2.160
**chr8**	54,569,668	54,570,293	0.031	0.124	217,968	440,066	440,069	4	0.536	0.569	2.143
**chr5**	493,262	493,746	0.036	0.182	172,643	343,608	343,612	5	0.424	0.485	2.121
**chr6**	29,795,501	29,795,595	0.034	0.136	188,094	374,395	374,398	4	0.525	0.586	2.101
**chr17**	80,289,500	80,289,701	-0.027	0.107	104,929	210,429	210,432	4	-0.520	-0.603	2.081
**chr10**	70,321,770	70,321,959	0.031	0.124	28,175	54,833	54,836	4	0.519	0.636	2.075
**chr12**	117,797,056	117,797,635	0.040	0.200	57,778	115,485	115,489	5	0.413	0.545	2.064
**chr19**	11,353,961	11,354,240	0.028	0.113	111,615	224,525	224,528	4	0.514	0.652	2.055
**chr2**	26,624,760	26,624,865	0.025	0.101	121,960	246,995	246,998	4	0.512	0.579	2.048
**chr16**	56,696,748	56,697,229	0.028	0.111	88,038	174,840	174,843	4	0.511	0.680	2.045
**chr4**	140,656,749	140,657,110	0.026	0.103	169,748	338,243	338,246	4	0.510	0.631	2.040
**chr19**	1,387,394	1,387,894	-0.028	0.110	108,823	218,581	218,584	4	-0.509	-0.687	2.036
**chr14**	102,554,826	102,554,977	0.033	0.132	72,843	145,414	145,417	4	0.509	0.647	2.035
**chr6**	10,883,895	10,884,314	0.033	0.130	186,038	369,196	369,199	4	0.508	0.553	2.034
**chr17**	56,565,286	56,565,644	0.027	0.109	100,960	201,742	201,745	4	0.506	0.511	2.025
**chr20**	44,746,392	44,747,006	-0.028	0.285	140,615	282,425	282,434	10	-0.202	-0.249	2.018
**chr6**	146,755,301	146,755,900	0.027	0.110	196,115	396,635	396,638	4	0.500	0.767	1.999
**chr1**	11,708,792	11,709,271	0.033	0.131	3,510	7,279	7,282	4	0.496	0.660	1.983

**chr11**	82,443,149	82,443,614	0.028	0.113	44,506	89,617	89,620	4	0.496	0.675	1.982
**chr1**	17,215,834	17,216,201	0.037	0.147	4,452	8,979	8,982	4	0.494	0.646	1.975
**chr2**	233,251,770	233,252,170	0.041	0.166	135,805	272,015	272,018	4	0.483	0.516	1.933
**chr1**	23,884,703	23,885,086	0.035	0.139	5,630	11,094	11,097	4	0.482	0.511	1.930
**chr8**	143,763,326	143,763,565	-0.028	0.110	223,182	449,333	449,336	4	-0.482	-0.511	1.927
**chr8**	145,008,957	145,009,406	-0.030	0.118	223,731	450,584	450,587	4	-0.481	-0.548	1.925
**chr1**	7,842,159	7,842,406	-0.033	0.132	2,675	5,804	5,807	4	-0.480	-0.577	1.919
**chr20**	61,732,467	61,732,608	0.027	0.106	141,955	285,308	285,311	4	0.476	0.561	1.904
**chr22**	51,016,501	51,016,644	0.025	0.100	148,747	298,687	298,690	4	0.471	0.574	1.885
**chr19**	52,996,083	52,996,617	0.029	0.117	118,026	238,929	238,932	4	0.469	0.534	1.877
**chr1**	227,748,424	227,748,719	0.030	0.120	21,322	41,802	41,805	4	0.466	0.588	1.863
**chr3**	50,487,955	50,488,230	0.025	0.098	152,798	306,618	306,621	4	0.464	0.620	1.856
**chr13**	112,712,424	112,712,795	0.025	0.101	65,563	130,953	130,956	4	0.458	0.570	1.832
**chr1**	228,346,014	228,346,347	0.028	0.112	21,442	42,079	42,082	4	0.458	0.540	1.830
**chr19**	19,639,553	19,639,596	0.033	0.131	113,496	228,856	228,859	4	0.455	0.615	1.819
**chr22**	46,449,498	46,449,821	-0.031	0.124	147,948	297,262	297,265	4	-0.453	-0.486	1.811
**chr2**	114,033,360	114,033,830	0.028	0.112	128,333	258,706	258,709	4	0.448	0.454	1.792
**chr20**	5,485,144	5,485,294	-0.030	0.181	138,494	277,554	277,559	6	-0.298	-0.320	1.787
**chr8**	56,791,576	56,791,798	-0.033	0.132	218,114	440,360	440,363	4	-0.446	-0.476	1.785
**chr6**	31,148,404	31,148,483	-0.025	0.124	188,606	377,487	377,491	5	-0.349	-0.373	1.747
**chr13**	112,187,145	112,187,396	0.030	0.121	65,453	130,675	130,678	4	0.419	0.484	1.676
**chr17**	73,584,029	73,584,111	0.028	0.141	102,695	205,201	205,205	5	0.335	0.522	1.673
**chr20**	44,803,246	44,803,686	0.032	0.128	140,625	282,446	282,449	4	0.416	0.476	1.663

**chr15**	81,410,745	81,411,066	0.031	0.126	79,970	158,935	158,938	4	0.416	0.507	1.662
**chr7**	27,138,712	27,138,974	0.026	0.105	202,131	409,513	409,516	4	0.415	0.493	1.661
**chr6**	32,055,135	32,055,316	0.027	0.107	188,977	380,461	380,464	4	0.411	0.627	1.643
**chr12**	6,486,598	6,486,709	-0.024	0.098	49,523	99,109	99,112	4	-0.411	-0.567	1.643
**chr12**	53,358,946	53,359,506	0.030	0.118	53,044	105,984	105,987	4	0.410	0.466	1.640
**chr12**	132,939,657	132,939,992	0.028	0.114	60,038	120,334	120,337	4	0.409	0.455	1.638
**chr2**	66,659,348	66,659,590	0.025	0.098	124,840	252,149	252,152	4	0.404	0.514	1.615
**chr16**	54,321,848	54,322,494	0.027	0.108	87,801	174,341	174,344	4	0.401	0.574	1.602
**chr17**	43,716,423	43,716,617	-0.024	0.098	99,443	198,608	198,611	4	-0.393	-0.451	1.570
**chr5**	496,069	496,476	0.035	0.141	172,645	343,618	343,621	4	0.392	0.628	1.568
**chr9**	96,715,687	96,716,209	0.025	0.101	226,639	455,043	455,046	4	0.386	0.424	1.543
**chr4**	80,885,735	80,886,264	0.024	0.097	167,324	333,738	333,741	4	0.378	0.548	1.514
**chr6**	37,616,410	37,616,803	0.027	0.107	190,315	385,855	385,858	4	0.378	0.556	1.512
**chr16**	4,103,167	4,103,533	-0.026	0.103	84,029	167,363	167,366	4	-0.372	-0.470	1.486
**chr16**	55,794,456	55,794,910	0.033	0.164	87,927	174,589	174,593	5	0.272	0.363	1.361
**chr9**	34,370,781	34,370,894	0.025	0.099	224,994	452,784	452,787	4	0.336	0.491	1.345
**chr22**	26,875,499	26,875,652	-0.025	0.102	145,844	292,777	292,780	4	-0.336	-0.374	1.342
**chr4**	940,614	941,054	0.029	0.115	162,328	323,947	323,950	4	0.333	0.414	1.330
**chr7**	4,901,337	4,901,628	0.026	0.105	200,602	406,489	406,492	4	0.330	0.366	1.318
**chr17**	81,045,495	81,045,863	0.025	0.101	105,350	211,445	211,448	4	0.320	0.590	1.281
**chr6**	292,329	292,823	-0.031	0.157	184,828	366,824	366,828	5	-0.250	-0.260	1.252
**chr7**	4,832,112	4,832,359	0.030	0.119	200,565	406,401	406,404	4	0.309	0.356	1.236
**chr5**	139,227,979	139,228,242	0.038	0.192	180,529	358,423	358,427	5	0.247	0.311	1.236

**chr11**	2,406,712	2,407,267	-0.027	0.109	36,227	72,232	72,235	4	-0.292	-0.551	1.168
**chr11**	6,592,066	6,592,745	0.024	0.096	36,931	73,912	73,915	4	0.289	0.321	1.157
**chr5**	664,363	664,666	-0.035	0.139	172,747	343,836	343,839	4	-0.287	-0.317	1.147
**chr3**	194,705,841	194,706,168	-0.030	0.121	161,493	322,192	322,195	4	-0.274	-0.334	1.096
**chr21**	46,975,805	46,976,340	0.027	0.110	144,394	290,012	290,015	4	0.264	0.404	1.055
**chr6**	31,237,029	31,237,405	0.045	0.179	188,622	377,561	377,564	4	0.232	0.238	0.928
**chr19**	55,477,653	55,477,810	-0.030	0.120	118,579	240,113	240,116	4	-0.164	-0.198	0.655
**chr2**	128,453,108	128,453,484	0.034	0.171	129,225	260,338	260,342	5	0.114	0.157	0.571
**chr4**	6,728,936	6,729,199	0.035	0.141	163,790	327,281	327,284	4	0.128	0.205	0.513
**chr12**	131,622,284	131,622,739	-0.028	0.111	59,596	119,302	119,305	4	-0.085	-0.163	0.340

The table shows the results of the *dmrFind* algorithm. DMR positions are shown in the chr/start/end co-ordinates, with probe indices and numbers represented by indexStart, indexEnd and nprobes, and area_raw the significance calculation following permutation analysis, allowing ranking of these DMRs by significance, as shown.
