# Supplementary material for: Mosaic Epigenetic Dysregulation of Ectodermal Cells in Autism Spectrum Disorder
Source: PLoS Genet. 2014 May 29;10(5):e1004402. doi: 10.1371/journal.pgen.1004402 (PMC4038484; doi:10.1371/journal.pgen.1004402)
Supplement: Table S7 — Bonferroni-corrected p-values of association between modules and known covariates. (PDF) [file pgen.1004402.s015.pdf]

**Supplemental Table S7: Bonferroni-corrected p-values of association between modules and known covariates**

| Module | ASD | Gender | Age | YRI % | CEU % |
|---|---|---|---|---|---|
| MElavenderblush2 | 1 | 1 | 1 | 1 | 1 |
| Meplum3 | 1 | 1 | 1 | 1 | 1 |
| Mecoral2 | 1 | 1 | 1 | 1 | 1 |
| Methistle3 | 1 | 1 | 1 | 1 | 1 |
| Memediumorchid | 1 | 1 | 1 | 1 | 1 |
| Medeeppink | 1 | 1 | 1 | 1 | 1 |
| Melightslateblue | 1 | 1 | 1 | 1 | 1 |
| Mepaleturquoise | 1 | 1 | 1 | 1 | 1 |
| Mepalevioletred3 | 1 | 1 | 1.38E-02 | 1 | 1 |
| Mechocolate4 | 1 | 1 | 3.18E-04 | 1 | 1 |
| Memediumpurple1 | 1 | 1 | 1 | 1 | 1 |
| Menavajowhite1 | 1 | 1 | 9.65E-02 | 1 | 1 |
| Medarkseagreen3 | 1 | 1 | 4.47E-02 | 1 | 1 |
| Mepink4 | 1 | 1 | 7.86E-02 | 1 | 1 |
| Mehoneydew | 1 | 1 | 5.09E-04 | 1 | 1 |
| Mesaddlebrown | 0.65 | 1 | 3.67E-03 | 1 | 1 |
| Mepalevioletred2 | 1 | 1 | 3.04E-03 | 1 | 1 |
| Methistle1 | 2.43E-02 | 1 | 7.58E-05 | 1 | 1 |
| Memagenta4 | 0.16 | 1 | 3.09E-17 | 1 | 1 |
| Meyellow | 8.50E-04 | 1 | 8.40E-16 | 2.53E-02 | 1.98E-02 |
| Mefirebrick3 | 8.23E-02 | 1 | 2.31E-08 | 1 | 0.88 |
| Mepalevioletred1 | 2.85E-02 | 1 | 1.73E-10 | 2.06E-02 | 1.38E-02 |
| Meplum2 | 1 | 1 | 4.00E-09 | 1 | 1 |
| Melightsteelblue1 | 1 | 1 | 3.61E-15 | 0.59 | 0.74 |
| Menavajowhite2 | 0.21 | 1 | 5.86E-11 | 0.33 | 0.51 |
| Mecoral3 | 1 | 1 | 4.13E-15 | 1 | 1 |
| Mebrown | 9.93E-03 | 1 | 9.61E-28 | 1.27E-02 | 1.40E-02 |
| Meskyblue1 | 1 | 1 | 3.32E-23 | 1 | 1 |
| Mecoral4 | 2.31E-02 | 1 | 2.30E-03 | 1 | 1 |
| Mecyan | 1 | 1 | 1 | 1 | 1 |
| Metan4 | 5.92E-02 | 1 | 1.55E-04 | 1 | 1 |
| Mehoneydew1 | 1 | 1 | 3.28E-02 | 1 | 1 |
| Melightcoral | 1 | 1 | 1.12E-04 | 0.23 | 1 |
| Meorange | 1 | 1 | 1.64E-08 | 9.54E-02 | 0.280779828 |
| Meantiquewhite2 | 1 | 1 | 1 | 1 | 1 |
| Megreen | 1 | 1 | 9.28E-03 | 1 | 1 |
| Meyellow4 | 1 | 1 | 4.17E-06 | 1 | 1 |
| Meblue | 1 | 1 | 3.95E-06 | 1 | 1 |
| Meturquoise | 1 | 1 | 8.25E-06 | 1 | 1 |
| Medarkseagreen4 | 1 | 1 | 1 | 1 | 1 |
| Medarkgreen | 1 | 1 | 1 | 1 | 1 |

| | | | | | |
|---|---|---|---|---|---|
| Mefloralwhite | 1 | 0.92 | 1 | 6.57E-03 | 2.44E-04 |
| Meorangered4 | 1 | 1 | 1 | 1 | 1 |
| Meblue4 | 1 | 1 | 1 | 1 | 1 |
| Megreen4 | 1 | 1 | 1 | 1 | 1 |
| Melightsteelblue | 1 | 1 | 1 | 1 | 1 |
| Meskyblue3 | 1 | 1 | 1 | 1 | 1 |
| Mesalmon2 | 1 | 1 | 1 | 1 | 1 |
| Meskyblue2 | 1 | 1 | 1 | 1 | 1 |
| Mesienna3 | 1 | 1 | 1 | 1 | 1 |
| Meorangered3 | 1 | 1 | 1 | 1 | 1 |
| Meplum1 | 1 | 1 | 1 | 1 | 1 |
| Meyellowgreen | 1 | 1 | 1 | 1 | 1 |
| Mesienna4 | 1 | 1 | 1 | 1 | 1 |
| Mesteelblue | 1 | 1 | 1 | 1 | 1 |
| Meindianred4 | 1 | 1 | 1 | 1 | 1 |
| Melavenderblush1 | 1 | 1 | 1 | 1 | 1 |
| Medarkslateblue | 1 | 1 | 1 | 1 | 1 |
| Meskyblue4 | 1 | 1 | 1 | 1 | 1 |
| Mesalmon1 | 1 | 1 | 1 | 1 | 1 |
| Mewhite | 1 | 1 | 1 | 1 | 1 |
| Memidnightblue | 1 | 1 | 1 | 1 | 1 |
| Methistle2 | 1 | 1 | 1 | 1 | 1 |
| Medarkviolet | 1 | 1 | 1 | 1 | 1 |
| **Melightgreen** | **5.26E-02** | **1** | **1** | **1** | **1** |
| Meskyblue | 1 | 1 | 1 | 1 | 1 |
| Meyellow3 | 1 | 1 | 1 | 1 | 1 |
| Medarkseagreen2 | 1 | 1 | 1 | 1 | 1 |
| Meorangered1 | 1 | 1 | 1 | 1 | 1 |
| Meivory | 1 | 1 | 1 | 1 | 1 |
| Medarkolivegreen | 1 | 1 | 1 | 1 | 1 |
| Memagenta3 | 1 | 1 | 1 | 1 | 1 |
| Melightcyan | 1 | 1 | 1 | 4.30E-02 | 0.59 |
| Melightpink4 | 1 | 1 | 1 | 1 | 1 |
| Meroyalblue | 1 | 1 | 1 | 1 | 0.10 |
| Mecoral | 1 | 1 | 1 | 1 | 1 |
| Medarkgrey | 1 | 1 | 1 | 1 | 1 |
| Memagenta | 1 | 1 | 1 | 0.19 | 0.64 |
| Melavenderblush3 | 1 | 1 | 1 | 1 | 1 |
| Mefirebrick4 | 1 | 1 | 1 | 1 | 1 |
| Methistle4 | 1 | 1 | 0.28 | 1 | 1 |
| Medarkmagenta | 1 | 1 | 1 | 0.67 | 0.24 |
| **Medarkolivegreen2** | **1.01E-02** | **1** | **0.18** | **0.42** | **0.54** |
| Memediumpurple2 | 1 | 1 | 1 | 1 | 1 |
| Meviolet | 1 | 1 | 1 | 1 | 1 |

| | | | | | |
|---|---|---|---|---|---|
| Meblack | 1 | 1 | 1 | 5.83E-02 | 0.47 |
| Megreenyellow | 1 | 1 | 1 | 3.28E-03 | 3.61E-02 |
| Memaroon | 1 | 0.64 | 1 | 0.59 | 0.82 |
| Mepurple | 0.51 | 1.08E-42 | 1 | 1 | 1 |
| Meantiquewhite4 | 1 | 1 | 1 | 1 | 1 |
| Meblueviolet | 1 | 1 | 1 | 1 | 1 |
| Meplum4 | 1 | 1 | 1 | 1 | 1 |
| Memediumpurple3 | 1 | 1 | 1 | 1 | 1 |
| Menavajowhite | 1 | 1 | 1 | 1 | 1 |
| Mesalmon4 | 1 | 1 | 1 | 1 | 1 |
| Meindianred3 | 1 | 1 | 1 | 1 | 1 |
| Meblue2 | 1 | 1 | 1 | 1 | 1 |
| Mecoral1 | 1 | 1 | 1 | 1 | 1 |
| Meplum | 1 | 1 | 1 | 1 | 1 |
| Mered | 1 | 1 | 1 | 5.97E-03 | 3.75E-02 |
| Mesalmon | 1 | 1 | 1 | 6.59E-02 | 0.39877902 |
| Melightblue4 | 1 | 1 | 1 | 1 | 1 |
| Mebrown2 | 0.91 | 1 | 1 | 1 | 1 |
| Medarkturquoise | 4.07E-02 | 1 | 8.32E-29 | 1 | 1 |
| Melightyellow | 3.68E-02 | 1 | 2.75E-20 | 1 | 1 |
| Medarkorange2 | 1 | 1 | 6.18E-04 | 1 | 1 |
| Metan | 1 | 1 | 2.51E-09 | 1 | 1 |
| Melightcyan1 | 1 | 1 | 6.23E-02 | 1 | 1 |
| Mebisque4 | 1 | 1 | 1 | 1 | 1 |
| Memediumpurple4 | 1 | 1 | 1 | 1 | 1 |
| Meantiquewhite1 | 1 | 1 | 1.42E-03 | 1 | 1 |
| Medarkolivegreen4 | 0.38 | 1 | 1.13E-05 | 0.99 | 0.47 |
| Medarkorange | 2.48E-02 | 1 | 1.26E-07 | 1 | 1 |
| Melightpink3 | 1 | 1 | 1 | 1 | 1 |
| Medarkred | 1 | 1 | 1 | 1 | 1 |
| Megrey60 | 1 | 1 | 1 | 0.19 | 1 |
| Mebrown4 | 1 | 1 | 1 | 1 | 1 |
| Mepink | 1 | 1 | 1 | 2.08E-02 | 0.15 |
| Melightpink2 | 1 | 1 | 1 | 1 | 1 |
| Methistle | 1 | 1 | 1.75E-02 | 1 | 1 |
| Megrey | 6.01E-05 | 1 | 2.81E-30 | 4.72E-03 | 3.28E-03 |
