# Supplementary material for: Mosaic Epigenetic Dysregulation of Ectodermal Cells in Autism Spectrum Disorder
Source: PLoS Genet. 2014 May 29;10(5):e1004402. doi: 10.1371/journal.pgen.1004402 (PMC4038484; doi:10.1371/journal.pgen.1004402)
Supplement: Table S8 — Genes in light green module associated with ASD. (PDF) [file pgen.1004402.s016.pdf]

Supplemental Table S8: Genes in *light green* module associated with ASD

Gene	Chromosome	Position	Module Membership (MM)	MM P-value
HES4	1	936610	0.80	6.35E-22
ALDH4A1	1	19229528	0.76	9.95E-19
GRIK3	1	37498270	0.72	8.16E-16
DPH2	1	44435457	0.70	1.47E-14
EIF2B3	1	45452166	0.73	9.48E-17
RAD54L	1	46712932	0.79	1.18E-20
C1orf183	1	112281877	0.88	2.41E-31
ST7L	1	113162073	0.77	2.17E-19
CAPZA1	1	113162073	0.77	2.17E-19
DENND2C	1	115212659	0.85	8.00E-27
LGR6	1	202162209	0.85	5.85E-27
PTPN14	1	214725274	0.78	8.04E-20
MORN2	2	39103277	0.79	1.38E-20
DHX57	2	39103277	0.79	1.38E-20
MGAT5	2	134949571	0.76	1.67E-18
RIF1	2	152266336	0.80	9.07E-22
RPE	2	210867059	0.72	3.05E-16
IGFBP5	2	217559020	0.74	2.99E-17
SCAP	3	47516975	0.77	4.27E-19
TNNC1	3	52489346	0.71	3.55E-15
NISCH	3	52489346	0.71	3.55E-15
MAGI1	3	66024691	0.72	6.66E-16
NAALADL2	3	174095241	-0.71	1.86E-15
HES1	3	193852754	0.76	2.91E-18
MAN2B2	4	6577027	0.76	2.38E-18
HS3ST1	4	11810667	0.76	2.21E-18
CD38	4	15780522	0.73	1.31E-16
RBPJ	4	26323246	0.82	4.72E-23
FRAS1	4	78977690	0.76	1.78E-18
FRAS1	4	78978133	0.74	3.84E-17
COQ2	4	84205961	0.77	2.09E-19
LARP7	4	113626831	0.71	1.34E-15
SMAD1	4	146296778	-0.72	6.74E-16
HMGB2	4	174254825	0.73	1.90E-16
RGS7BP	5	63802184	0.78	4.86E-20
FAM151B	5	79783889	0.72	9.98E-16
PRDM6	5	122435202	0.75	5.20E-18
NEUROG1	5	134871686	0.72	3.06E-16
SPARC	5	151031796	0.72	3.59E-16
TSPAN17	5	176131088	-0.79	7.70E-21
ZFP62	5	180287685	0.80	2.45E-21
LY86	6	6648823	-0.70	6.61E-15

BAT4	6	31634141	0.70	5.78E-15
CSNK2B	6	31634141	0.70	5.78E-15
PPIL1	6	36842651	0.81	7.24E-23
CRIP3	6	43276478	0.74	2.91E-17
LCA5	6	80246572	0.84	5.79E-26
NDUFAF4	6	97345972	0.70	9.24E-15
C6orf174	6	127837548	0.75	6.74E-18
TNRC18	7	5463409	0.71	1.93E-15
IGFBP3	7	45961943	0.76	3.38E-18
TYW1B	7	72298667	0.72	4.62E-16
SBDSP	7	72298667	0.72	4.62E-16
GATSL1	7	74379144	-0.69	1.63E-14
LHFPL3	7	103969483	0.71	2.38E-15
LHFPL3	7	103970195	0.70	5.50E-15
TSPAN12	7	120497479	0.72	3.42E-16
RHEB	7	151215566	0.80	7.33E-22
SORBS3	8	22423994	0.78	1.07E-19
TNFRSF10B	8	22926800	0.71	2.51E-15
DPYSL2	8	26434689	0.72	5.60E-16
RRM2B	8	103251909	0.70	1.30E-14
MYC	8	128748155	0.73	1.62E-16
PCSK5	9	78506874	0.79	3.30E-21
ZEB1	10	31608136	0.75	1.02E-17
FZD8	10	35930499	0.72	3.60E-16
ZNF503-AS1	10	77054788	0.70	5.06E-15
FGF8	10	103535362	0.78	1.15E-19
ZNF215	11	6948101	0.80	9.54E-22
GTF2H1	11	18343657	0.74	4.99E-17
HPS5	11	18343657	0.74	4.99E-17
PAX6	11	31831591	0.72	7.24E-16
KBTBD4	11	47600851	0.73	1.87E-16
NDUFS3	11	47600851	0.73	1.87E-16
FADS1	11	61584442	0.76	2.55E-18
VEGFB	11	64002754	0.70	1.51E-14
BAD	11	64052221	0.79	6.58E-21
GPR137	11	64052221	0.79	6.58E-21
KRTAP5-11	11	71340352	-0.81	2.92E-22
GAB2	11	78129288	0.77	4.88E-19
TMEM126B	11	85339628	0.78	2.11E-20
DLG2	11	85339628	0.78	2.11E-20
MPZL3	11	118123074	0.71	2.29E-15
CLEC4C	12	7904267	-0.73	7.50E-17
DDX11	12	31226536	0.75	1.16E-17
NELL2	12	45270304	0.74	2.41E-17

TRHDE	12	72667326	0.71	2.35E-15
LOC283392	12	72667326	0.71	2.35E-15
N4BP2L1	13	33002431	0.78	2.70E-20
ABCC4	13	95953574	0.75	1.21E-17
ARHGEF7	13	111768023	0.77	5.42E-19
TUBGCP3	13	113263221	-0.76	2.00E-18
EFS	14	23834995	0.75	4.72E-18
PSME1	14	24604912	0.71	2.82E-15
SNX6	14	35099518	0.74	6.03E-17
SRP54	14	35451984	0.70	5.47E-15
FOXN3	14	90084672	0.74	4.68E-17
SLC25A29	14	100751514	0.75	4.31E-18
PACS2	14	105827276	-0.85	5.52E-27
RTF1	15	41708917	0.78	2.32E-20
CORO2B	15	68870836	0.74	1.96E-17
CSPG4	15	75986363	0.71	2.67E-15
ZNF200	16	3285262	0.74	5.77E-17
KLHDC4	16	87811505	0.76	2.23E-18
RNMTL1	17	685915	0.71	1.85E-15
GLOD4	17	685915	0.71	1.85E-15
NF1	17	29421732	0.75	4.54E-18
SLFN11	17	33700513	0.72	5.32E-16
COPZ2	17	46114574	0.79	3.32E-21
MIR152	17	46114574	0.79	3.32E-21
LOC146880	17	62777690	0.76	1.34E-18
KPNA2	17	66031814	0.72	8.83E-16
RECQL5	17	73629082	0.74	3.28E-17
LOC643008	17	73629082	0.74	3.28E-17
TIMP2	17	76921528	0.80	2.29E-21
CCDC165	18	8707237	0.77	1.28E-19
RAB27B	18	52495848	0.70	6.23E-15
CCDC102B	18	66382471	0.78	3.46E-20
TMX3	18	66382471	0.78	3.46E-20
SGTA	19	2761892	0.70	6.43E-15
ZNF77	19	2945000	0.80	5.98E-22
TNPO2	19	12833533	0.71	4.20E-15
LYL1	19	13213716	0.71	1.22E-15
UQCRFS1	19	29704262	0.74	6.92E-17
RTN2	19	45996498	0.74	6.33E-17
SMOX	20	4129314	0.73	1.77E-16
HMGN1	21	40720919	0.73	9.04E-17
DGCR6	22	18893614	0.71	1.42E-15
LZTR1	22	21337040	0.81	2.81E-22
PNPLA5	22	44287772	0.77	1.49E-19
