# Supplementary material for: Mosaic Epigenetic Dysregulation of Ectodermal Cells in Autism Spectrum Disorder
Source: PLoS Genet. 2014 May 29;10(5):e1004402. doi: 10.1371/journal.pgen.1004402 (PMC4038484; doi:10.1371/journal.pgen.1004402)
Supplement: Table S9 — Genes in dark olive green module associated with ASD. (PDF) [file pgen.1004402.s017.pdf]

Supplemental Table S9: Genes in *dark olive green* module associated with ASD

Gene	Chromosome	Position	Module Membership (MM)	MM P-value
PEX10	1	2337334	0.810337844	1.32E-22
GPBP1L1	1	46106399	0.773502529	1.61E-19
BCAR3	1	94245226	0.740245636	3.41E-17
ZC3H11A	1	203763483	0.768457607	3.85E-19
ZC3H11A	1	203763498	0.699133215	9.15E-15
CAMKMT	2	44646510	0.830944239	1.21E-24
PRKCE	2	46121488	0.728533494	1.86E-16
RBMS1	2	161593271	0.776332535	9.80E-20
ARPC2	2	219079038	0.797400033	1.89E-21
BSN	3	49638532	0.845041711	3.35E-26
CPLX1	4	795538	0.702505454	5.99E-15
NFXL1	4	47854334	0.802345605	7.00E-22
C5orf27	5	95192949	0.70421452	4.83E-15
ERGIC1	5	172263112	0.683902398	5.75E-14
TAP2	6	32803058	0.778753197	6.37E-20
COL11A2	6	33151008	0.729478157	1.63E-16
WDR46	6	33254880	0.801745369	7.91E-22
WDR46	6	33254892	0.761165479	1.30E-18
RSPH3	6	159423743	0.769393089	3.28E-19
AGPAT4	6	161549519	0.790267496	7.57E-21
MACC1	7	20240145	0.698674927	9.68E-15
CUX1	7	101478535	0.802229538	7.17E-22
JHDM1D	7	139859464	0.713418296	1.47E-15
KIAA0146	8	48557420	0.792246082	5.18E-21
KIAA0146	8	48587440	0.753484619	4.48E-18
PABPC1	8	101802144	0.774123593	1.45E-19
ARFIP2	11	6499575	0.736923523	5.57E-17
MICAL2	11	12222570	0.722898685	4.08E-16
CNIH2	11	66048759	0.805030303	4.03E-22
MAML2	11	95889454	0.781796778	3.67E-20
KRT79	12	53228661	0.764105173	8.00E-19
NCOR2	12	125030744	0.722336993	4.41E-16
ATP11A	13	113348391	0.730182655	1.47E-16
HOMEZ	14	23744304	0.747121013	1.21E-17
ARHGAP5	14	32597733	0.831457161	1.07E-24
RBM25	14	73524288	0.757192347	2.48E-18
c15orf50	15	70147094	0.763106126	9.44E-19
MLYCD	16	83945978	0.776011546	1.04E-19
ZNF426	19	9645566	0.749726963	8.08E-18
PSMD8	19	38869646	0.801710907	7.97E-22
HM13	20	30126382	0.728729016	1.81E-16
PPM1F	22	22290866	0.710229687	2.23E-15
