# Supplementary material for: Mosaic Epigenetic Dysregulation of Ectodermal Cells in Autism Spectrum Disorder
Source: PLoS Genet. 2014 May 29;10(5):e1004402. doi: 10.1371/journal.pgen.1004402 (PMC4038484; doi:10.1371/journal.pgen.1004402)
Supplement: Text S1 — A file containing all supplementary figures and tables, with details on analytical approaches and including software allowing re-analysis of data. (PDF) [file pgen.1004402.s018.pdf]

Mosaic ectodermal epigenetic dysregulation in autism spectrum disorder.

Berko *et al.*

Table of contents:

Page	Description
2	Patient cohort
3	Sample collection
4	Genotyping and methylation microarrays
5	Genotyping data preprocessing
5	Local ancestry deconvolution
7	Mosaicism detection
9	Methylation data preprocessing
9	Differentially-methylated region (DMR) identification
14	Copy number variant (CNV) calling
14	Massively-Parallel Bisulfite Sequencing to verify differential methylation
16	Gene ontology enrichment analysis
31	Weighted Gene Co-expression Network Analysis (WGCNA)
32	Protein-Protein Interaction (PPI) analysis
41	Code
44	Supplemental references

Illustrations

Page	Description
2	Supplemental Table S1
3	Supplemental Figure S1
4	Supplemental Table S2
6	Supplemental Table S3
8	Supplemental Figure S2
10	Supplemental Figure S3
11	Supplemental Figure S4
12	Supplemental Table S4
13	Supplemental Figure S5
14	Supplemental Table S5
15	Supplemental Figure S6
16	Supplemental Figure S7
17	Supplemental Table S6
33	Supplemental Figure S8
34	Supplemental Table S7
37	Supplemental Table S8
40	Supplemental Table S9

Patient Cohort

All patient recruitment and sample collection was done with the appropriate human subjects protocol approval from the Institutional Review Board at the Albert Einstein College of Medicine.

We enrolled two groups of subjects: descriptive traits of the two groups are summarized in **Supplemental Table S1.**

We recruited subjects with an ASD by two primary methods: enrolling patients seen in our clinics, and recruiting patients nationally from advertisements and a posting on the Autism Speaks website (http://www.autismspeaks.org). Patients enrolled from our clinics and research labs (43 subjects) were diagnosed with an ASD by combinations of metrics including the ADOS, ADI-R, CARS, DSM-IV, and clinical assessment, by clinicians and researchers trained in ASD diagnostic measures.

Other subjects enrolled in our study by internet and external recruitment (7 subjects) provided reports of an ASD diagnosis by ADOS and/or ADI-R from an accredited institution with one exception, a patient diagnosed with an ASD by the New York City Early Intervention Program.

The control cohort consisted of typically developing individuals without any prior evidence of an autism spectrum disorder.

	**ASD**	**TD**
Ages		
Maternal age (mean, SD)	37.58, 2.93	38.1, 2.97
(range)	(35-48)	(35-48)
Paternal age	40.19, 6.27	40.72, 5.87
	(29-51)	(30-52)
Subject Age	6.84, 3.58	11.2, 7.31
	(2-17)	(1-28)
Gender		
Male (n)	39	22
Female	11	28
Genetic Ancestry		
% CEU (mean, SD)	63.38, 29.37	85.19, 16.38
% YRI	22.43, 30.74	6.61, 17.02
**Total**	**50**	**50**

Supplemental Table S1: Characteristics of study subjects.

Age metrics reflect the mean age in years per group and the standard deviation, with the range included in parentheses. Maternal and paternal age refer to the age of the parents at the time of the subject's birth. All maternal ages are included, while information for paternal age was only available for ~50% of subjects. Subject age refers to age when the sample was collected. Percent genetic ancestry based on subjects with quality filtered genotype data; 47 ASD and 46 TD genotypes were included.

ASD: Autism Spectrum Disorder.
TD: Typically Developing.
CEU: Utah residents with Northern/Western European ancestry from the CEPH collection (European)
YRI: Yoruba in Ibadan, Nigeria (African)

Sample Collection

We optimized a method to collect DNA from buccal epithelium, using brushes to exfoliate the cheek and cheek gutters (area between the upper/lower gumline and cheeks). Brushes were then placed in CytoLyt (Hologic, MA), a methanol-based preservative. Upon arrival in the laboratory, brushes were removed from the solution, and cell pellets were retrieved from the sample, snap frozen in liquid nitrogen, and moved to -80°C for long term storage.

Cell subtype composition was determined by preparing slides from each of the samples collected, performing haematoxylin and eosin (H&E) staining, and visualizing using light microscopy, counting the first 25 individual (not clumped) cells seen. The typical appearance of these cells plus examples of cell-like structures counted as contaminants in **Supplemental Figure S1a-b**. Comparably high purity of the squamous epithelial cells was found for both groups.

We performed DNA extraction from these cells and confirmed the presence of high quality and high molecular weight DNA with UV spectrometry and gel electrophoresis.

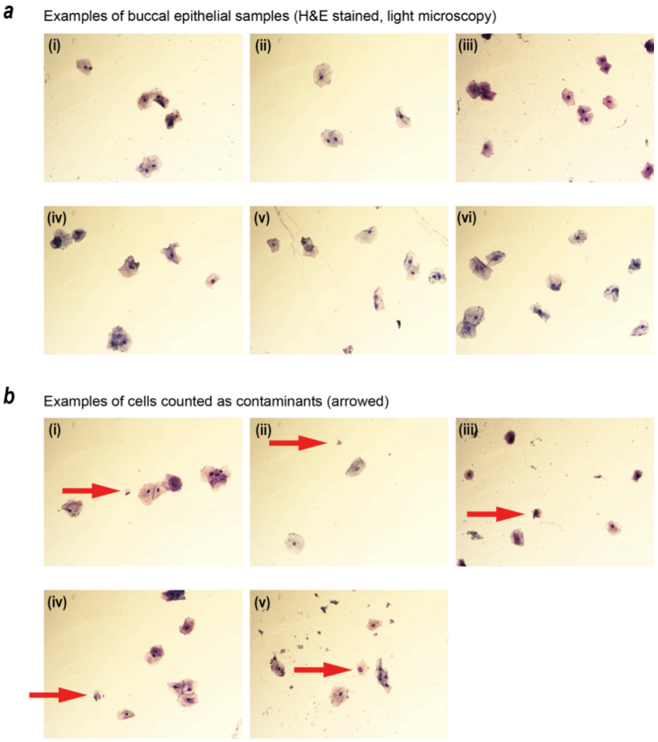

c Percentages of buccal epithelial cells in each cohort (25 cells counted per sample)

	Median	Mean	Standard deviation
Typically developing (TD)	100	97.96	2.35
Autism spectrum disorder (ASD)	100	97.86	2.52

Supplemental Figure S1: H&E staining of buccal epithelial samples.

The buccal brushings yielded the expected squamous epithelial cells (a) with very small proportions of possible contaminant cells types (b) of different sizes (b.i-b.iv) or nuclear characteristics (b.v). Counts of 25 individual cells per sample showed high purity in both ASD and TD groups (c).

Genotyping and Methylation microarrays

For the epigenome-wide association study (EWAS), we used a microarray-based approach, as the presence of oral bacterial DNA in the extracted samples precluded cost-effective sequencing.

Samples were randomized across arrays, and plates were submitted in batches of 96 to minimize potential for batch effect. Sufficient DNA was extracted to meet manufacturer's requirements of 200 ng and 500 ng for the genotyping and methylation arrays, respectively, Microarray hybridization and subsequent scanning was performed by a core facility according to manufacturer's protocols. The summary of samples run on each microarray is provided in **Supplemental Table S2**.

The Illumina HumanOmni2.5-8 BeadChip genotyping platform was used for genotyping, and the Illumina Infinium HumanMethylation450 Beadchip platform for DNA methylation studies.

	ASD	TD	Parents
Genotyping microarrays			
Batch 1	24	24<br>(including 2 siblings)	47
Batch 2	26	26<br>(including 5 siblings)	45
Methylation microarray			
**Batch 1**	**47**	**48**	**0**

Supplemental Table S2: Experimental batches of microarrays

Genotyping Data Preprocessing

Raw data from the genotyping microarrays were loaded into *BeadStudio*, Illumina's proprietary chip analysis software. Genotype calls were then exported in *plink* format, specifying conversion to the forward strand, which refers to annotation based on dbSNP.

We used *plink* to filter the data, with the following parameters for missingness and allele frequency:

--mind 0.05 --geno 0.1 --maf 0.01 --hwe 0.001

Filtering retained 184 out of 191 individual arrays, and 1,777,521 out of 2,379,855 SNPs, with genotyping rate of 0.992 in these remaining samples.

We then removed all ambiguously mapping single nucleotide polymorphisms (SNPs), designated by Illumina as "chr0".

Since we wanted to perform local ancestry deconvolution based on the 1000 Genomes data, which by convention is annotated on the "+" strand of hg19, we had to ensure our Illumina data likewise all mapped to the "+" strand. We therefore identified which of the dbSNP polymorphisms represented on the array actually mapped to the "–" strand, and used *plink* to flip those bases exclusively. We subsequently further removed the handful of SNPs on each chromosome that still possessed two variant alleles. We confirmed correct strandedness by merging our genotyping data with 1000 Genomes data and performing principal components analysis using *vcftools* (Danecek et al. 2011).

Local Ancestry Deconvolution

Since principal components analysis of our dataset revealed many individuals of mixed 3-way genotype admixture, we chose to perform local ancestry deconvolution with simulated mixed ancestry using *HapMix*, an approach previously shown to be accurate (Price et al. 2009). *HapMix* is an algorithm which uses a hidden Markov model to estimate the probability of ancestry at each data point along a chromosome, based on the input of 2 parental ancestral populations and rates of recombination. To model 3-way admixture, we ran two iterations of *HapMix* with different mixed parental populations, and calculated the joint probability of homozygous or heterozygous genotypes at each point (Price et al. 2009).

As in Price *et al.,* we constructed 2 sets of reference populations using genotype data from the 1000 Genomes project from Caucasians (CEU), Africans (YRI) and East Asians (CJ: CHB + JPT). After randomly selecting 80 unrelated individuals from each group, we constructed two mixed sets: a CEU/(CHB+JPT) set with 40 individuals from each group (80 total), and a CEU/YRI set with 40 individuals from each group (also 80 total). We then ran *HapMix* twice for each chromosome; first with the 2 parental populations as the CEU/(CHB+JPT) mixed set and homogeneous YRI, and then with the 2 parental populations as the CEU/YRI mixed set and homogeneous (CHB+JPT).

For each HapMix run, at every locus, *HapMix* estimates the probability of a homozygous call of the genotype of parental population 1, a heterozygous call of one allele from each parental population, and a homozygous call of the genotype of parental population 2. To calculate the final joint probability from both runs, we employed the following calculations (Price et al. 2009), explained in **Supplemental Table S3**:

RUN 1: CEU/(CHB+JPT) versus YRI as the two potential parental populations

RUN 2: CEU/YRI versus (CHB+JPT) as the two potential parental populations

The probabilities of the six potential states at each locus were normalized to 1, and final ancestry calls were made as the maximum probability at the locus only if it exceeded 0.5.

**Call**	**Probability Calculation (run number)**
Homozygous YRI	Probability YRI (from run 1) **X**<br>Probability CEU/YRI (from run 2)
Homozygous CJ	Probability CEU/(CHB+JPT)(1) **X**<br>Probability (CHB+JPT)(2)
Homozygous CEU	Probability CEU/(CHB+JPT)(1) **X**<br>Probability CEU/YRI(2)
Heterozygous YRI/CJ	Probability heterozygous CEU/(CHB+JPT)& YRI(1) **X**<br>Probability heterozygous CEU/YRI&(CHB+JPT)(2)
Heterozygous YRI/CEU	Probability heterozygous CEU/(CHB+JPT)&YRI(1) **X**<br>Probability CEU/YRI(2)
Heterozygous CJ/CEU	Probability CEU/(CHB+JPT)(1) **X**<br>Probability heterozygous CEU/YRI&CJ(2)

Supplemental Table S3: *HAPMIX* local ancestry probability calculation

Abbreviations:

CHB: Han Chinese in Beijing, China.
JPT: Japanese in Tokyo, Japan
CJ: CHB/JPT (East Asian)
YRI: Yoruba in Ibadan, Nigeria (African)
CEU: Utah residents with Northern and Western European ancestry from the CEPH collection (European)

Mosaicism Detection

We used the *Mosaic Alteration Detection (MAD)* algorithm, implemented in the package *GADA*, to identify potential chromosomal mosaic events (Gonzalez et al. 2011). To confirm our ability to use *MAD* to discover detectable whole chromosome mosaicism, we prepared a test dataset of 4 samples. The samples consisted of DNA from 3 cell lines, known to harbor mosaic trisomy, from Coriell Cell Repository, and one DNA sample extracted from buccal epithelium of a patient with a clinical diagnosis of mosaic trisomy 18. Samples were hybridized on the Illumina Infinium Omni1-Quad (prior to Illumina's release of the 2.5-8 BeadChip) and genotype calls were made with Illumina Genome Studio. We plotted the B-Allele Frequencies (BAF) of each known mosaic chromosome (**Supplemental Figure S2)** confirming the presence of visually detectable mosaic trisomy. From the BAF plots we were further able to characterize the mosaicism as resulting from either a meiosis I or meiosis II non-disjunction, based on loss of the 5th and 6th genotype bands at either the telomere of centromere, respectively. We were also able to identify segments of uniparental disomy (UPD), based on loss of heterozygous bands (Conlin et al. 2010). We then analyzed the genotypes with *MAD*, employing the default suggested parameters (aAlpha=0.8, T=9, and MinSegLen=75). Although it also called mosaic events on other chromosomes, *MAD* correctly identified the whole chromosome mosaic trisomy in all 4 cases, either with an over-abundance of mosaic calls or by calling exceptionally long mosaic regions on the affected chromosome.

To prepare our genotyping dataset for *MAD* analysis, we first re-clustered the data in GenomeStudio to improve the quality of some poorly performing SNPs. After exporting the data and applying the same *plink* filters shown previously, we retained 186 out of 191 individual arrays and 1,820,947 SNPs. We ran MAD with the same default parameters as our test dataset, obtaining 376 called mosaic segments across the 186 individuals. No whole chromosome mosaicism events were detected in either the ASD or the TD subjects.

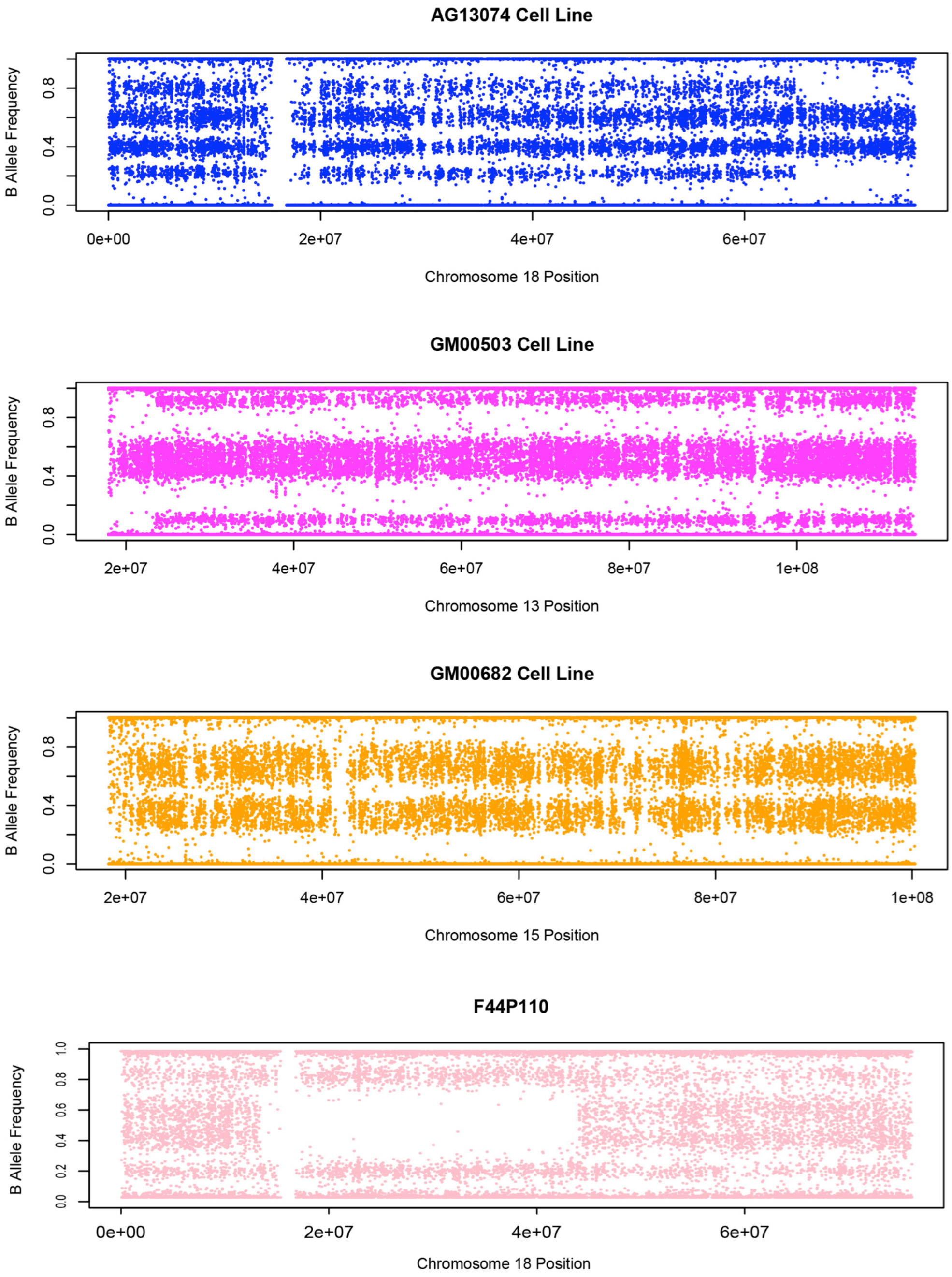

Supplemental Figure S2: B allele frequency plots of known mosaic cases

The *MAD* output correctly highlights an abnormality along chromosomes; analysis of BAF patterns allows determination of the source of error. The pattern in AG13074 results from meiosis I non-disjunction, and F44 P110 demonstrates a meiosis II non-disjunction with mosaic UPD. The lack of array probes on the p arm of chromosome 13 precludes definitive assessment of the meiotic source of the GM00503 error (I or II); the pattern of GM00682 could result from a variety of situations.

Methylation Data Preprocessing

Supplemental Figure S3 provides an overview of the preprocessing pipeline.

Raw *idat* data were loaded in the R (RCoreTeam 2012) package *minfi* (Hansen and Aryee) and assessed for basic quality control metrics, including determination of poorly performing probes with insignificant detection p-values above background control probes. Since our cohort includes both males and females, we removed the X and Y chromosomes from the raw methylation values, and performed *SWAN* normalization to correct for intra-array differences between Illumina Type I and Type II probes (Dedeurwaerder et al. 2011; Maksimovic et al. 2012). We excluded any probes with detection p-value greater than 0.01 (12,590 probes), and then corrected for batch (microarray chip) effect using the *ComBat* function in the R package *SVA* (surrogate variable analysis) (Leek et al.).

Differentially-methylated region (DMR) Identification

To understand the relative effects of known technical, biological, and microarray-based covariates acting on methylation data variability, we performed principal components analysis (PCA) on the M values (logit-transformed Illumina-defined beta values) obtained from the previous preprocessing. We accounted for the possible known confounders, including technical (date of DNA extraction, microarray chip, position on chip), microarray-based (all categories of control probes designed by Illumina) and biological (ASD status, age, gender, and ancestry percentage). Ancestry percent was calculated as the proportion for each population of all allele genotyping positions called by *HapMix*. We fit a linear model for each of the top 10 principal components as a function of each covariate, and summarized the data with a heatmap of the negative $\log_{10}$ p-values for each regression. We identified the significant confounding covariates and corrected for them in all subsequent analysis.

Bump-hunting provides significant advantages over typical individual probe statistical test: it smoothes data over a region, obliterating the need for arbitrary genomic cutoffs, it applies a rigorous analysis and correction for confounding factors, and it incorporates false discovery rate (FDR) considerations as part of the algorithm. Bump-hunting utilizes known covariates input into a model matrix as components in regression analyses. Although we did not see a strong effect of gender on PCA variability, we included gender in our model matrix since methylation patterns are known to vary by gender (Sarter, Long et al. 2005). Based on our PCA data, we input age, percent CEU (European), and percent YRI (African) ancestry as known biological covariates. Bump-hunting was used in the *dmrFind* function in the R package *charm* (Aryee et al. 2011).

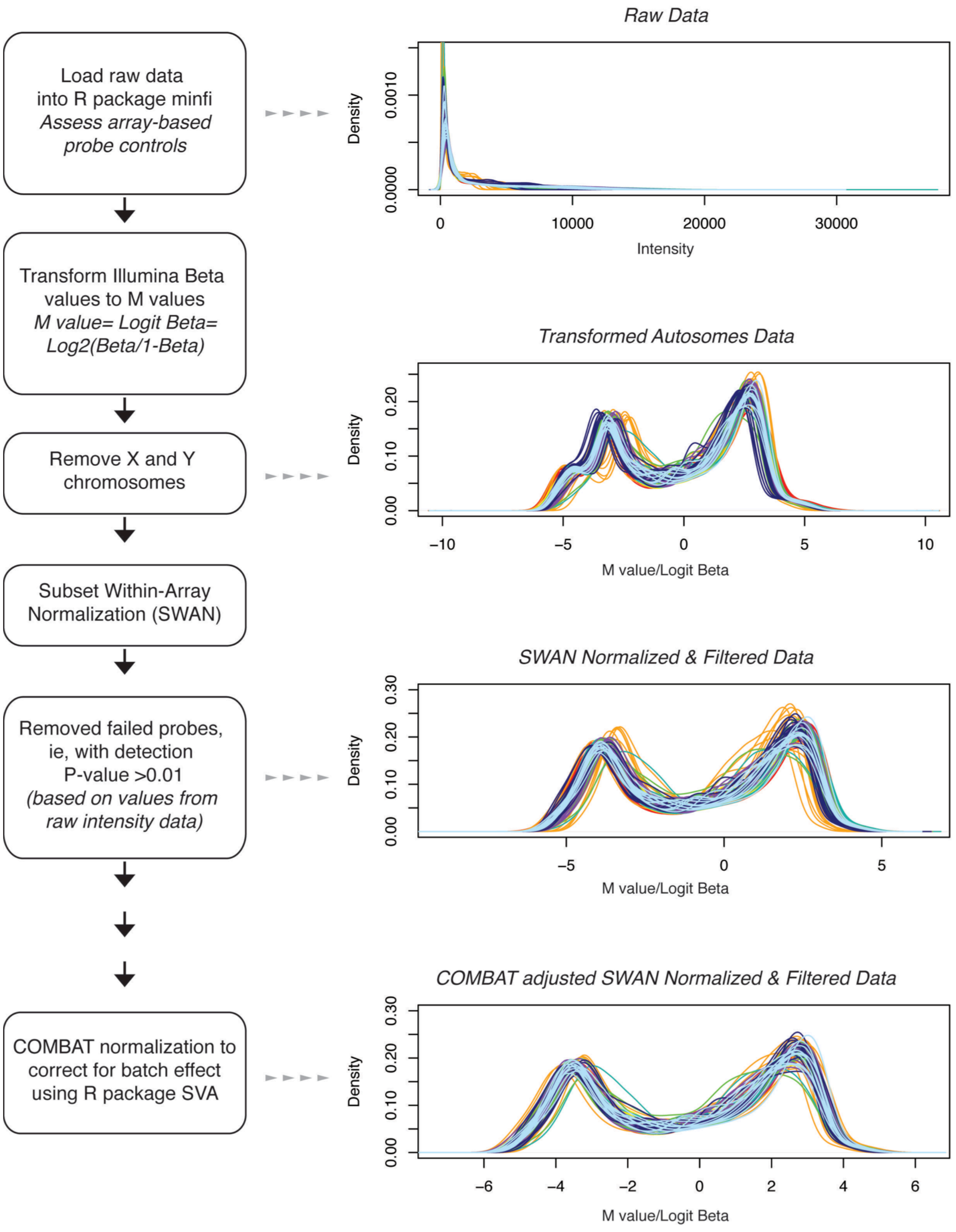

Supplemental Figure S3: Preprocessing of Illumina 450K data

The colors in the density plots correspond to different chips (microarrays), each run to contain 12 samples. The normalization procedures correct for both intra-array and inter-array differences.

We excluded two TD samples from our analysis, since the genotyping arrays performed on their DNA did not pass quality control thresholds.

The *dmrFind* algorithm corrects for unidentified confounders, and returns probe methylation values that preserve the effects of the known covariates input into the model matrix. PCA and linear regression on this data confirmed that SVA properly identified and corrected for all unwanted technical sources of variation, **Supplemental Figure S4**.

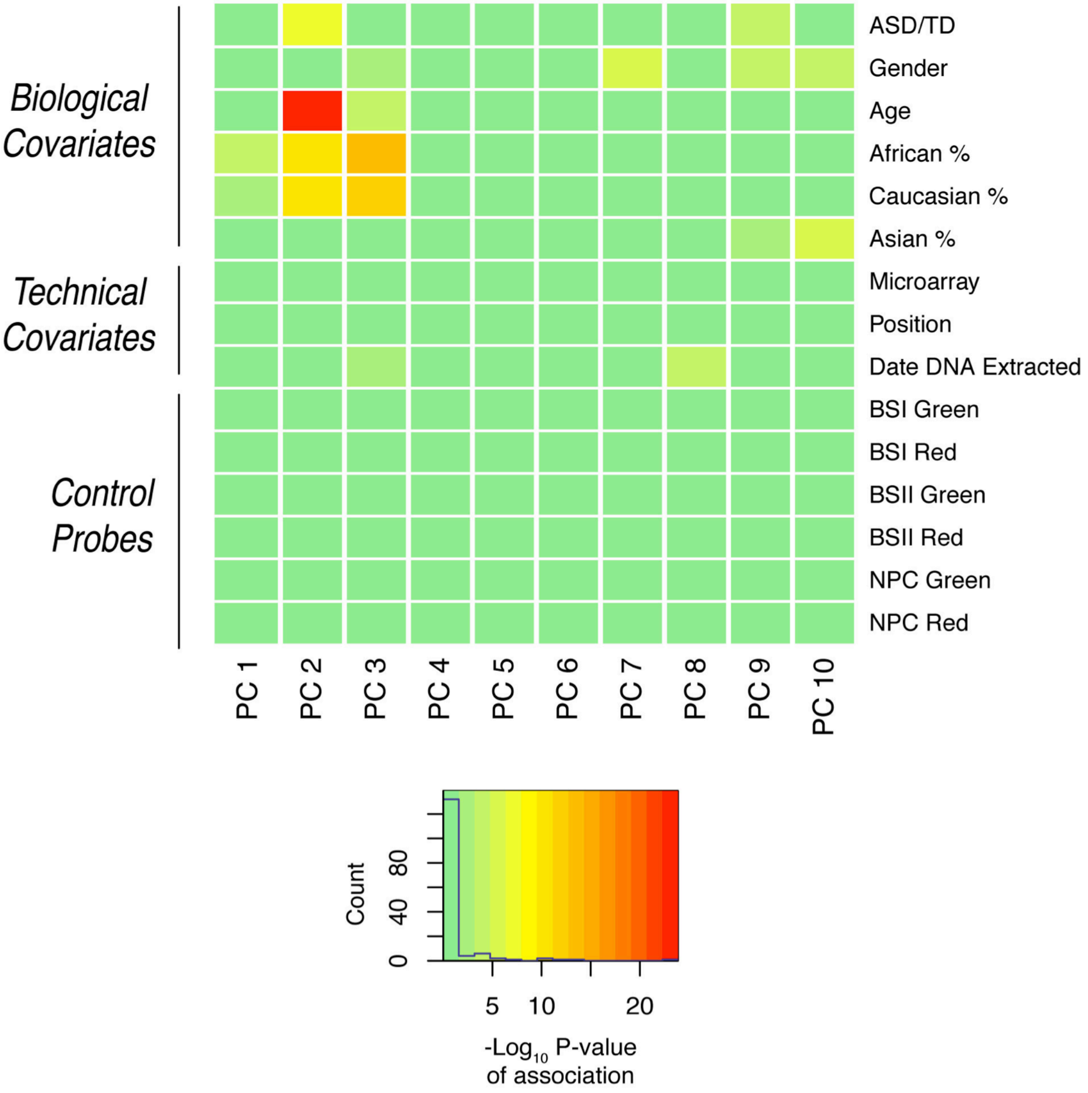

Supplemental Figure S4: Associations between principal components and known covariates

Heatmap of $-\log_{10}$ P-values for the association of each principal component with each known covariate demonstrates that variation due to technical artifact has been removed, while variation due to known biological covariates has been preserved for subsequent analysis.

The bump-hunting ASD-associated DMR output is provided in **Supplemental Table S4**. Stable DMRs are visualized in **Supplemental Figure S5.**

The differentially methylated regions were associated with the nearest overlapping gene, shown in **Table 1**. We investigated autism gene databases (Xu et al. 2012) and published literature for previous evidence linking the gene to ASD. We used UCSC genome browser (Kent et al. 2002) to determine the cytoband, and the SFARI gene CNV database (Basu et al. 2009) for a summary of CNV research implicating the cytoband in ASD. Gene functions were gleaned from UCSC genome browser and the GeneCards database (Rebhan et al. 1997).

chromosome	start	end	value	area	pns	indexStart	indexEnd	nprobes	avg	max	area.raw	gene name	Illumina annotation
1	248,100,183	248,100,614	0.427	4.273	23,221	45,365	45,374	10	0.080	0.150	0.803	*OR2L13*	1st exon, 5'UTR, TSS200 and TSS 1500, CpG island and north shore
2	241,290,446	241,291,070	0.381	1.904	137,222	274,789	274,793	5	0.086	0.104	0.428	*GPC1*	north shelf
2	114,033,360	114,033,830	0.331	1.323	128,333	258,706	258,709	4	0.075	0.105	0.301	*PAX8*	gene body, CpG island and north shore
4	3,748,154	3,748,554	0.465	2.324	163,348	326,311	326,315	5	0.119	0.148	0.595	*ADRA2C*	CpG island and north shore
5	139,227,979	139,228,242	-0.158	0.790	180,529	358,423	358,427	5	-0.076	-0.127	0.378	*NRG2*	gene body, CpG island
5	16,508,920	16,509,123	0.373	1.491	174,420	347,241	347,244	4	0.078	0.095	0.312	*FAM134B*	TSS200, 5'UTR, 1st exon, body, enhancer
6	73,329,988	73,330,358	-0.209	1.253	192,434	389,727	389,732	6	-0.077	-0.088	0.461	*KCNQ5*	TSS1500, CpG island north shore
7	28,452,066	28,452,289	-0.202	0.806	202,381	410,259	410,262	4	-0.076	-0.083	0.302	*CREB5*	TSS200, 5'UTR, 1st exon, CpG island south shelf
10	135,341,870	135,342,620	-0.491	2.947	35,126	69,284	69,289	6	-0.078	-0.148	0.469	*CYP2E1*	gene body, CG island and south shore
10	135,342,936	135,343,280	-0.462	1.850	35,127	69,290	69,293	4	-0.078	-0.098	0.310	*CYP2E1*	gene body, south shore
12	117,797,056	117,797,635	-0.213	1.067	57,778	115,485	115,489	5	-0.086	-0.109	0.432	*NOS1*	5'UTR, north shore
16	53,407,678	53,407,808	0.174	0.696	87,732	174,199	174,202	4	0.089	0.115	0.357	*LOC643802*	CpG island, south shore
16	2,879,944	2,880,326	-0.265	1.060	83,603	166,459	166,462	4	-0.101	-0.125	0.402	*ZG16B*	TSS200, TSS1500, 1st exon
16	1,796,832	1,797,383	-0.515	2.060	83,090	165,290	165,293	4	-0.090	-0.139	0.361	*MAPK8IP3*	gene body, CpG island and south shore
19	12,876,846	12,877,188	0.401	1.603	111,948	225,403	225,406	4	0.079	0.086	0.315	*HOOK2*	gene body, CpG island, north shore and south shore

Supplemental Table S4: DMRs Associated with ASD from Bump-Hunting.

Unstable DMRs are shaded in gray.

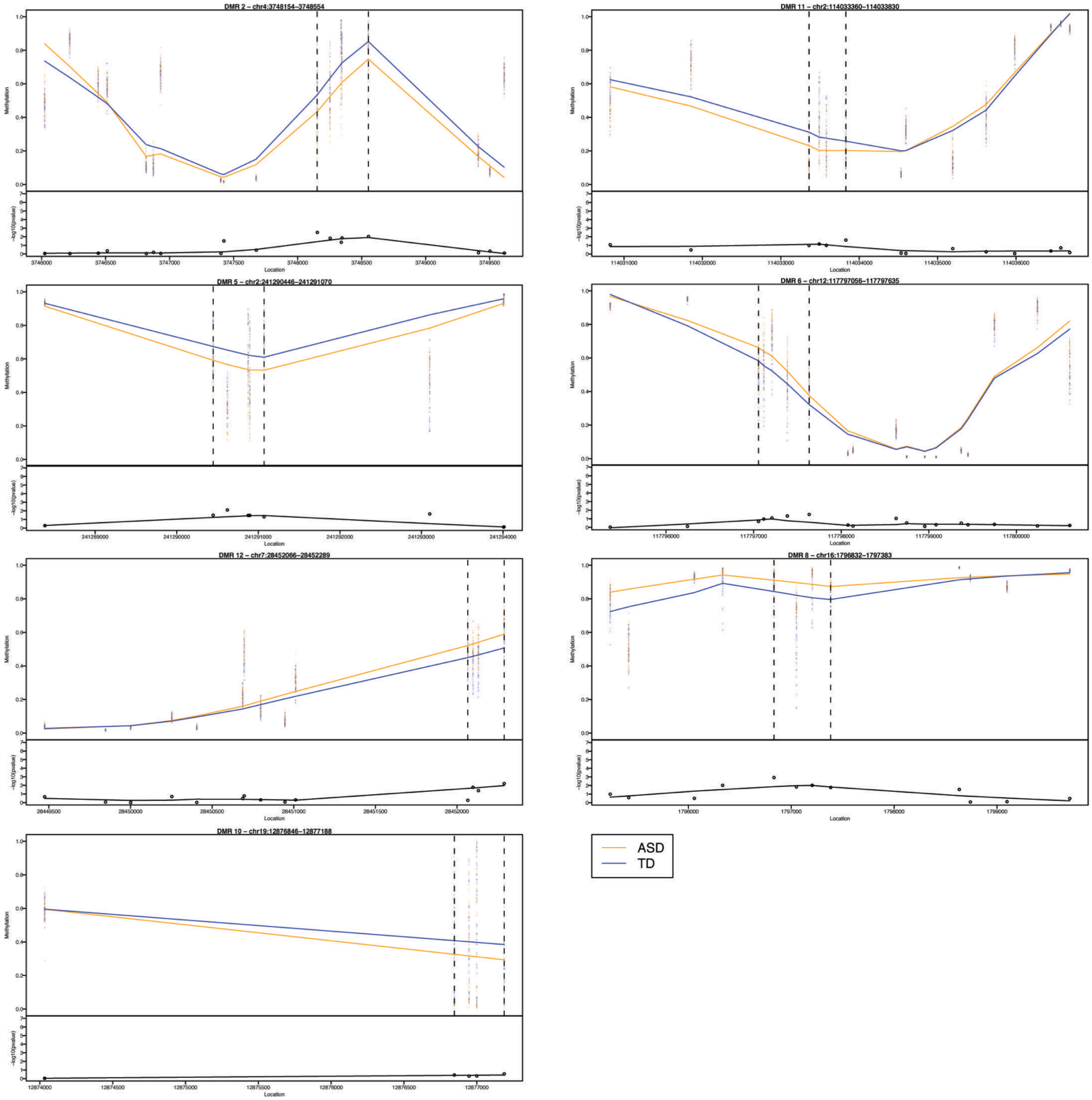

Supplemental Figure S5: Stable DMRs defined by the *bump-hunting* algorithm

DNA methylation values are displayed along with -log$_{10}$ p-values for each probe.

Left column, from top to bottom, gene names: *ADRA2C, GPC1, CREB5, HOOK2*.

Right column, top to bottom: *PAX8, NOS1, MAPK8IP*.

Copy number variant (CNV) Calling

The *CNVision* algorithm (Sanders et al. 2011) integrates three CNV-calling platforms: *GNOSIS*, *PennCNV*, and *QuantiSNP* (Colella et al. 2007), increasing accuracy of calls with this complementary assessment of each CNV. Since we wanted to exclude any loci with potential CNVs from methylation analysis, we retained all called CNVs, even those called with low potential probability.

When DMRs were found to overlap CNVs, we re-ran bump-hunting with these loci excluded to confirm that the local DMR was not solely due to the presence of CNVs in the region.

Massively-parallel bisulfite sequencing to validate differential DNA methylation

We bisulphite converted 500 ng of DNA using the Zymo EZ-96 Methylation-Lightning Kit. After separate PCR amplification of 4 target regions, we pooled the amplicons in averaged equal ratios and generated Illumina libraries using Tecan automation. Two sets of 48 libraries each were multiplexed on the MiSeq. Using bsmap (Bisulphite Sequencing Mapping Platform) (Xi and Li 2009) we checked for bisulphite conversion efficiency (C→T in CH contexts) and quantified the percent methylation for each person at every CpG in the amplicons.

We validated differential methylation for 3 predicted DMRs: the loci in *NOS1*, *FAM134B*, and *OR2L13*, and tested one of the loci with unstable DMR prediction (at the *KCNQ5* gene). The loci tested were chosen on the basis of their potential functional relevance and technical ability to design bisulphite PCR primers covering CpGs that overlapped with array probes. **Supplemental Table S5** shows the primers used.

Locus	Forward Primer	Reverse Primer
chr12:117797179-117797517	GGGGAAAAAATTTATGTTTTAGAGAG	AAAATTCTTCCCTCTACTCCCATAAC
chr6:73330181-73330482	GGTTTTTGTTGGTGATTAGGAGTAG	AAAAACAAAACTAAACTTCCACCAC
chr1:248100298-248100643	TTTTATTGTTTTTGGGGTTAATTAT	CACCAATATATAAAACAAAACCCTTC
chr5:16508806-16509201	TATTTTAATGTTGAATATTAGGAGGAAAA	AACCACTCCACCCTTAAATAAATAC

Supplemental Table S5: Primers for bisulphite-converted DNA

We showed a concordance of DNA methylation changes between microarray and sequencing-based approaches for all loci, but the degrees of difference of DNA methylation at the DMRs at *NOS1* and *KCNQ5* are insufficient to allow significant associations to be assigned statistically (**Supplemental Figure S6**).

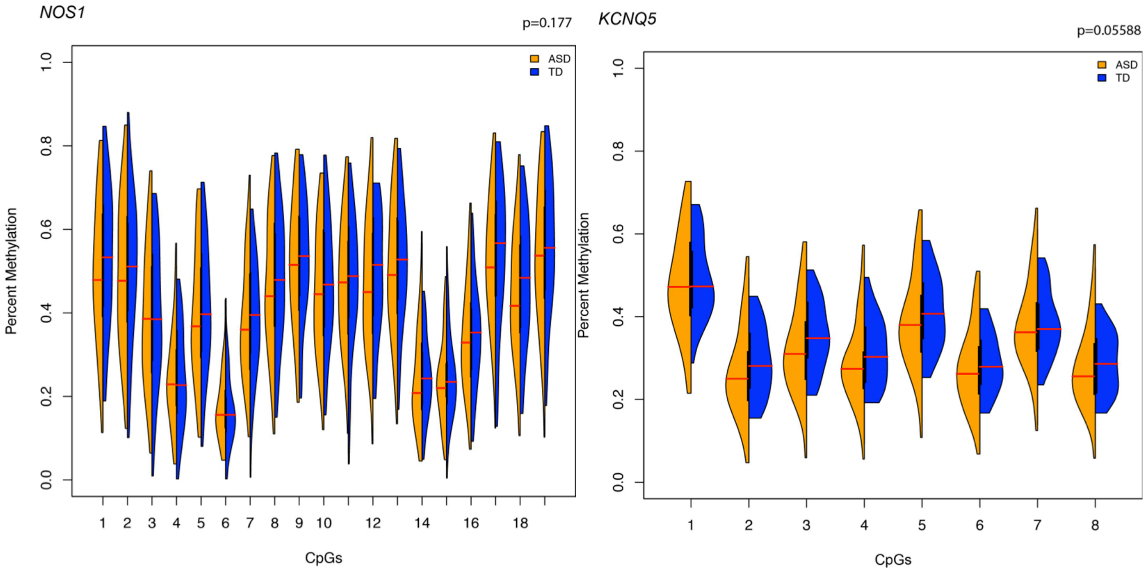

Supplemental Figure S6: Results of massively-parallel bisulphite sequencing of the *NOS1* and *KCNQ5* DMRs. The p values were calculated using t tests combining all of the loci tested in the putative DMR and comparing between the ASD and TD groups.

Gene Ontology Enrichment Analysis

For age-associated DMRs obtained from bump-hunting, we plotted only the enrichment categories with a threshold of the p-value with a significance of 10^{-7} or lower. Enrichment category results are displayed in **Supplemental Figure S7** and the DMRs themselves in **Supplemental Table S6**.

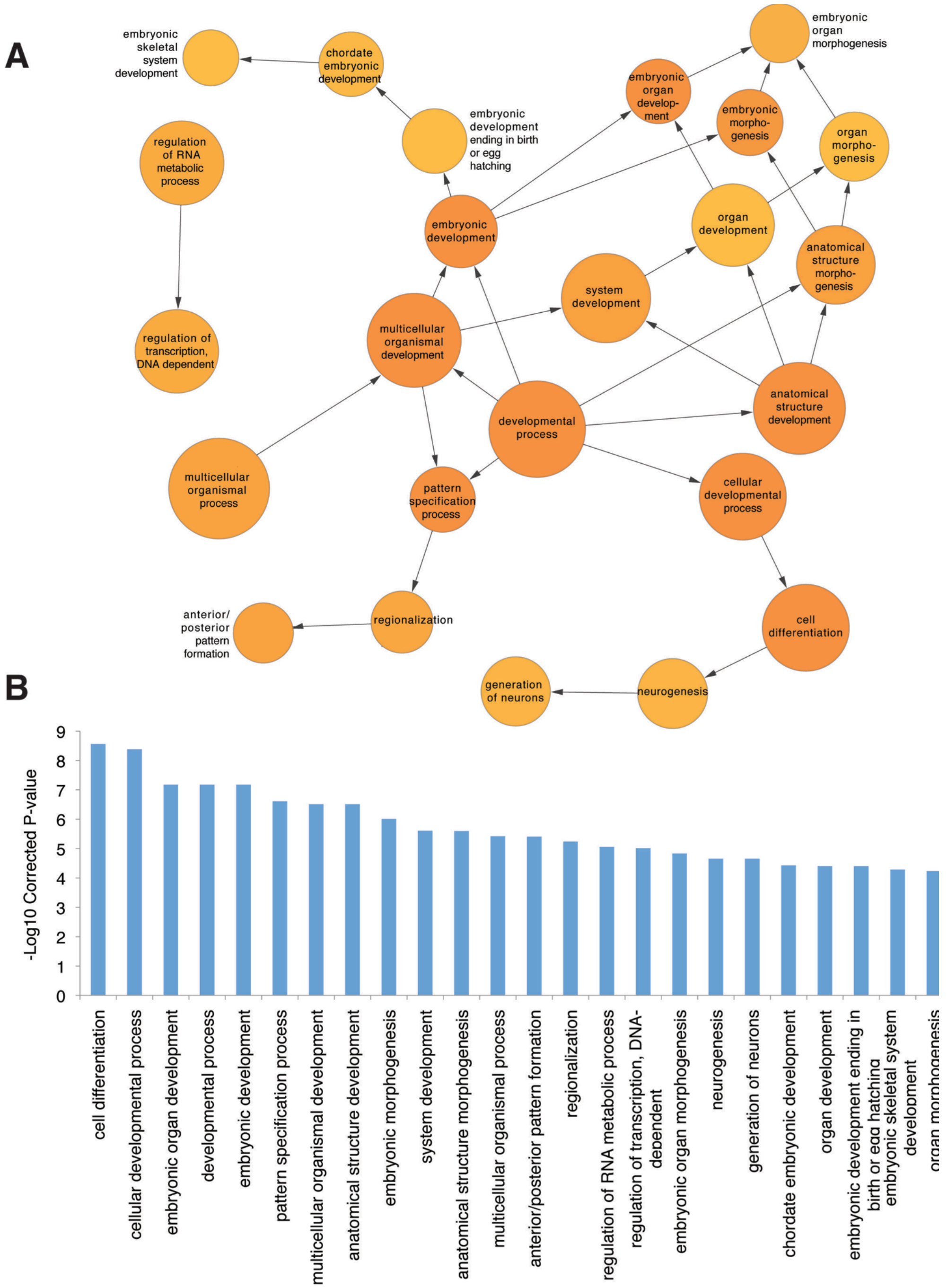

Supplemental Figure S7: Gene ontology analysis of genes associated with age-related DMRs.

Panel A shows connectivity of the gene ontology categories significantly enriched for genes related to age-associated DMRs, plotted by corrected p-value in **Panel B**.

Supplemental Table S6: Age-associated candidate DMRs.

chr	start	end	value	area	pns	indexStart	indexEnd	nprobes	avg	max	area.raw
**chr2**	176,986,460	176,987,465	0.029	0.318	132,157	265,348	265,358	11	0.732	0.855	8.054
**chr1**	231,155,632	231,156,204	-0.039	0.467	21,845	42,875	42,886	12	-0.609	-0.707	7.312
**chr6**	32,118,295	32,118,457	0.026	0.290	189,023	380,814	380,824	11	0.659	0.753	7.250
**chr19**	58,220,295	58,220,837	0.027	0.271	119,212	241,574	241,583	10	0.697	0.817	6.965
**chr14**	24,641,021	24,641,852	0.048	0.529	67,268	134,784	134,794	11	0.606	0.635	6.670
**chr8**	23,563,970	23,564,717	0.027	0.246	215,946	436,377	436,385	9	0.729	0.859	6.557
**chr1**	110,610,899	110,612,044	0.033	0.267	12,843	25,512	25,519	8	0.799	0.850	6.392
**chr19**	9,473,565	9,473,781	0.032	0.285	111,176	223,482	223,490	9	0.691	0.797	6.217
**chr16**	66,612,955	66,613,334	0.037	0.334	88,644	175,913	175,921	9	0.663	0.794	5.970
**chr10**	22,634,038	22,634,226	0.046	0.320	25,480	49,991	49,997	7	0.832	0.864	5.825
**chr13**	79,169,714	79,170,303	0.027	0.217	63,849	127,718	127,725	8	0.718	0.788	5.745
**chr6**	32,078,398	32,078,624	-0.028	0.225	189,003	380,630	380,637	8	-0.691	-0.752	5.525
**chr2**	177,029,459	177,030,171	0.027	0.192	132,199	265,502	265,508	7	0.775	0.798	5.425
**chr14**	29,234,890	29,235,196	0.029	0.229	67,445	135,202	135,209	8	0.666	0.827	5.332
**chr5**	87,441,081	87,441,969	0.031	0.249	177,623	353,048	353,055	8	0.659	0.812	5.272
**chr3**	147,126,638	147,127,097	0.035	0.244	158,563	316,905	316,911	7	0.752	0.852	5.264
**chr7**	27,205,200	27,205,658	0.025	0.223	202,198	409,826	409,834	9	0.583	0.696	5.251
**chr4**	155,661,691	155,662,795	0.029	0.259	170,611	339,758	339,766	9	0.556	0.630	5.004
**chr11**	86,383,182	86,383,430	0.041	0.286	44,708	90,004	90,010	7	0.707	0.738	4.950
**chr22**	24,890,690	24,890,833	0.043	0.301	145,700	292,479	292,485	7	0.678	0.766	4.744

**chr8**	143,858,414	143,858,636	0.036	0.255	223,217	449,399	449,405	7	0.669	0.736	4.680
**chr7**	99,775,422	99,775,558	0.034	0.240	207,428	419,896	419,902	7	0.666	0.745	4.661
**chr14**	57,275,967	57,276,789	0.034	0.204	68,821	137,800	137,805	6	0.773	0.871	4.638
**chr7**	27,225,058	27,225,143	0.029	0.172	202,219	409,908	409,913	6	0.772	0.848	4.631
**chr17**	7,832,680	7,833,237	0.028	0.255	94,610	188,539	188,547	9	0.511	0.575	4.602
**chr1**	248,020,436	248,020,812	0.043	0.258	23,207	45,341	45,346	6	0.749	0.833	4.492
**chr7**	8,481,994	8,482,614	0.037	0.225	201,229	407,774	407,779	6	0.747	0.836	4.481
**chr16**	87,864,324	87,865,062	-0.050	0.300	91,530	181,608	181,613	6	-0.721	-0.750	4.325
**chr1**	92,952,440	92,952,533	0.034	0.202	11,997	23,805	23,810	6	0.715	0.761	4.290
**chr2**	74,875,227	74,875,387	0.042	0.254	125,794	254,086	254,091	6	0.713	0.788	4.276
**chr8**	24,772,137	24,772,350	0.039	0.232	216,010	436,484	436,489	6	0.706	0.777	4.233
**chr14**	54,413,218	54,413,931	0.052	0.310	68,535	137,302	137,307	6	0.704	0.806	4.221
**chr10**	103,603,292	103,603,869	0.029	0.205	31,479	61,239	61,245	7	0.602	0.702	4.215
**chr1**	91,301,204	91,301,962	0.029	0.172	11,874	23,499	23,504	6	0.695	0.766	4.170
**chr3**	120,626,881	120,627,088	0.031	0.155	156,324	312,596	312,600	5	0.830	0.849	4.148
**chr2**	10,182,878	10,183,227	0.043	0.258	120,711	244,696	244,701	6	0.690	0.742	4.140
**chr12**	106,533,667	106,533,903	0.040	0.241	56,479	112,724	112,729	6	0.687	0.745	4.119
**chr11**	2,292,751	2,292,914	0.036	0.253	36,185	72,072	72,078	7	0.588	0.751	4.116
**chr6**	100,903,561	100,903,909	0.025	0.148	193,518	391,945	391,950	6	0.686	0.807	4.113
**chr6**	85,474,028	85,474,209	0.030	0.178	192,887	390,662	390,667	6	0.680	0.859	4.083
**chr11**	94,278,324	94,278,603	0.032	0.259	45,079	90,675	90,682	8	0.508	0.574	4.061
**chr5**	87,980,882	87,981,253	0.026	0.158	177,666	353,160	353,165	6	0.670	0.802	4.020
**chr2**	220,299,643	220,299,900	0.032	0.190	134,869	270,356	270,361	6	0.667	0.762	4.003

**chr22**	30,476,089	30,476,285	0.033	0.230	146,109	293,345	293,351	7	0.565	0.685	3.953
**chr5**	78,407,552	78,407,683	0.029	0.171	177,238	352,385	352,390	6	0.657	0.800	3.943
**chr6**	29,943,268	29,943,480	0.038	0.308	188,133	374,577	374,584	8	0.489	0.583	3.911
**chr1**	79,472,282	79,472,452	0.030	0.152	11,278	22,419	22,423	5	0.779	0.847	3.895
**chr18**	44,526,430	44,527,026	0.031	0.246	106,733	214,413	214,420	8	0.485	0.657	3.883
**chr13**	100,624,279	100,624,373	0.026	0.128	64,614	129,099	129,103	5	0.776	0.832	3.880
**chr5**	43,017,982	43,018,629	0.045	0.227	175,429	349,043	349,047	5	0.760	0.817	3.802
**chr4**	85,414,016	85,414,486	0.027	0.134	167,599	334,300	334,304	5	0.754	0.792	3.772
**chr22**	24,181,191	24,181,270	0.034	0.169	145,604	292,299	292,303	5	0.754	0.818	3.771
**chr5**	172,672,390	172,672,817	0.027	0.136	183,336	363,882	363,886	5	0.749	0.773	3.744
**chr1**	151,810,586	151,810,904	0.031	0.187	14,673	29,289	29,294	6	0.621	0.691	3.729
**chr3**	147,127,579	147,128,157	0.034	0.169	158,564	316,915	316,919	5	0.744	0.861	3.722
**chr7**	100,463,416	100,464,145	-0.059	0.353	207,617	420,346	420,351	6	-0.615	-0.639	3.692
**chr13**	79,170,627	79,171,230	0.028	0.139	63,849	127,727	127,731	5	0.737	0.838	3.683
**chr4**	85,402,870	85,403,409	0.027	0.137	167,592	334,286	334,290	5	0.732	0.761	3.658
**chr16**	86,547,203	86,547,544	0.027	0.137	91,139	180,813	180,817	5	0.731	0.767	3.654
**chr11**	86,085,623	86,086,005	0.026	0.158	44,685	89,972	89,977	6	0.609	0.731	3.652
**chr7**	19,146,032	19,146,555	0.025	0.123	201,554	408,365	408,369	5	0.730	0.799	3.650
**chr2**	223,164,831	223,164,925	0.027	0.134	135,087	270,748	270,752	5	0.727	0.805	3.637
**chr5**	176,827,082	176,827,793	0.040	0.237	183,904	364,948	364,953	6	0.599	0.723	3.597
**chr3**	147,125,712	147,125,782	0.031	0.157	158,563	316,888	316,892	5	0.717	0.828	3.586
**chr6**	85,482,570	85,483,055	0.032	0.159	192,892	390,680	390,684	5	0.715	0.793	3.573
**chr6**	11,044,877	11,044,974	0.067	0.269	186,057	369,233	369,236	4	0.892	0.905	3.568

**chr2**	176,948,693	176,948,759	0.029	0.147	132,115	265,209	265,213	5	0.706	0.784	3.528
**chr1**	91,190,366	91,190,891	0.028	0.139	11,865	23,457	23,461	5	0.705	0.795	3.527
**chr17**	46,685,292	46,685,448	0.028	0.141	99,899	199,582	199,586	5	0.701	0.744	3.505
**chr16**	1,593,152	1,593,766	-0.034	0.202	83,002	165,085	165,090	6	-0.583	-0.705	3.499
**chr16**	68,482,591	68,482,821	0.030	0.148	89,083	176,948	176,952	5	0.697	0.806	3.483
**chr11**	2,891,065	2,891,118	0.031	0.154	36,402	72,730	72,734	5	0.691	0.737	3.456
**chr16**	51,187,388	51,187,807	0.030	0.148	87,625	174,051	174,055	5	0.686	0.801	3.429
**chr10**	93,805,441	93,805,870	0.028	0.139	30,293	58,645	58,649	5	0.669	0.772	3.344
**chr17**	78,999,347	78,999,895	-0.032	0.162	104,247	208,702	208,706	5	-0.668	-0.739	3.342
**chr14**	100,069,535	100,069,840	0.055	0.222	72,269	144,115	144,118	4	0.827	0.851	3.307
**chr22**	40,417,285	40,417,869	-0.043	0.214	147,204	295,668	295,672	5	-0.653	-0.769	3.264
**chr12**	113,916,473	113,916,664	0.030	0.122	57,370	114,617	114,620	4	0.811	0.828	3.242
**chr8**	97,157,756	97,158,052	0.026	0.128	220,041	443,885	443,889	5	0.648	0.741	3.240
**chr1**	164,545,553	164,546,143	0.035	0.174	16,595	33,260	33,264	5	0.646	0.810	3.229
**chr3**	14,614,882	14,615,579	-0.032	0.161	149,961	301,002	301,006	5	-0.641	-0.692	3.203
**chr6**	101,846,779	101,846,872	0.026	0.130	193,554	392,047	392,051	5	0.638	0.692	3.191
**chr2**	176,964,506	176,964,720	0.030	0.118	132,130	265,271	265,274	4	0.798	0.830	3.190
**chr6**	73,329,988	73,330,358	0.036	0.217	192,434	389,727	389,732	6	0.529	0.610	3.176
**chr1**	119,535,693	119,535,986	0.038	0.153	13,653	27,185	27,188	4	0.794	0.872	3.175
**chr11**	123,066,529	123,067,275	0.030	0.181	47,241	94,847	94,852	6	0.529	0.683	3.174
**chr1**	228,400,217	228,400,693	0.034	0.136	21,459	42,125	42,128	4	0.788	0.822	3.153
**chr8**	56,015,399	56,015,785	0.026	0.105	218,073	440,284	440,287	4	0.784	0.808	3.135
**chr5**	170,288,742	170,289,070	0.034	0.171	183,006	363,218	363,222	5	0.625	0.864	3.127

**chr8**	25,898,191	25,898,539	0.031	0.126	216,106	436,652	436,655	4	0.778	0.803	3.111
**chr5**	126,626,348	126,626,364	0.025	0.099	179,378	356,130	356,133	4	0.777	0.826	3.106
**chr11**	134,147,143	134,147,634	0.034	0.136	48,559	97,232	97,235	4	0.773	0.855	3.091
**chr19**	17,958,339	17,958,736	0.032	0.159	113,027	227,806	227,810	5	0.618	0.744	3.091
**chr19**	57,182,844	57,183,268	0.031	0.124	119,037	241,073	241,076	4	0.768	0.814	3.070
**chr10**	28,035,631	28,035,894	0.038	0.267	25,811	50,667	50,673	7	0.439	0.487	3.070
**chr13**	79,177,877	79,177,925	0.027	0.107	63,854	127,748	127,751	4	0.759	0.790	3.037
**chr7**	101,005,910	101,006,089	0.031	0.215	207,774	420,718	420,724	7	0.433	0.521	3.032
**chr11**	64,146,487	64,146,822	-0.034	0.172	41,583	83,185	83,189	5	-0.606	-0.642	3.030
**chr7**	153,584,416	153,584,609	0.034	0.136	211,900	428,260	428,263	4	0.757	0.787	3.029
**chr1**	75,595,919	75,596,336	0.029	0.118	11,085	22,047	22,050	4	0.757	0.834	3.029
**chr8**	11,555,178	11,555,548	0.029	0.114	214,859	434,416	434,419	4	0.756	0.799	3.025
**chr12**	54,071,090	54,071,194	0.033	0.163	53,207	106,359	106,363	5	0.605	0.704	3.024
**chr1**	39,957,387	39,957,400	0.026	0.105	8,008	16,050	16,053	4	0.752	0.768	3.009
**chr5**	87,968,528	87,968,749	0.029	0.115	177,652	353,113	353,116	4	0.752	0.830	3.008
**chr10**	88,149,210	88,149,632	0.040	0.159	29,828	57,728	57,731	4	0.752	0.813	3.007
**chr1**	18,959,268	18,959,625	0.025	0.101	4,757	9,510	9,513	4	0.742	0.756	2.966
**chr2**	239,139,911	239,140,190	0.032	0.195	136,760	273,781	273,786	6	0.494	0.662	2.962
**chr16**	29,625,216	29,625,259	0.036	0.145	86,317	171,434	171,437	4	0.739	0.794	2.958
**chr3**	147,111,120	147,111,308	0.024	0.098	158,547	316,846	316,849	4	0.737	0.803	2.947
**chr11**	35,441,558	35,441,900	0.029	0.144	39,114	78,143	78,147	5	0.588	0.723	2.942
**chr2**	200,329,654	200,329,680	0.028	0.113	133,200	267,328	267,331	4	0.735	0.837	2.941
**chr5**	132,083,532	132,084,068	0.032	0.127	179,674	356,716	356,719	4	0.734	0.835	2.938

**chr8**	25,905,478	25,905,811	0.028	0.112	216,114	436,684	436,687	4	0.732	0.775	2.928
**chr7**	27,245,018	27,245,747	0.026	0.103	202,239	409,982	409,985	4	0.724	0.765	2.896
**chr1**	154,475,068	154,475,269	0.032	0.127	15,125	30,199	30,202	4	0.723	0.793	2.891
**chr18**	70,534,298	70,535,005	0.027	0.135	107,430	215,904	215,908	5	0.578	0.744	2.888
**chr5**	87,979,441	87,979,871	0.026	0.105	177,664	353,155	353,158	4	0.721	0.816	2.884
**chr14**	95,239,381	95,239,751	0.026	0.131	71,852	143,386	143,390	5	0.576	0.861	2.882
**chr11**	66,102,055	66,102,352	-0.029	0.144	42,230	84,801	84,805	5	-0.572	-0.642	2.861
**chr6**	100,054,585	100,054,817	0.026	0.104	193,460	391,824	391,827	4	0.714	0.855	2.855
**chr19**	2,046,085	2,046,350	-0.050	0.199	109,187	219,324	219,327	4	-0.711	-0.744	2.846
**chr6**	100,442,105	100,442,151	0.037	0.150	193,479	391,871	391,874	4	0.710	0.720	2.841
**chr18**	49,868,378	49,868,552	0.027	0.108	106,986	214,941	214,944	4	0.708	0.758	2.832
**chr3**	62,354,991	62,355,443	0.030	0.118	153,866	308,533	308,536	4	0.708	0.780	2.832
**chr4**	174,430,487	174,431,058	0.024	0.096	171,282	340,983	340,986	4	0.706	0.731	2.825
**chr3**	187,387,555	187,387,734	0.031	0.124	160,929	321,238	321,241	4	0.706	0.841	2.823
**chr10**	22,634,578	22,635,028	0.025	0.102	25,480	50,000	50,003	4	0.699	0.782	2.796
**chr6**	134,213,992	134,214,307	0.027	0.109	195,293	395,191	395,194	4	0.699	0.753	2.794
**chr17**	40,715,222	40,715,281	-0.026	0.132	98,728	196,894	196,898	5	-0.558	-0.644	2.788
**chr4**	52,942,997	52,943,247	0.042	0.169	166,053	331,543	331,546	4	0.696	0.757	2.784
**chr2**	223,161,771	223,162,128	0.032	0.127	135,085	270,727	270,730	4	0.695	0.792	2.780
**chr11**	111,385,450	111,385,659	0.027	0.109	45,879	92,155	92,158	4	0.695	0.805	2.779
**chr15**	60,288,082	60,288,404	0.026	0.103	77,285	154,102	154,105	4	0.694	0.759	2.777
**chr21**	36,041,605	36,041,699	0.033	0.134	143,100	287,541	287,544	4	0.694	0.716	2.775
**chr6**	156,718,177	156,718,546	0.039	0.193	196,828	397,938	397,942	5	0.554	0.626	2.770

**chr8**	98,290,310	98,290,372	0.034	0.137	220,135	444,065	444,068	4	0.692	0.826	2.767
**chr15**	58,357,922	58,357,989	0.028	0.110	77,129	153,822	153,825	4	0.691	0.723	2.764
**chr4**	48,486,087	48,486,472	0.026	0.106	165,951	331,335	331,338	4	0.687	0.752	2.748
**chr17**	36,719,518	36,719,937	0.034	0.172	97,771	194,856	194,860	5	0.549	0.726	2.744
**chr1**	19,665,070	19,665,240	0.040	0.161	4,926	9,817	9,820	4	0.686	0.731	2.744
**chr10**	126,136,228	126,136,709	0.059	0.237	33,326	64,892	64,895	4	0.683	0.755	2.733
**chr10**	50,604,330	50,604,569	0.024	0.097	27,342	53,347	53,350	4	0.680	0.779	2.721
**chr16**	2,563,274	2,563,560	0.024	0.097	83,480	166,190	166,193	4	0.679	0.803	2.717
**chr19**	10,397,612	10,397,780	0.031	0.125	111,331	223,899	223,902	4	0.678	0.766	2.713
**chr13**	37,004,721	37,004,812	0.025	0.127	61,968	124,283	124,287	5	0.542	0.570	2.711
**chr17**	61,778,366	61,778,813	0.025	0.100	101,437	202,761	202,764	4	0.675	0.730	2.701
**chr17**	58,216,297	58,216,651	0.030	0.119	101,114	202,101	202,104	4	0.672	0.755	2.688
**chr20**	55,964,998	55,965,497	0.035	0.138	141,209	283,686	283,689	4	0.671	0.781	2.682
**chr2**	157,176,971	157,177,345	0.035	0.141	130,820	262,911	262,914	4	0.671	0.825	2.682
**chr8**	143,545,478	143,545,949	0.034	0.170	223,102	449,175	449,179	5	0.536	0.619	2.680
**chr8**	1,273,592	1,273,856	0.028	0.139	213,656	432,090	432,094	5	0.535	0.646	2.674
**chr12**	66,627,900	66,628,232	-0.025	0.099	54,519	109,239	109,242	4	-0.667	-0.715	2.668
**chr14**	29,243,404	29,243,690	0.028	0.114	67,454	135,237	135,240	4	0.666	0.790	2.665
**chr1**	41,119,634	41,119,988	0.030	0.121	8,190	16,458	16,461	4	0.660	0.741	2.638
**chr6**	30,653,512	30,653,659	-0.028	0.112	188,431	376,470	376,473	4	-0.659	-0.679	2.635
**chr4**	151,500,631	151,501,298	0.037	0.148	170,283	339,171	339,174	4	0.659	0.774	2.634
**chr16**	85,932,591	85,932,853	0.031	0.123	90,989	180,519	180,522	4	0.658	0.752	2.632
**chr2**	177,053,274	177,053,292	0.028	0.113	132,211	265,539	265,542	4	0.658	0.724	2.631

**chr13**	24,844,846	24,844,938	0.028	0.168	60,981	122,394	122,399	6	0.438	0.541	2.631
**chr12**	56,414,442	56,414,533	0.025	0.099	53,637	107,388	107,391	4	0.654	0.701	2.616
**chr11**	46,383,031	46,383,209	0.028	0.111	39,798	79,438	79,441	4	0.653	0.724	2.611
**chr19**	18,260,330	18,260,515	-0.033	0.131	113,106	227,997	228,000	4	-0.650	-0.685	2.599
**chr12**	126,675,667	126,676,048	0.026	0.103	59,051	118,197	118,200	4	0.650	0.770	2.598
**chr6**	110,736,772	110,737,053	-0.033	0.163	194,095	393,045	393,049	5	-0.517	-0.584	2.586
**chr15**	67,356,310	67,356,942	0.035	0.174	78,156	155,651	155,655	5	0.516	0.559	2.579
**chr8**	61,777,711	61,778,137	-0.036	0.146	218,407	440,878	440,881	4	-0.643	-0.724	2.572
**chr1**	6,515,580	6,515,748	0.036	0.145	2,387	5,290	5,293	4	0.643	0.703	2.570
**chr11**	17,803,160	17,803,421	-0.036	0.145	38,057	76,022	76,025	4	-0.642	-0.684	2.567
**chr17**	46,618,919	46,619,555	0.026	0.104	99,835	199,378	199,381	4	0.641	0.826	2.565
**chr10**	99,734,416	99,734,912	0.034	0.169	30,914	59,888	59,892	5	0.513	0.616	2.564
**chr4**	175,132,842	175,133,151	0.024	0.096	171,327	341,091	341,094	4	0.640	0.744	2.561
**chr1**	200,009,830	200,010,283	0.024	0.097	18,525	36,715	36,718	4	0.638	0.685	2.551
**chr5**	54,518,745	54,519,159	0.028	0.113	175,795	349,774	349,777	4	0.637	0.754	2.549
**chr6**	30,070,059	30,070,403	0.038	0.339	188,216	375,015	375,023	9	0.283	0.533	2.543
**chr20**	33,762,474	33,762,943	0.028	0.111	139,752	280,319	280,322	4	0.635	0.727	2.540
**chr7**	94,284,865	94,284,900	0.030	0.121	206,839	418,369	418,372	4	0.634	0.835	2.535
**chr2**	175,208,588	175,208,761	0.026	0.103	131,985	264,936	264,939	4	0.631	0.743	2.526
**chr14**	94,392,718	94,392,932	-0.030	0.121	71,715	143,118	143,121	4	-0.630	-0.671	2.519
**chr12**	50,426,531	50,427,095	0.032	0.127	52,527	104,971	104,974	4	0.628	0.698	2.512
**chr1**	17,085,860	17,086,071	0.030	0.121	4,431	8,941	8,944	4	0.627	0.684	2.508
**chr17**	27,038,861	27,039,058	0.028	0.110	96,631	192,556	192,559	4	0.627	0.717	2.506

**chr12**	11,653,278	11,653,827	0.024	0.098	50,245	100,630	100,633	4	0.626	0.842	2.505
**chr21**	36,399,146	36,399,540	0.041	0.166	143,128	287,597	287,600	4	0.625	0.684	2.498
**chr7**	142,494,148	142,494,244	0.028	0.113	210,754	426,012	426,015	4	0.622	0.724	2.490
**chr2**	74,663,416	74,663,698	0.026	0.104	125,719	253,871	253,874	4	0.622	0.725	2.487
**chr1**	91,300,288	91,300,446	0.025	0.102	11,873	23,494	23,497	4	0.622	0.653	2.486
**chr17**	42,733,527	42,733,600	0.033	0.130	99,202	198,076	198,079	4	0.611	0.835	2.443
**chr10**	110,225,900	110,226,387	0.025	0.099	31,992	62,298	62,301	4	0.611	0.627	2.443
**chr1**	3,473,665	3,474,376	-0.028	0.111	1,736	3,933	3,936	4	-0.610	-0.781	2.440
**chr13**	28,545,214	28,545,566	0.025	0.098	61,363	123,182	123,185	4	0.607	0.697	2.429
**chr3**	194,408,516	194,408,901	0.036	0.143	161,474	322,164	322,167	4	0.607	0.695	2.427
**chr1**	33,231,272	33,231,382	0.035	0.140	7,123	14,255	14,258	4	0.603	0.623	2.412
**chr17**	1,881,005	1,881,333	0.036	0.145	93,375	185,564	185,567	4	0.601	0.611	2.404
**chr1**	115,881,130	115,881,259	0.029	0.114	13,375	26,633	26,636	4	0.601	0.698	2.403
**chr4**	109,093,158	109,093,243	0.025	0.101	168,519	336,010	336,013	4	0.595	0.663	2.380
**chr2**	186,603,398	186,603,639	0.030	0.120	132,626	266,293	266,296	4	0.592	0.691	2.369
**chr2**	220,196,530	220,196,755	0.031	0.125	134,841	270,288	270,291	4	0.592	0.800	2.368
**chr7**	101,512,529	101,513,100	0.025	0.099	207,835	420,826	420,829	4	0.592	0.650	2.367
**chr13**	112,709,256	112,709,550	0.024	0.097	65,560	130,940	130,943	4	0.590	0.803	2.361
**chr10**	94,451,351	94,451,736	0.026	0.105	30,344	58,759	58,762	4	0.589	0.644	2.358
**chr6**	29,521,781	29,521,803	0.030	0.121	187,956	373,759	373,762	4	0.588	0.632	2.352
**chr17**	4,648,566	4,648,949	0.034	0.135	93,932	186,796	186,799	4	0.586	0.755	2.346
**chr19**	11,529,947	11,530,065	0.029	0.145	111,665	224,649	224,653	5	0.468	0.552	2.342
**chr8**	10,261,972	10,262,221	0.029	0.117	214,638	433,984	433,987	4	0.585	0.697	2.338

**chr11**	125,036,088	125,036,420	0.030	0.119	47,523	95,377	95,380	4	0.584	0.668	2.334
**chr1**	4,770,676	4,771,201	0.034	0.136	2,015	4,574	4,577	4	0.583	0.707	2.333
**chr12**	58,003,774	58,003,965	0.025	0.127	54,003	108,274	108,278	5	0.466	0.629	2.331
**chr11**	9,025,730	9,026,308	0.035	0.140	37,246	74,531	74,534	4	0.580	0.758	2.322
**chr11**	334,298	334,833	0.035	0.138	35,235	69,532	69,535	4	0.576	0.582	2.305
**chr17**	7,350,001	7,350,413	0.026	0.103	94,440	188,076	188,079	4	0.574	0.627	2.297
**chr1**	47,882,686	47,883,234	0.025	0.102	9,213	18,622	18,625	4	0.574	0.790	2.295
**chr10**	8,097,331	8,097,689	0.032	0.128	24,556	48,236	48,239	4	0.573	0.672	2.293
**chr4**	41,646,293	41,646,672	0.026	0.105	165,708	330,788	330,791	4	0.572	0.657	2.290
**chr13**	112,717,207	112,717,707	0.031	0.126	65,571	130,968	130,971	4	0.571	0.630	2.284
**chr1**	228,246,632	228,247,135	0.027	0.108	21,403	41,970	41,973	4	0.570	0.623	2.282
**chr22**	17,083,412	17,083,727	0.025	0.101	144,702	290,507	290,510	4	0.570	0.658	2.280
**chr8**	145,106,246	145,106,582	0.039	0.155	223,787	450,753	450,756	4	0.568	0.584	2.272
**chr6**	125,855,124	125,855,421	0.027	0.110	194,820	394,326	394,329	4	0.565	0.583	2.261
**chr6**	170,597,326	170,597,588	-0.036	0.145	198,758	401,914	401,917	4	-0.562	-0.663	2.248
**chr5**	172,110,211	172,110,579	0.028	0.113	183,213	363,608	363,611	4	0.559	0.695	2.237
**chr11**	1,483,731	1,483,973	0.026	0.103	35,852	71,128	71,131	4	0.559	0.663	2.236
**chr12**	113,913,695	113,914,222	0.028	0.112	57,369	114,609	114,612	4	0.557	0.748	2.229
**chr1**	236,557,182	236,557,682	-0.032	0.127	22,345	43,778	43,781	4	-0.557	-0.739	2.229
**chr3**	100,712,058	100,712,345	-0.024	0.097	155,320	310,850	310,853	4	-0.555	-0.560	2.221
**chr15**	45,996,521	45,996,787	0.028	0.112	76,437	152,529	152,532	4	0.553	0.665	2.213
**chr15**	37,387,304	37,387,577	0.032	0.160	75,453	150,422	150,426	5	0.443	0.507	2.213
**chr22**	19,748,777	19,749,188	0.032	0.126	145,030	291,203	291,206	4	0.552	0.710	2.208

**chr19**	3,097,565	3,097,728	-0.028	0.111	109,601	220,187	220,190	4	-0.549	-0.559	2.197
**chr16**	66,400,320	66,400,411	-0.032	0.126	88,601	175,821	175,824	4	-0.549	-0.699	2.196
**chr1**	228,225,533	228,225,687	0.030	0.119	21,398	41,960	41,963	4	0.549	0.571	2.194
**chr19**	15,530,606	15,530,870	-0.025	0.100	112,528	226,742	226,745	4	-0.547	-0.653	2.188
**chr3**	42,307,519	42,307,866	0.024	0.097	151,540	303,761	303,764	4	0.544	0.672	2.175
**chr1**	159,825,552	159,825,761	0.028	0.112	16,043	32,151	32,154	4	0.540	0.740	2.160
**chr8**	54,569,668	54,570,293	0.031	0.124	217,968	440,066	440,069	4	0.536	0.569	2.143
**chr5**	493,262	493,746	0.036	0.182	172,643	343,608	343,612	5	0.424	0.485	2.121
**chr6**	29,795,501	29,795,595	0.034	0.136	188,094	374,395	374,398	4	0.525	0.586	2.101
**chr17**	80,289,500	80,289,701	-0.027	0.107	104,929	210,429	210,432	4	-0.520	-0.603	2.081
**chr10**	70,321,770	70,321,959	0.031	0.124	28,175	54,833	54,836	4	0.519	0.636	2.075
**chr12**	117,797,056	117,797,635	0.040	0.200	57,778	115,485	115,489	5	0.413	0.545	2.064
**chr19**	11,353,961	11,354,240	0.028	0.113	111,615	224,525	224,528	4	0.514	0.652	2.055
**chr2**	26,624,760	26,624,865	0.025	0.101	121,960	246,995	246,998	4	0.512	0.579	2.048
**chr16**	56,696,748	56,697,229	0.028	0.111	88,038	174,840	174,843	4	0.511	0.680	2.045
**chr4**	140,656,749	140,657,110	0.026	0.103	169,748	338,243	338,246	4	0.510	0.631	2.040
**chr19**	1,387,394	1,387,894	-0.028	0.110	108,823	218,581	218,584	4	-0.509	-0.687	2.036
**chr14**	102,554,826	102,554,977	0.033	0.132	72,843	145,414	145,417	4	0.509	0.647	2.035
**chr6**	10,883,895	10,884,314	0.033	0.130	186,038	369,196	369,199	4	0.508	0.553	2.034
**chr17**	56,565,286	56,565,644	0.027	0.109	100,960	201,742	201,745	4	0.506	0.511	2.025
**chr20**	44,746,392	44,747,006	-0.028	0.285	140,615	282,425	282,434	10	-0.202	-0.249	2.018
**chr6**	146,755,301	146,755,900	0.027	0.110	196,115	396,635	396,638	4	0.500	0.767	1.999
**chr1**	11,708,792	11,709,271	0.033	0.131	3,510	7,279	7,282	4	0.496	0.660	1.983

**chr11**	82,443,149	82,443,614	0.028	0.113	44,506	89,617	89,620	4	0.496	0.675	1.982
**chr1**	17,215,834	17,216,201	0.037	0.147	4,452	8,979	8,982	4	0.494	0.646	1.975
**chr2**	233,251,770	233,252,170	0.041	0.166	135,805	272,015	272,018	4	0.483	0.516	1.933
**chr1**	23,884,703	23,885,086	0.035	0.139	5,630	11,094	11,097	4	0.482	0.511	1.930
**chr8**	143,763,326	143,763,565	-0.028	0.110	223,182	449,333	449,336	4	-0.482	-0.511	1.927
**chr8**	145,008,957	145,009,406	-0.030	0.118	223,731	450,584	450,587	4	-0.481	-0.548	1.925
**chr1**	7,842,159	7,842,406	-0.033	0.132	2,675	5,804	5,807	4	-0.480	-0.577	1.919
**chr20**	61,732,467	61,732,608	0.027	0.106	141,955	285,308	285,311	4	0.476	0.561	1.904
**chr22**	51,016,501	51,016,644	0.025	0.100	148,747	298,687	298,690	4	0.471	0.574	1.885
**chr19**	52,996,083	52,996,617	0.029	0.117	118,026	238,929	238,932	4	0.469	0.534	1.877
**chr1**	227,748,424	227,748,719	0.030	0.120	21,322	41,802	41,805	4	0.466	0.588	1.863
**chr3**	50,487,955	50,488,230	0.025	0.098	152,798	306,618	306,621	4	0.464	0.620	1.856
**chr13**	112,712,424	112,712,795	0.025	0.101	65,563	130,953	130,956	4	0.458	0.570	1.832
**chr1**	228,346,014	228,346,347	0.028	0.112	21,442	42,079	42,082	4	0.458	0.540	1.830
**chr19**	19,639,553	19,639,596	0.033	0.131	113,496	228,856	228,859	4	0.455	0.615	1.819
**chr22**	46,449,498	46,449,821	-0.031	0.124	147,948	297,262	297,265	4	-0.453	-0.486	1.811
**chr2**	114,033,360	114,033,830	0.028	0.112	128,333	258,706	258,709	4	0.448	0.454	1.792
**chr20**	5,485,144	5,485,294	-0.030	0.181	138,494	277,554	277,559	6	-0.298	-0.320	1.787
**chr8**	56,791,576	56,791,798	-0.033	0.132	218,114	440,360	440,363	4	-0.446	-0.476	1.785
**chr6**	31,148,404	31,148,483	-0.025	0.124	188,606	377,487	377,491	5	-0.349	-0.373	1.747
**chr13**	112,187,145	112,187,396	0.030	0.121	65,453	130,675	130,678	4	0.419	0.484	1.676
**chr17**	73,584,029	73,584,111	0.028	0.141	102,695	205,201	205,205	5	0.335	0.522	1.673
**chr20**	44,803,246	44,803,686	0.032	0.128	140,625	282,446	282,449	4	0.416	0.476	1.663

**chr15**	81,410,745	81,411,066	0.031	0.126	79,970	158,935	158,938	4	0.416	0.507	1.662
**chr7**	27,138,712	27,138,974	0.026	0.105	202,131	409,513	409,516	4	0.415	0.493	1.661
**chr6**	32,055,135	32,055,316	0.027	0.107	188,977	380,461	380,464	4	0.411	0.627	1.643
**chr12**	6,486,598	6,486,709	-0.024	0.098	49,523	99,109	99,112	4	-0.411	-0.567	1.643
**chr12**	53,358,946	53,359,506	0.030	0.118	53,044	105,984	105,987	4	0.410	0.466	1.640
**chr12**	132,939,657	132,939,992	0.028	0.114	60,038	120,334	120,337	4	0.409	0.455	1.638
**chr2**	66,659,348	66,659,590	0.025	0.098	124,840	252,149	252,152	4	0.404	0.514	1.615
**chr16**	54,321,848	54,322,494	0.027	0.108	87,801	174,341	174,344	4	0.401	0.574	1.602
**chr17**	43,716,423	43,716,617	-0.024	0.098	99,443	198,608	198,611	4	-0.393	-0.451	1.570
**chr5**	496,069	496,476	0.035	0.141	172,645	343,618	343,621	4	0.392	0.628	1.568
**chr9**	96,715,687	96,716,209	0.025	0.101	226,639	455,043	455,046	4	0.386	0.424	1.543
**chr4**	80,885,735	80,886,264	0.024	0.097	167,324	333,738	333,741	4	0.378	0.548	1.514
**chr6**	37,616,410	37,616,803	0.027	0.107	190,315	385,855	385,858	4	0.378	0.556	1.512
**chr16**	4,103,167	4,103,533	-0.026	0.103	84,029	167,363	167,366	4	-0.372	-0.470	1.486
**chr16**	55,794,456	55,794,910	0.033	0.164	87,927	174,589	174,593	5	0.272	0.363	1.361
**chr9**	34,370,781	34,370,894	0.025	0.099	224,994	452,784	452,787	4	0.336	0.491	1.345
**chr22**	26,875,499	26,875,652	-0.025	0.102	145,844	292,777	292,780	4	-0.336	-0.374	1.342
**chr4**	940,614	941,054	0.029	0.115	162,328	323,947	323,950	4	0.333	0.414	1.330
**chr7**	4,901,337	4,901,628	0.026	0.105	200,602	406,489	406,492	4	0.330	0.366	1.318
**chr17**	81,045,495	81,045,863	0.025	0.101	105,350	211,445	211,448	4	0.320	0.590	1.281
**chr6**	292,329	292,823	-0.031	0.157	184,828	366,824	366,828	5	-0.250	-0.260	1.252
**chr7**	4,832,112	4,832,359	0.030	0.119	200,565	406,401	406,404	4	0.309	0.356	1.236
**chr5**	139,227,979	139,228,242	0.038	0.192	180,529	358,423	358,427	5	0.247	0.311	1.236

**chr11**	2,406,712	2,407,267	-0.027	0.109	36,227	72,232	72,235	4	-0.292	-0.551	1.168
**chr11**	6,592,066	6,592,745	0.024	0.096	36,931	73,912	73,915	4	0.289	0.321	1.157
**chr5**	664,363	664,666	-0.035	0.139	172,747	343,836	343,839	4	-0.287	-0.317	1.147
**chr3**	194,705,841	194,706,168	-0.030	0.121	161,493	322,192	322,195	4	-0.274	-0.334	1.096
**chr21**	46,975,805	46,976,340	0.027	0.110	144,394	290,012	290,015	4	0.264	0.404	1.055
**chr6**	31,237,029	31,237,405	0.045	0.179	188,622	377,561	377,564	4	0.232	0.238	0.928
**chr19**	55,477,653	55,477,810	-0.030	0.120	118,579	240,113	240,116	4	-0.164	-0.198	0.655
**chr2**	128,453,108	128,453,484	0.034	0.171	129,225	260,338	260,342	5	0.114	0.157	0.571
**chr4**	6,728,936	6,729,199	0.035	0.141	163,790	327,281	327,284	4	0.128	0.205	0.513
**chr12**	131,622,284	131,622,739	-0.028	0.111	59,596	119,302	119,305	4	-0.085	-0.163	0.340

The table shows the results of the *dmrFind* algorithm. DMR positions are shown in the chr/start/end co-ordinates, with probe indices and numbers represented by indexStart, indexEnd and nprobes, and area_raw the significance calculation following permutation analysis, allowing ranking of these DMRs by significance, as shown.

Weighted Gene Co-expression Network Analysis (WGCNA)

We performed weighted gene co-expression network analysis (WGCNA) (Langfelder and Horvath 2008) to assess networks of co-methylated CpGs associated with ASD status. We used the CpG values output from the surrogate variable analysis (SVA) algorithm called in bump-hunting, since SVA corrected for all known technical artifacts. We used the WGCNA package in R and built an unsigned co-methylation network. Correlation matrices were raised to the power of 5, as calculated by the scale-free topology criterion on data subsets, and thresholds were set of minimum module connectivity (kME) of greater than 0.7, and minimum height for module merging of 0.1 (Voineagu et al. 2011). We ran the algorithm with block sizes of 40,000 CpGs.

We assessed module relevance to case/control status with a t-test (two tailed, unequal variance) of module eigengene values with case/control and gender categories. For relationship with the continuous variables of age and percent YRI and CEU ancestry, we used Pearson correlation coefficients and their Student asymptotic p-values.

For analysis of methylation changes associated with ASD, we selected the 2 modules (“light green”, and “dark olive green2”) that showed significant correlation only with ASD status and not with any other covariate, to avoid confounding effects.

A full list of the modules obtained and their Bonferroni-corrected p-values is provided in **Supplemental Table S7**.

The genes associated with “light green” and “dark olive green 2” two ASD-associated modules are listed in **Supplemental Tables S8 and S9**.

Gene ontology enrichment was performed using the same Cytoscape method described previously. For the light green module, gene ontology showed significant enrichment for negative regulation of smooth muscle cell migration ($p = 1.34 \times 10^{-2}$), regulation of cell proliferation ($p = 3.25 \times 10^{-2}$), negative regulation of cell migration ($p = 3.27 \times 10^{-2}$), negative regulation of locomotion ($p = 3.27 \times 10^{-2}$), negative regulation of cellular component movement ($p = 3.27 \times 10^{-2}$), and negative regulation of metabolic process ($p = 4.64 \times 10^{-2}$). All p-values shown are after FDR correction.

It has been recently demonstrated that gene ontology analysis can be affected by unequal probe distribution in microarrays, with bias towards genes represented by greater numbers of probes (Geeleher et al. 2013). To address this concern, we calculated the number of probes corresponding to every gene by using annotations assigned by Illumina in the 450K array manifest. We used the number of probes per gene to generate a weight for every gene and recalculated enriched gene ontologies, using the R package *GoSeq* (Young et al. 2010). After accounting for unequal probe distribution, we obtained candidate ontology categories, but these did not remain significant after correcting the associated p-values for multiple testing.

We also interrogated some of the modules most significantly associated with age and ancestry, the “dark turquoise” and “red” modules, respectively. Using the *Lists2Networks* software (Lachmann and Ma'ayan 2010), we found that the most significant gene ontology category enriched with genes from the age module was brain development (p=0.008). We then asked whether the ancestry-associated modules were enriched for CpGs with annotated SNPs on either the C or G. Out of 247 CpGs, 154 contained a reported variant (based on 1000 Genomes data), with 11 of those possessing variants on both the C and the G. This confirms the

importance of both polymorphism on the CpG, as well as the presence of ancestry-associated methylation changes resulting from effects other than direct polymorphism.

Protein-Protein Interaction (PPI) Analysis

To assess the functional impact of our WGCNA ASD-associated co-methylated gene modules, we interrogated the genes' relevance in protein-protein interaction (PPI) networks. We combined the genes in the light green and dark olive green 2 modules with a list of previously curated known ASD risk genes, with the addition of exome sequencing candidates (*KATNAL2* and *CHD8*) (Neale et al. 2012). We used GeneMania (Warde-Farley et al. 2010) to build a PPI network of this combined list, using only data from physical protein interaction databases. We visualized this network in Cytoscape.

To test the significance of these PPI connections, we performed Degree Aware Disease Gene Prioritization (DADA) (Erten et al. 2011). We used the ASD seed list mentioned previously, a combined candidate list of the genes related to the ASD-associated WGCNA CpGs, and the physical interaction database from the Human Protein Reference Database (HPRD) available through GeneMania. We evaluated if our genes were significantly enriched in ranking using the Mann-Whitney test.

We also assessed whether the candidate genes related to ASD-associated CpG modules were functionally connected to known risk genes for Intellectual Disability (ID). We used a previously curated list of ID-implicated genes (Neale et al. 2012) and combined that with our 2 module genes to build a PPI network. Using the same parameters as above, we generated a network showing many functional connections, displayed in **Supplemental Figure S8**.

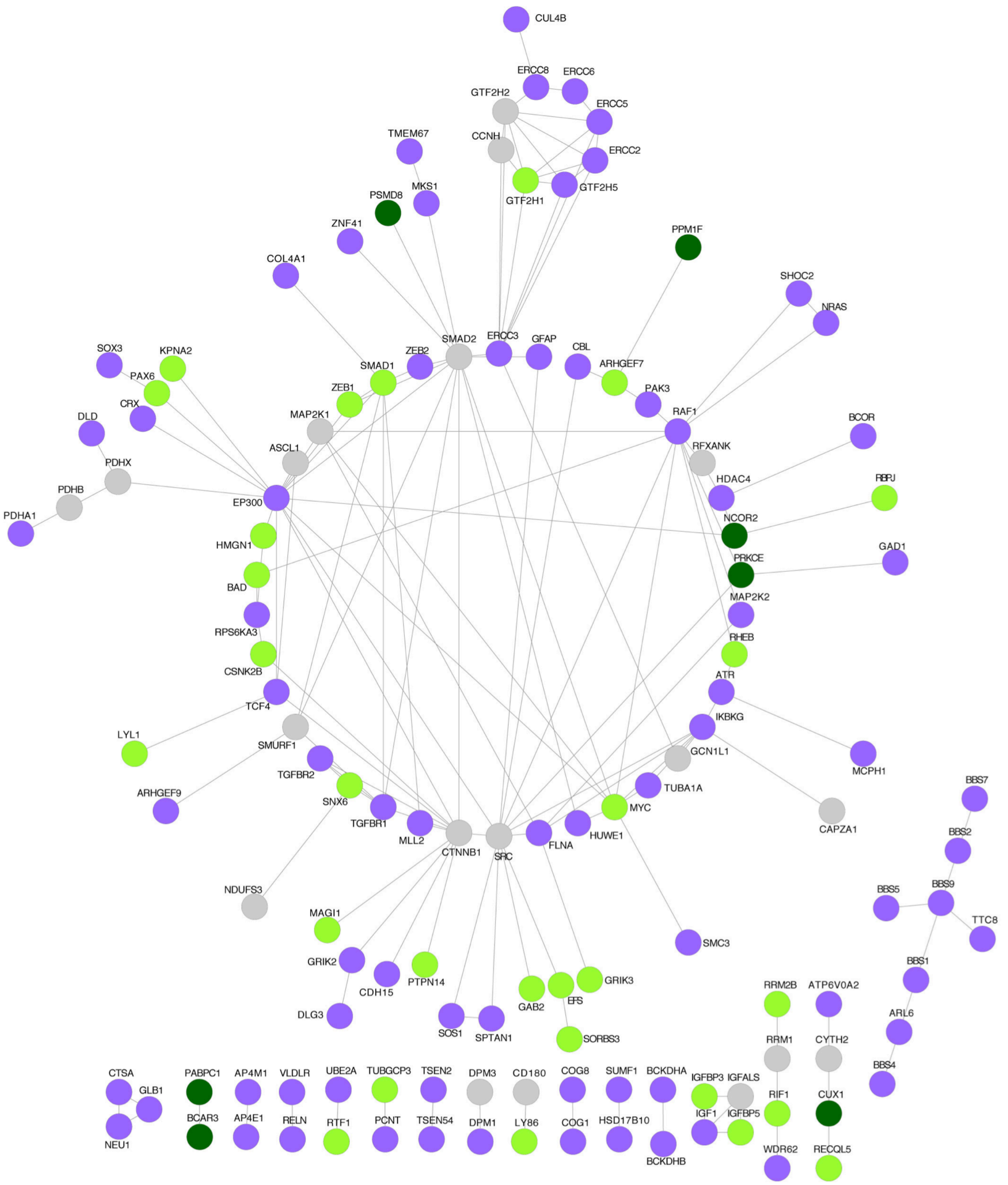

Supplemental Figure S8: PPI Network of candidate genes and intellectual disability (ID) genes

Purple nodes indicate genes from a curated list of previously implicated genes in ID. Light green and dark green nodes refer to genes from our WGCNA analysis of ASD-associated CpGs. Grey defines intermediate genes.

Supplemental Table S7: Bonferroni-corrected p-values of association between modules and known covariates

Module	ASD	Gender	Age	YRI %	CEU %
MElavenderblush2	1	1	1	1	1
Meplum3	1	1	1	1	1
Mecoral2	1	1	1	1	1
Methistle3	1	1	1	1	1
Memediumorchid	1	1	1	1	1
Medeeppink	1	1	1	1	1
Melightslateblue	1	1	1	1	1
Mepaleturquoise	1	1	1	1	1
Mepalevioletred3	1	1	1.38E-02	1	1
Mechocolate4	1	1	3.18E-04	1	1
Memediumpurple1	1	1	1	1	1
Menavajowhite1	1	1	9.65E-02	1	1
Medarkseagreen3	1	1	4.47E-02	1	1
Mepink4	1	1	7.86E-02	1	1
Mehoneydew	1	1	5.09E-04	1	1
Mesaddlebrown	0.65	1	3.67E-03	1	1
Mepalevioletred2	1	1	3.04E-03	1	1
Methistle1	2.43E-02	1	7.58E-05	1	1
Memagenta4	0.16	1	3.09E-17	1	1
Meyellow	8.50E-04	1	8.40E-16	2.53E-02	1.98E-02
Mefirebrick3	8.23E-02	1	2.31E-08	1	0.88
Mepalevioletred1	2.85E-02	1	1.73E-10	2.06E-02	1.38E-02
Meplum2	1	1	4.00E-09	1	1
Melightsteelblue1	1	1	3.61E-15	0.59	0.74
Menavajowhite2	0.21	1	5.86E-11	0.33	0.51
Mecoral3	1	1	4.13E-15	1	1
Mebrown	9.93E-03	1	9.61E-28	1.27E-02	1.40E-02
Meskyblue1	1	1	3.32E-23	1	1
Mecoral4	2.31E-02	1	2.30E-03	1	1
Mecyan	1	1	1	1	1
Metan4	5.92E-02	1	1.55E-04	1	1
Mehoneydew1	1	1	3.28E-02	1	1
Melightcoral	1	1	1.12E-04	0.23	1
Meorange	1	1	1.64E-08	9.54E-02	0.280779828
Meantiquewhite2	1	1	1	1	1
Megreen	1	1	9.28E-03	1	1
Meyellow4	1	1	4.17E-06	1	1
Meblue	1	1	3.95E-06	1	1
Meturquoise	1	1	8.25E-06	1	1
Medarkseagreen4	1	1	1	1	1
Medarkgreen	1	1	1	1	1

Mefloralwhite	1	0.92	1	6.57E-03	2.44E-04
Meorangered4	1	1	1	1	1
Meblue4	1	1	1	1	1
Megreen4	1	1	1	1	1
Melightsteelblue	1	1	1	1	1
Meskyblue3	1	1	1	1	1
Mesalmon2	1	1	1	1	1
Meskyblue2	1	1	1	1	1
Mesienna3	1	1	1	1	1
Meorangered3	1	1	1	1	1
Meplum1	1	1	1	1	1
Meyellowgreen	1	1	1	1	1
Mesienna4	1	1	1	1	1
Mesteelblue	1	1	1	1	1
Meindianred4	1	1	1	1	1
Melavenderblush1	1	1	1	1	1
Medarkslateblue	1	1	1	1	1
Meskyblue4	1	1	1	1	1
Mesalmon1	1	1	1	1	1
Mewhite	1	1	1	1	1
Memidnightblue	1	1	1	1	1
Methistle2	1	1	1	1	1
Medarkviolet	1	1	1	1	1
**Melightgreen**	**5.26E-02**	**1**	**1**	**1**	**1**
Meskyblue	1	1	1	1	1
Meyellow3	1	1	1	1	1
Medarkseagreen2	1	1	1	1	1
Meorangered1	1	1	1	1	1
Meivory	1	1	1	1	1
Medarkolivegreen	1	1	1	1	1
Memagenta3	1	1	1	1	1
Melightcyan	1	1	1	4.30E-02	0.59
Melightpink4	1	1	1	1	1
Meroyalblue	1	1	1	1	0.10
Mecoral	1	1	1	1	1
Medarkgrey	1	1	1	1	1
Memagenta	1	1	1	0.19	0.64
Melavenderblush3	1	1	1	1	1
Mefirebrick4	1	1	1	1	1
Methistle4	1	1	0.28	1	1
Medarkmagenta	1	1	1	0.67	0.24
**Medarkolivegreen2**	**1.01E-02**	**1**	**0.18**	**0.42**	**0.54**
Memediumpurple2	1	1	1	1	1
Meviolet	1	1	1	1	1

Meblack	1	1	1	5.83E-02	0.47
Megreenyellow	1	1	1	3.28E-03	3.61E-02
Memaroon	1	0.64	1	0.59	0.82
Mepurple	0.51	1.08E-42	1	1	1
Meantiquewhite4	1	1	1	1	1
Meblueviolet	1	1	1	1	1
Meplum4	1	1	1	1	1
Memediumpurple3	1	1	1	1	1
Menavajowhite	1	1	1	1	1
Mesalmon4	1	1	1	1	1
Meindianred3	1	1	1	1	1
Meblue2	1	1	1	1	1
Mecoral1	1	1	1	1	1
Meplum	1	1	1	1	1
Mered	1	1	1	5.97E-03	3.75E-02
Mesalmon	1	1	1	6.59E-02	0.39877902
Melightblue4	1	1	1	1	1
Mebrown2	0.91	1	1	1	1
Medarkturquoise	4.07E-02	1	8.32E-29	1	1
Melightyellow	3.68E-02	1	2.75E-20	1	1
Medarkorange2	1	1	6.18E-04	1	1
Metan	1	1	2.51E-09	1	1
Melightcyan1	1	1	6.23E-02	1	1
Mebisque4	1	1	1	1	1
Memediumpurple4	1	1	1	1	1
Meantiquewhite1	1	1	1.42E-03	1	1
Medarkolivegreen4	0.38	1	1.13E-05	0.99	0.47
Medarkorange	2.48E-02	1	1.26E-07	1	1
Melightpink3	1	1	1	1	1
Medarkred	1	1	1	1	1
Megrey60	1	1	1	0.19	1
Mebrown4	1	1	1	1	1
Mepink	1	1	1	2.08E-02	0.15
Melightpink2	1	1	1	1	1
Methistle	1	1	1.75E-02	1	1
Megrey	6.01E-05	1	2.81E-30	4.72E-03	3.28E-03

Supplemental Table S8: Genes in *light green* module associated with ASD

Gene	Chromosome	Position	Probe	Module Membership (MM)	MM P-value
HES4	1	936610	cg15825501	0.80	6.35E-22
ALDH4A1	1	19229528	cg01972394	0.76	9.95E-19
GRIK3	1	37498270	cg04364463	0.72	8.16E-16
DPH2	1	44435457	cg06790019	0.70	1.47E-14
EIF2B3	1	45452166	cg05654404	0.73	9.48E-17
RAD54L	1	46712932	cg16163324	0.79	1.18E-20
C1orf183	1	112281877	cg21304211	0.88	2.41E-31
ST7L	1	113162073	cg17861791	0.77	2.17E-19
CAPZA1	1	113162073	cg17861791	0.77	2.17E-19
DENND2C	1	115212659	cg13501090	0.85	8.00E-27
LGR6	1	202162209	cg04351903	0.85	5.85E-27
PTPN14	1	214725274	cg17024643	0.78	8.04E-20
MORN2	2	39103277	cg07479988	0.79	1.38E-20
DHX57	2	39103277	cg07479988	0.79	1.38E-20
MGAT5	2	134949571	cg16240751	0.76	1.67E-18
RIF1	2	152266336	cg18320648	0.80	9.07E-22
RPE	2	210867059	cg10181911	0.72	3.05E-16
IGFBP5	2	217559020	cg03222971	0.74	2.99E-17
SCAP	3	47516975	cg01374398	0.77	4.27E-19
TNNC1	3	52489346	cg01910272	0.71	3.55E-15
NISCH	3	52489346	cg01910272	0.71	3.55E-15
MAGI1	3	66024691	cg15764058	0.72	6.66E-16
NAALADL2	3	174095241	cg17014718	-0.71	1.86E-15
HES1	3	193852754	cg26348180	0.76	2.91E-18
MAN2B2	4	6577027	cg09477292	0.76	2.38E-18
HS3ST1	4	11810667	ch.4.11419765R	0.76	2.21E-18
CD38	4	15780522	cg27473538	0.73	1.31E-16
RBPJ	4	26323246	cg06812693	0.82	4.72E-23
FRAS1	4	78977690	cg12573119	0.76	1.78E-18
FRAS1	4	78978133	cg01402409	0.74	3.84E-17
COQ2	4	84205961	cg20161984	0.77	2.09E-19
LARP7	4	113626831	cg03148140	0.71	1.34E-15
SMAD1	4	146296778	cg06295548	-0.72	6.74E-16
HMGB2	4	174254825	cg14705778	0.73	1.90E-16
RGS7BP	5	63802184	cg08709073	0.78	4.86E-20
FAM151B	5	79783889	cg06506598	0.72	9.98E-16
PRDM6	5	122435202	cg01196322	0.75	5.20E-18
NEUROG1	5	134871686	cg17772342	0.72	3.06E-16
SPARC	5	151031796	cg21530174	0.72	3.59E-16
TSPAN17	5	176131088	cg13041389	-0.79	7.70E-21
ZFP62	5	180287685	cg24877792	0.80	2.45E-21
LY86	6	6648823	cg16101278	-0.70	6.61E-15

BAT4	6	31634141	cg27137280	0.70	5.78E-15
CSNK2B	6	31634141	cg27137280	0.70	5.78E-15
PPIL1	6	36842651	cg04860157	0.81	7.24E-23
CRIP3	6	43276478	cg18857062	0.74	2.91E-17
LCA5	6	80246572	cg19879479	0.84	5.79E-26
NDUFAF4	6	97345972	cg04571327	0.70	9.24E-15
C6orf174	6	127837548	cg21986718	0.75	6.74E-18
TNRC18	7	5463409	cg23209537	0.71	1.93E-15
IGFBP3	7	45961943	cg15208757	0.76	3.38E-18
TYW1B	7	72298667	cg21423973	0.72	4.62E-16
SBDSP	7	72298667	cg21423973	0.72	4.62E-16
GATSL1	7	74379144	cg18210722	-0.69	1.63E-14
LHFPL3	7	103969483	cg17373058	0.71	2.38E-15
LHFPL3	7	103970195	cg11826638	0.70	5.50E-15
TSPAN12	7	120497479	cg26340461	0.72	3.42E-16
RHEB	7	151215566	cg20495206	0.80	7.33E-22
SORBS3	8	22423994	cg21291431	0.78	1.07E-19
TNFRSF10B	8	22926800	cg26918957	0.71	2.51E-15
DPYSL2	8	26434689	cg14967899	0.72	5.60E-16
RRM2B	8	103251909	cg12374732	0.70	1.30E-14
MYC	8	128748155	cg25080152	0.73	1.62E-16
PCSK5	9	78506874	cg13512204	0.79	3.30E-21
ZEB1	10	31608136	cg25231972	0.75	1.02E-17
FZD8	10	35930499	cg00645593	0.72	3.60E-16
ZNF503-AS1	10	77054788	cg15394763	0.70	5.06E-15
FGF8	10	103535362	cg11706469	0.78	1.15E-19
ZNF215	11	6948101	cg10765857	0.80	9.54E-22
GTF2H1	11	18343657	cg11347316	0.74	4.99E-17
HPS5	11	18343657	cg11347316	0.74	4.99E-17
PAX6	11	31831591	cg16822387	0.72	7.24E-16
KBTBD4	11	47600851	cg07996345	0.73	1.87E-16
NDUFS3	11	47600851	cg07996345	0.73	1.87E-16
FADS1	11	61584442	cg25837350	0.76	2.55E-18
VEGFB	11	64002754	cg18872604	0.70	1.51E-14
BAD	11	64052221	cg25163015	0.79	6.58E-21
GPR137	11	64052221	cg25163015	0.79	6.58E-21
KRTAP5-11	11	71340352	cg22870994	-0.81	2.92E-22
GAB2	11	78129288	cg05492810	0.77	4.88E-19
TMEM126B	11	85339628	cg12830327	0.78	2.11E-20
DLG2	11	85339628	cg12830327	0.78	2.11E-20
MPZL3	11	118123074	cg27161463	0.71	2.29E-15
CLEC4C	12	7904267	cg18348303	-0.73	7.50E-17
DDX11	12	31226536	cg16864700	0.75	1.16E-17
NELL2	12	45270304	cg21846305	0.74	2.41E-17

TRHDE	12	72667326	cg09972192	0.71	2.35E-15
LOC283392	12	72667326	cg09972192	0.71	2.35E-15
N4BP2L1	13	33002431	cg06513149	0.78	2.70E-20
ABCC4	13	95953574	cg15396799	0.75	1.21E-17
ARHGEF7	13	111768023	cg03925425	0.77	5.42E-19
TUBGCP3	13	113263221	cg24531141	-0.76	2.00E-18
EFS	14	23834995	cg02213260	0.75	4.72E-18
PSME1	14	24604912	cg24054649	0.71	2.82E-15
SNX6	14	35099518	cg13093793	0.74	6.03E-17
SRP54	14	35451984	cg04980793	0.70	5.47E-15
FOXN3	14	90084672	cg26386436	0.74	4.68E-17
SLC25A29	14	100751514	cg10963192	0.75	4.31E-18
PACS2	14	105827276	cg15936935	-0.85	5.52E-27
RTF1	15	41708917	cg15581235	0.78	2.32E-20
CORO2B	15	68870836	cg12043722	0.74	1.96E-17
CSPG4	15	75986363	cg14576802	0.71	2.67E-15
ZNF200	16	3285262	cg03530756	0.74	5.77E-17
KLHDC4	16	87811505	cg09562174	0.76	2.23E-18
RNMTL1	17	685915	cg27220681	0.71	1.85E-15
GLOD4	17	685915	cg27220681	0.71	1.85E-15
NF1	17	29421732	cg02726883	0.75	4.54E-18
SLFN11	17	33700513	cg05504685	0.72	5.32E-16
COPZ2	17	46114574	cg21384971	0.79	3.32E-21
MIR152	17	46114574	cg21384971	0.79	3.32E-21
LOC146880	17	62777690	cg15869463	0.76	1.34E-18
KPNA2	17	66031814	cg13777502	0.72	8.83E-16
RECQL5	17	73629082	cg04219446	0.74	3.28E-17
LOC643008	17	73629082	cg04219446	0.74	3.28E-17
TIMP2	17	76921528	cg10466987	0.80	2.29E-21
CCDC165	18	8707237	cg14095692	0.77	1.28E-19
RAB27B	18	52495848	cg05095774	0.70	6.23E-15
CCDC102B	18	66382471	cg15552529	0.78	3.46E-20
TMX3	18	66382471	cg15552529	0.78	3.46E-20
SGTA	19	2761892	cg04171554	0.70	6.43E-15
ZNF77	19	2945000	cg04335562	0.80	5.98E-22
TNPO2	19	12833533	cg11788103	0.71	4.20E-15
LYL1	19	13213716	cg02011446	0.71	1.22E-15
UQCRFS1	19	29704262	cg02905964	0.74	6.92E-17
RTN2	19	45996498	cg19869610	0.74	6.33E-17
SMOX	20	4129314	cg20604317	0.73	1.77E-16
HMGN1	21	40720919	cg01338834	0.73	9.04E-17
DGCR6	22	18893614	cg07004357	0.71	1.42E-15
LZTR1	22	21337040	cg07047601	0.81	2.81E-22
PNPLA5	22	44287772	cg24258125	0.77	1.49E-19

Supplemental Table S9: Genes in *dark olive green* module associated with ASD

Gene	Chromosome	Position	Probe	Module Membership (MM)	MM P-value
PEX10	1	2337334	cg16523185	0.810337844	1.32E-22
GPBP1L1	1	46106399	cg17827803	0.773502529	1.61E-19
BCAR3	1	94245226	cg24937735	0.740245636	3.41E-17
ZC3H11A	1	203763483	cg16020436	0.768457607	3.85E-19
ZC3H11A	1	203763498	cg02337583	0.699133215	9.15E-15
CAMKMT	2	44646510	cg10521567	0.830944239	1.21E-24
PRKCE	2	46121488	cg16884847	0.728533494	1.86E-16
RBMS1	2	161593271	cg13392885	0.776332535	9.80E-20
ARPC2	2	219079038	cg12884009	0.797400033	1.89E-21
BSN	3	49638532	cg22881573	0.845041711	3.35E-26
CPLX1	4	795538	cg02133849	0.702505454	5.99E-15
NFXL1	4	47854334	cg27531236	0.802345605	7.00E-22
C5orf27	5	95192949	cg26898099	0.70421452	4.83E-15
ERGIC1	5	172263112	cg24581650	0.683902398	5.75E-14
TAP2	6	32803058	cg00386460	0.778753197	6.37E-20
COL11A2	6	33151008	cg07457375	0.729478157	1.63E-16
WDR46	6	33254880	cg23652681	0.801745369	7.91E-22
WDR46	6	33254892	cg17417645	0.761165479	1.30E-18
RSPH3	6	159423743	cg15999887	0.769393089	3.28E-19
AGPAT4	6	161549519	cg26780581	0.790267496	7.57E-21
MACC1	7	20240145	cg21710826	0.698674927	9.68E-15
CUX1	7	101478535	cg08582182	0.802229538	7.17E-22
JHDM1D	7	139859464	cg26800802	0.713418296	1.47E-15
KIAA0146	8	48557420	cg04247508	0.792246082	5.18E-21
KIAA0146	8	48587440	cg18404513	0.753484619	4.48E-18
PABPC1	8	101802144	cg08995449	0.774123593	1.45E-19
ARFIP2	11	6499575	cg17403702	0.736923523	5.57E-17
MICAL2	11	12222570	cg09371112	0.722898685	4.08E-16
CNIH2	11	66048759	cg06155341	0.805030303	4.03E-22
MAML2	11	95889454	cg15521790	0.781796778	3.67E-20
KRT79	12	53228661	cg13119928	0.764105173	8.00E-19
NCOR2	12	125030744	cg07243762	0.722336993	4.41E-16
ATP11A	13	113348391	cg09507215	0.730182655	1.47E-16
HOMEZ	14	23744304	cg04420752	0.747121013	1.21E-17
ARHGAP5	14	32597733	cg07564690	0.831457161	1.07E-24
RBM25	14	73524288	cg04422024	0.757192347	2.48E-18
c15orf50	15	70147094	cg16497945	0.763106126	9.44E-19
MLYCD	16	83945978	cg01984843	0.776011546	1.04E-19
ZNF426	19	9645566	cg21474288	0.749726963	8.08E-18
PSMD8	19	38869646	cg11607742	0.801710907	7.97E-22
HM13	20	30126382	cg04306926	0.728729016	1.81E-16
PPM1F	22	22290866	cg01800253	0.710229687	2.23E-15

Code

Using R version 2.15.0

```
#LOADING IN RAW IDAT FILES
setwd("INSERT USER DIRECTORY")
man<-read.csv("HumanMethylation450_15017482_v.1.1.csv", skip=7, header=TRUE,
stringsAsFactors=FALSE)

require(minfi)
library(IlluminaHumanMethylation450kmanifest)
targets <- read.450k.sheet("USER DIRECTORY", pattern="pheno96.csv")
Basename<-apply(targets, 1, function(x){
	paste("USER DIRECTORY", x[13], "/", x[13], "_", x[12], sep="")
})
targets<-data.frame(targets[,1:13], Basename)
RGset <- read.450k.exp(base = "USER DIRECTORY", targets = targets)

##Extract bisulphite control values from getProbeInfo from RGset

#CALCULATE DETECTION PVALS TO REMOVE POORLY PERFORMING PROBES
sampleNames(RGset) = targets$Sample_Name
pVals = detectionP(RGset)

M.set<-preprocessRaw(RGset)  #this is what the table of signal intensities is extracted from

#REMOVE SEX CHROMOSOMES BASED ON ILLUMINA MANIFEST ANNOTATION
cpgs<-getManifestInfo(M.set, type = c("locusNames"))
Y<-which(man[,12]=="Y")
X<-which(man[,12]=="X")
cpgsremove<-c(man[X,1], man[Y,1])
pos<-cpgs %in% cpgsremove
nomatch<-which(pos=="FALSE")
M.set2<-M.set[nomatch,]

#NORMALIZE AUTOSOME DATA WITH SWAN
M.setswan<-preprocessSWAN(RGset, mSet=M.set2)

#REMOVE PROBES WITH DETECTION P-VAL > 0.01
pValsauto<-pVals[nomatch,]
msetswanfilt<-M.setswan[rowSums(pValsauto)<=0.01,]

#EXTRACT M-VALUES FROM NORMALIZED, FILTERED DATASET
mvals<-getM(msetswanfilt, type="Illumina")
write.table(mvals, file="m_values.txt", sep="\t", quote=FALSE)

#CORRECT FOR BATCH EFFECT
library(sva)
pd<-pData(msetswanfilt)
```

```
batch<-pd$Slide
mod<-model.matrix(~as.factor(Gender) + age.3.2012 + as.factor(Sample_Group), data=pd)
norm_m<-ComBat(mvals, batch=batch, mod=mod, numCovs=3,
par.prior=TRUE,prior.plots=FALSE)
write.table(norm_m, file="norm_m.txt", sep="\t", quote=FALSE)

##This file contains 96 individuals.  Three were then removed from subsequent DMR analysis
(one had a changed diagnosis, and two did not have genotyping data as their genotyping arrays
failed quality controls.  For simplicity, the phenotype file with the 93 retained individuals is
provided for the subsequent analysis.

#BUMPHUNTING ANALYSIS

setwd("USER DIRECTORY")
mval<-read.delim("norm_m.txt", header=TRUE, stringsAsFactors=FALSE)

cpgnames<-row.names(mval)

manifest<-read.csv("HumanMethylation450_15017482_v.1.1.csv", skip=7, sep=",",
stringsAsFactors=FALSE)
temp<-match(cpgnames, manifest[,1])
manifest2<-manifest[temp,]

pheno<-read.csv("pheno93.csv", stringsAsFactors=FALSE)

names<-colnames(mval)
keep<-match(pheno[,1], names)
mval2<-mval[,keep]

chr<-manifest2[,12]
chr<-paste("chr", chr, sep="")
position<-as.numeric(manifest2[,13])
library(charm)

#this part reorders each chromosome by position and then rbinds them all together, and then
reorders the matrix of m-values by that order too)
a<-cbind(chr, position, cpgnames)
chrs<-levels(as.factor(chr))
final<-c()
for (j in 1:length(chrs)){
b<-subset(a, a[,1]==chrs[j])
ord<-order(as.numeric((b[,2])))
c<-b[ord,]
final<-rbind(final, c)
}
exclude<-which(final[,1]=="chr")
final2<-final[-exclude,]
d<-match(final2[,3], row.names(mval))
mval3<-mval2[d,]
```

```
position<-as.numeric(final2[,2])
chr<-final2[,1]

pns<-clusterMaker(chr, position, order.it=TRUE, maxGap=300)

mod<-model.matrix(~as.factor(pheno[,8]) + (pheno[,9]) + (pheno[,10]) + pheno[,11] +
as.factor(Sample_Group), data=pheno)
mod0<-model.matrix(~as.factor(pheno[,8]) + (pheno[,9]) + (pheno[,10]) + pheno[,11],
data=pheno)

mval4<-data.matrix(mval3)
test<-dmrFind(logitp=mval4, mod=mod, mod0=mod0, coeff=6, pns=pns, chr=chr, pos=position)

## TYPE test$dmrs for dmrs

##WGCNA CODE IS FREELY AVAILABLE AT
```

http://labs.genetics.ucla.edu/horvath/CoexpressionNetwork/Rpackages/WGCNA/. Parameters used in the application of this package are explained in detail in the Supplemental Materials.

Supplemental References

Aryee MJ, Wu Z, Ladd-Acosta C, Herb B, Feinberg AP, Yegnasubramanian S, Irizarry RA. 2011. Accurate genome-scale percentage DNA methylation estimates from microarray data. *Biostatistics* **12**(2): 197-210.

Basu SN, Kollu R, Banerjee-Basu S. 2009. AutDB: a gene reference resource for autism research. *Nucleic Acids Res* **37** (Database issue): D832-836.

Colella S, Yau C, Taylor JM, Mirza G, Butler H, Clouston P, Bassett AS, Seller A, Holmes CC, Ragoussis J. 2007. QuantiSNP: an Objective Bayes Hidden-Markov Model to detect and accurately map copy number variation using SNP genotyping data. *Nucleic Acids Res* **35**(6): 2013-2025.

Conlin LK, Thiel BD, Bonnemann CG, Medne L, Ernst LM, Zackai EH, Deardorff MA, Krantz ID, Hakonarson H, Spinner NB. 2010. Mechanisms of mosaicism, chimerism and uniparental disomy identified by single nucleotide polymorphism array analysis. *Hum Mol Genet* **19**(7): 1263-1275.

Danecek P, Auton A, Abecasis G, Albers CA, Banks E, DePristo MA, Handsaker RE, Lunter G, Marth GT, Sherry ST et al. 2011. The variant call format and VCFtools. *Bioinformatics* **27**(15): 2156-2158.

Dedeurwaerder S, Defrance M, Calonne E, Denis H, Sotiriou C, Fuks F. 2011. Evaluation of the Infinium Methylation 450K technology. *Epigenomics* **3**(6): 771-784.

Erten S, Bebek G, Ewing RM, Koyuturk M. 2011. DADA: Degree-Aware Algorithms for Network-Based Disease Gene Prioritization. *BioData Min* **4**: 19.

Geeleher P, Hartnett L, Egan LJ, Golden A, Raja Ali RA, Seoighe C. 2013. Gene-set analysis is severely biased when applied to genome-wide methylation data. *Bioinformatics* **29**(15): 1851-1857.

Gonzalez JR, Rodriguez-Santiago B, Caceres A, Pique-Regi R, Rothman N, Chanock SJ, Armengol L, Perez-Jurado LA. 2011. A fast and accurate method to detect allelic genomic imbalances underlying mosaic rearrangements using SNP array data. *BMC Bioinformatics* **12**: 166.

Hansen KD, Aryee M. minfi: Analyze Illumina's 450k methylation arrays., Vol version 1.4.0, p. R package. BioConductor.

Kent WJ, Sugnet CW, Furey TS, Roskin KM, Pringle TH, Zahler AM, Haussler D. 2002. The human genome browser at UCSC. *Genome Res* **12**(6): 996-1006.

Lachmann A, Ma'ayan A. 2010. Lists2Networks: integrated analysis of gene/protein lists. *BMC Bioinformatics* **11**: 87.

Langfelder P, Horvath S. 2008. WGCNA: an R package for weighted correlation network analysis. *BMC Bioinformatics* **9**: 559.

Leek JT, E. JW, Parker HS, E. JA, Storey JD. sva: Surrogate Variable Analysis., Vol version 3.2.1, p. R package. BioConductor.

Maksimovic J, Gordon L, Oshlack A. 2012. SWAN: Subset-quantile within array normalization for illumina infinium HumanMethylation450 BeadChips. *Genome Biol* **13**(6): R44.

Neale BM, Kou Y, Liu L, Ma'ayan A, Samocha KE, Sabo A, Lin CF, Stevens C, Wang LS, Makarov V et al. 2012. Patterns and rates of exonic de novo mutations in autism spectrum disorders. *Nature* **485**(7397): 242-245.

Price AL, Tandon A, Patterson N, Barnes KC, Rafaels N, Ruczinski I, Beaty TH, Mathias R, Reich D, Myers S. 2009. Sensitive detection of chromosomal segments of distinct ancestry in admixed populations. *PLoS Genet* **5**(6): e1000519.

Rebhan M, Chalifa-Caspi V, Prilusky J, Lancet D. 1997. GeneCards: integrating information about genes, proteins and diseases. *Trends Genet* **13**(4): 163.

Sanders SJ, Ercan-Sencicek AG, Hus V, Luo R, Murtha MT, Moreno-De-Luca D, Chu SH, Moreau MP, Gupta AR, Thomson SA et al. 2011. Multiple recurrent de novo CNVs, including duplications of the 7q11.23 Williams syndrome region, are strongly associated with autism. *Neuron* **70**(5): 863-885.

Voineagu I, Wang X, Johnston P, Lowe JK, Tian Y, Horvath S, Mill J, Cantor RM, Blencowe BJ, Geschwind DH. 2011. Transcriptomic analysis of autistic brain reveals convergent molecular pathology. *Nature* **474**(7351): 380-384.

Warde-Farley D, Donaldson SL, Comes O, Zuberi K, Badrawi R, Chao P, Franz M, Grouios C, Kazi F, Lopes CT et al. 2010. The GeneMANIA prediction server: biological network integration for gene prioritization and predicting gene function. *Nucleic Acids Res* **38** (Web Server issue): W214-220.

Xi Y, Li W. 2009. BSMAP: whole genome bisulfite sequence MAPping program. *BMC Bioinformatics* **10**: 232.

Xu LM, Li JR, Huang Y, Zhao M, Tang X, Wei L. 2012. AutismKB: an evidence-based knowledgebase of autism genetics. *Nucleic Acids Res* **40** (Database issue): D1016-1022.

Young MD, Wakefield MJ, Smyth GK, Oshlack A. 2010. Gene ontology analysis for RNA-seq: accounting for selection bias. *Genome Biol* **11**(2): R14.
