# Supplementary material for: Mosaic Epigenetic Dysregulation of Ectodermal Cells in Autism Spectrum Disorder
Source: PLoS Genet. 2014 May 29;10(5):e1004402. doi: 10.1371/journal.pgen.1004402 (PMC4038484; doi:10.1371/journal.pgen.1004402)
Supplement: Figure S8 — PPI Network of candidate genes and intellectual disability (ID) genes. Purple nodes indicate genes from a curated list of previously implicated genes in ID. Light green and dark green nodes refer to genes from our WGCNA analysis of ASD-associated CpGs. Grey defines intermediate genes. (PDF) [file pgen.1004402.s008.pdf]

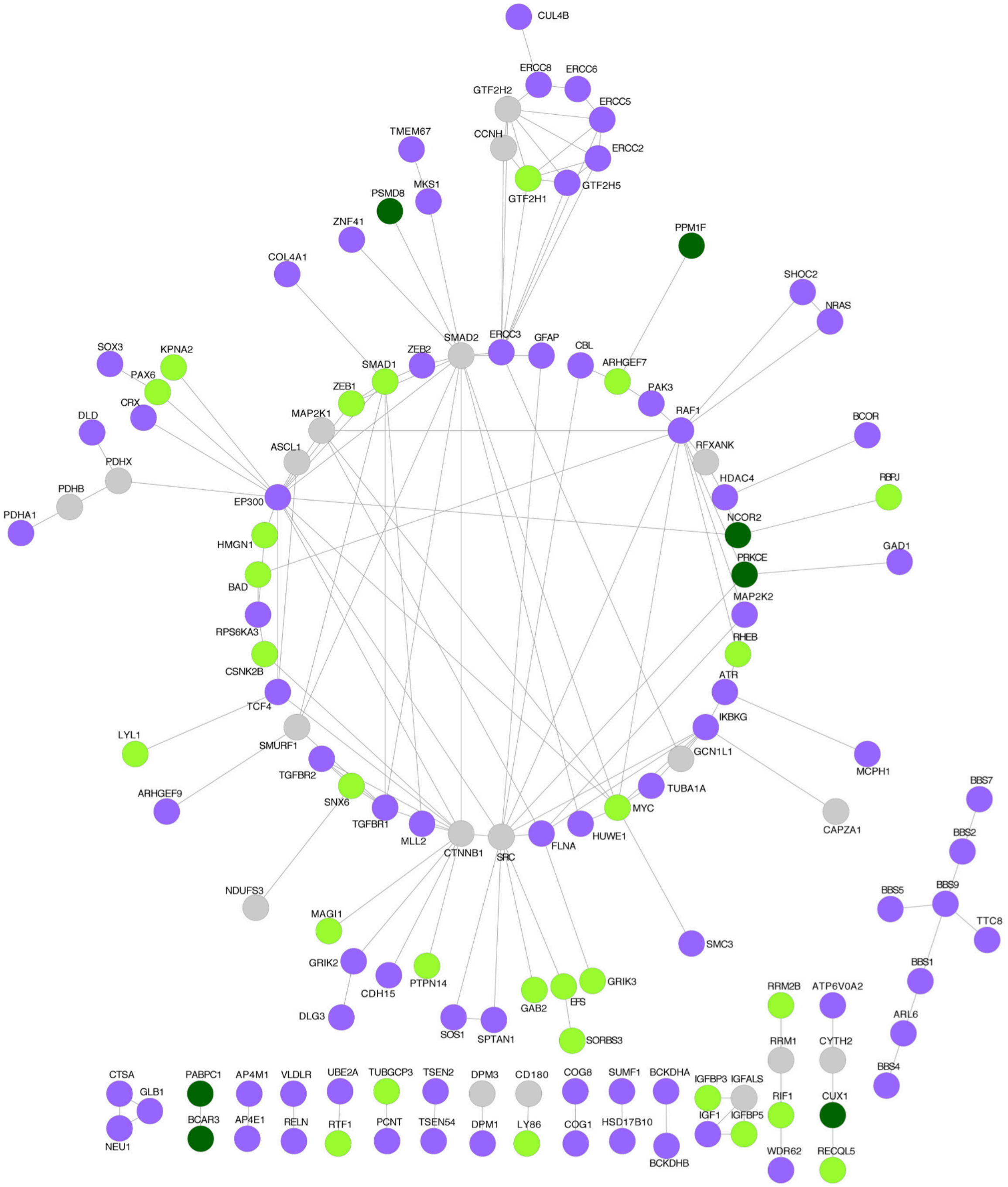

Supplemental Figure S8: PPI Network of candidate genes and intellectual disability (ID) genes

Purple nodes indicate genes from a curated list of previously implicated genes in ID. Light green and dark green nodes refer to genes from our WGCNA analysis of ASD-associated CpGs. Grey defines intermediate genes.
